# Optimizations of lipid II synthesis: an essential glycolipid precursor in bacterial cell wall synthesis and a validated antibiotic target

**DOI:** 10.3762/bjoc.20.22

**Published:** 2024-02-06

**Authors:** Milandip Karak, Cian R Cloonan, Brad R Baker, Rachel V K Cochrane, Stephen A Cochrane

**Affiliations:** 1 School of Chemistry and Chemical Engineering, Queen's University Belfast, David Keir Building, Stranmillis Road, Belfast, BT9 5AG, UK

**Keywords:** chemical glycosylation, lipid II, peptidoglycan, polyprenyls, total synthesis

## Abstract

Lipid II is an essential glycolipid found in bacteria. Accessing this valuable cell wall precursor is important both for studying cell wall synthesis and for studying/identifying novel antimicrobial compounds. Herein, we describe optimizations to the modular chemical synthesis of lipid II and unnatural analogues. In particular, the glycosylation step, a critical step in the formation of the central disaccharide unit (GlcNAc-MurNAc), was optimized. This was achieved by employing the use of glycosyl donors with diverse leaving groups. The key advantage of this approach lies in its adaptability, allowing for the generation of a wide array of analogues through the incorporation of alternative building blocks at different stages of synthesis.

## Introduction

**Figure 1:**
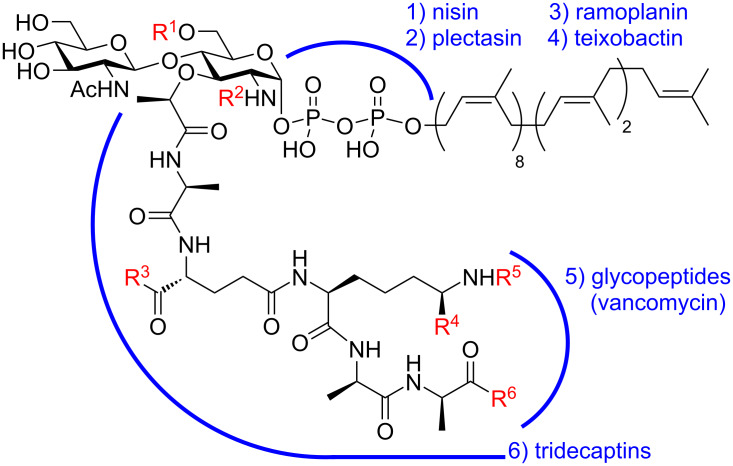
Structure of lipid II, with variable positions shown in red and antimicrobial-binding motifs highlighted with blue arcs. R^1^ = H or Ac; R^2^ = H or Ac; R^3^ = OH, OMe or NH_2_; R^4^ = H or COOH; R^5^ = Gly_5_, Ala_2_, Ala-Ser/Ala or ᴅ-Asp; R^6^ = OH, OMe or NH_2_. These structural modifications are described in detail by Münch and co-workers [9]. For more details on lipid II-binding antimicrobials, see recent review by Buijs and co-workers [2].

Lipid II (Figure 1) is an essential bacterial glycolipid involved in peptidoglycan biosynthesis [1]. It is synthesized on the inner leaflet of the cytoplasmic membrane, before translocation to the outer leaflet, where it is then used as the monomeric building block of peptidoglycan biosynthesis. Lipid II is a validated antibiotic target for clinically prescribed antibiotics including vancomycin and ramoplanin [2]. It is also the target for a host of other antimicrobials (mostly non-ribosomal peptides), including the tridecaptins [3], nisin [4], teixobactin [5], clovibactin [6], malacidin [7], and cilagicin [8].

Despite significant progress in the chemical synthesis of lipid II and its analogues, the scarcity of these compounds and their limited structural diversity present significant obstacles to in-depth explorations of their intricate structural and functional characteristics. This scarcity issue is further exacerbated by an overwhelming demand that far exceeds existing supply capacities. To date, the chemical, chemoenzymatic, or biochemical synthesis of lipid II and its variants has been achieved by several research groups [10–27]. Nonetheless, considering the current state of knowledge, the chemical synthesis approach emerges as a more viable strategy in contrast to other methodologies, as it offers the potential to generate ample quantities of lipid II analogues suitable for high-throughput screening endeavors. In recent years, a major focus of the Cochrane lab has been the chemical synthesis of bacterial polyprenyls to study the mechanism of action of antimicrobial peptides that kill bacteria through binding to these polyprenyls [21,28–34]. Lipid II has been of particular interest, and during our synthesis of multiple different lipid II analogues, we have developed several optimizations, which we describe herein. The base lipid II syntheses upon which optimizations were made are our previously reported syntheses of Gram-negative lipid II in 2016 [20] and Gram-positive lipid II (**11**) in 2018 [23]. Building upon these synthetic strategies we have achieved noteworthy enhancements in glycosylation conditions, including improvements in reaction time and yields. This approach enables the systematic assembly of lipid II and analogues that contain shorter polyprenyl chains, specifically farnesyl (C_15_), geranylgeranyl (C_20_), and solanesyl (C_45_). Such short chain analogues are valuable in several applications due to their improved solubility in aqueous systems. Assembly is achieved by integrating distinct carbohydrate, peptide, and polyprenyl phosphate building blocks. This modular synthetic method allows for the strategic substitution of constituent building blocks at different synthetic stages and provides a practical avenue for producing substantial amounts of lipid II analogues. Consequently, this approach offers a more feasible means of addressing the demands associated with biophysical screening pursuits.

**Figure 2:**
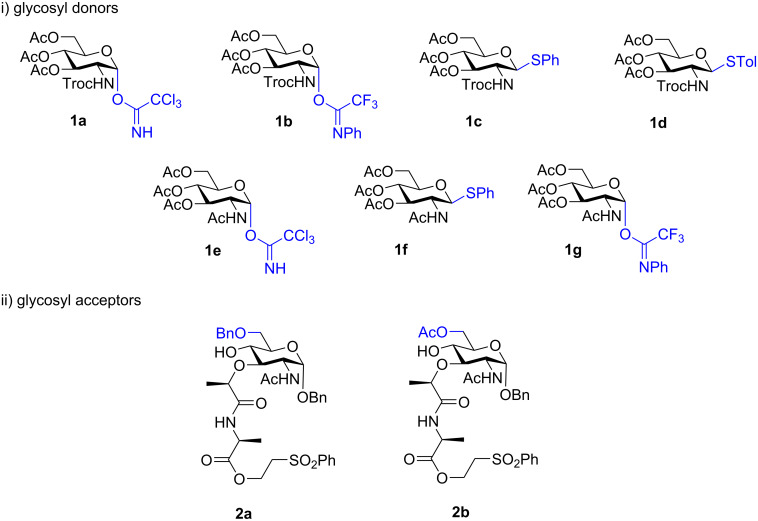
List of i) glycosyl donors and ii) glycosyl acceptors used in this study.

Prior research in the field of total synthesis of lipid II has elucidated that specific combinations of protecting groups on glycosyl acceptors and donors, as represented by compounds **1a** and **2a** in Figure 2, are proficient in the efficient generation of lipid II disaccharide [35–36]. Subsequently, significant endeavors have been directed towards the exploration of glycosyl donors, such as *N*-phthaloyl 3,4,6-*O*-triacetyl-2-deoxy-2-amino-ᴅ-glucopyranosyl-1-bromide, *N*-2,2,2-trichloroethoxycarbonyl-3,4,6-*O*-triacetyl-2-deoxy-2-amino-ᴅ-glucopyranosyl-1-bromide, and *N*-phthaloyl-2-deoxy-2-amino-3,4,6-*O*-triacetate-ᴅ-glucopyranosyl-1-(2,2,2-trichloroacetoimidate), all of which have proven successful in disaccharide synthesis alongside C6-protected acceptors (**2a** or **2b** in Figure 2) [10–11,14–15,37–38]. More recently, an innovative one-pot glycosylation approach using a (2,6-dichloro-4-methoxyphenyl)(2,4-dichlorophenyl)-protected glycosyl acceptor has been developed, demonstrating satisfactory stability under Schmidt glycosylation conditions [18]. In general, the outcome of glycosylation hinges on the specific pairing of glycosyl donors and glycosyl acceptors employed in the reaction. Notably, when glycosyl donors such as **1e**–**g**, featuring acyl group protection at the C2 position, are combined with acceptors like **2b**, which have acyl groups protecting the C6 position, the reaction kinetics become sluggish, resulting in low conversion rates or no conversion [36,39].

## Results and Discussion

In our studies, the initial glycosyl donors and acceptors (Figure 2; compounds **1a**–**g** and **2a**,**b**) were synthesized using established procedures from the literature, commencing with ᴅ-glucosamine and benzyl 2-acetamido-4,6-*O*-benzylidene-2-deoxy-α-ᴅ-glucopyranoside as the starting materials, respectively [40–43]. Imidate donors **1a** and **1e** were obtained exclusively as α-anomers, and **1b** and **1g** as a 1:1 α:β mixture which were then purified to give the desired α-anomers. Thioglycosides **1c**, **1d**, and **1f** were isolated purely as β-anomers due to anchimeric assistance from the C2 *N*-acetyl or *N*-Troc groups. In glycosyl acceptors, the first amino acid of the lipid II pentapeptide, Ala, was incorporated as a 2-(phenylsulfonyl)ethyl ester, as previously reported by Saha and co-workers [44]. This modification prevents a deleterious side reaction occurring, wherein during glycosylation, muramic acid esters undergo a 6-*exo*-*trig* cyclization with the 4-OH group. Comprehensive experimental protocols detailing the preparation of these glycosyl donors can be found in Supporting Information File 1.

Next, we conducted an extended investigation into glycosylation, employing a diverse range of glycosyl donors (**1a**–**g**) and acceptors (**2a** and **2b**), and the comprehensive results are presented in Table 1. Initially, our approach was guided by the established protocols of Kurosu et al., which had previously demonstrated effectiveness in glycosylating glycosyl trichloroacetimidate **1a** and C6-benzylated MurNAc derivative **2a** [18]. Despite our efforts to optimize the yield of the target product **3a**, involving modifications to reaction conditions such as transitioning from 0 °C to room temperature and extending the reaction duration from 3 to 24 hours, we did not observe the anticipated enhancements (51% yield, entry 1, Table 1). This trend persisted when we attempted glycosylation between C6-acetylated MurNAc derivative **2b** and **1a**, where the desired product **3b** remained elusive (Table 1, entry 2). In fact, glycosyl acceptor **2b** failed to yield the desired glycosylation product **3d** under the conditions tested (Table 1, entries 7 and 8). Moderate yields of **3a** were achieved when using glycosyl donors such as **1b**–**d** under standard conditions A or B (Table 1, entries 3–5). Notably, both Troc-protected thio-donors **1c**,**d** exhibited similar behavior in terms of yield. Unfortunately, no target product **3c** was obtained under standard glycosylation conditions A or B when C2-acetamido glycosyl donors (e.g., **1e**–**g**) were subjected to the glycosylation reaction (Table 1, entries 6, 8, and 9). A slight improvement in the yield of **3a** was observed when switching from TMSOTf to TfOH as the activator (Table 1, entry 5 vs entry 10). However, substituting TMSOTf with BF_3_·OEt_2_ did not yield any target product **3a** (Table 1, entry 3 vs entry 12). In our observations, we initially noted that at room temperature, the degradation rate of glycosyl donor **1a** exceeded the rate of product formation. This led to a complex mixture consisting of the target product **3a**, acceptor **2a**, and various degraded products of donor **1a**. This situation posed challenges, as even prolonged reaction times did not enhance the product yield, and the subsequent purification of the target product became a difficult task. However, when we conducted the reaction at lower temperatures, the degradation of glycosyl donor **1a** slowed down, and the reaction proceeded at a moderate rate. Eventually, we found that the utilization of extra equivalents of **1a** and activators, following conditions akin to those employed by Kurosu, resulted in a significant boost in the yield of the target product to 68% (Table 1, entry 11).

**Table 1:** Optimization of the glycosylation conditions.^a^

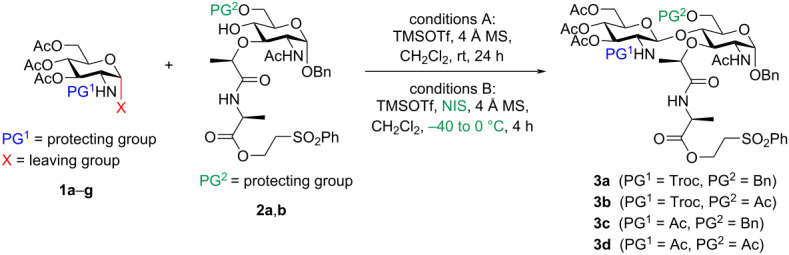					

Entry	Donor	Acceptor	Deviation from std. conditions	Product	Yield (%)

1	**1a**	**2a**	conditions A	**3a**	51
2	**1a**	**2b**	conditions A	**3b**	0
3	**1b**	**2a**	conditions A	**3a**	29
4	**1c**	**2a**	conditions B	**3a**	46
5	**1d**	**2a**	conditions B	**3a**	43
6	**1e**	**2a**	conditions A	**3c**	0
7	**1e**	**2b**	conditions A	**3d**	0
8	**1f**	**2b**	conditions B	**3d**	0
9	**1g**	**2a**	conditions A	**3c**	0
10	**1d**	**2a**	**TfOH**, NIS, 4 Å MS, CH_2_Cl_2_, −40 to 0 °C, 4 h	**3a**	50
11	**1a**	**2a**	TMSOTf, 4 Å MS, CH_2_Cl_2_, 0 °C, 3 h; **then, added 2 equiv 1a, 1 equiv TMSOTf, 0 °C, 4 h**	**3a**	68
12	**1b**	**2a**	**BF_3_·OEt_2_**, 4 Å MS, CH_2_Cl_2_, 0 °C to rt, 24 h	**3a**	0

^a^TMSOTf: trimethylsilyl trifluoromethanesulfonate, MS: molecular sieves, NIS: *N*-iodosuccinimide, Ac: acetyl, Bn: benzyl, Troc: 2,2,2-trichloroethoxycarbonyl.

**Scheme 1:**
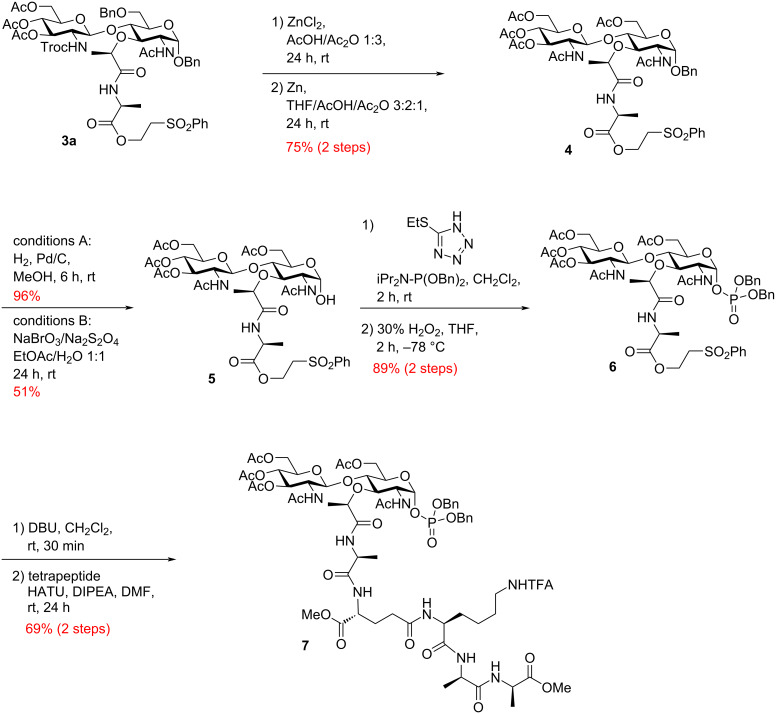
Synthesis of disaccharide pentapeptide core **7**.

Next, a comprehensive synthetic strategy for the preparation of α-phosphoryl GlcNAc-MurNAc-pentapeptide **7**, based on established protocols with minor adjustments was completed (Scheme 1) [10–11]. After the successful glycosylation reaction, disaccharide **3a**, protected with C2-Troc and C6-benzyl groups, was efficiently deprotected under acidic conditions using ZnCl_2_/Zn, followed by in situ re-acetylation of the C2-amino group and C6-alcohol with acetic anhydride, resulting in the formation of disaccharide **4** in a one-pot fashion. The anomeric benzyl protecting group in disaccharide **4** was then removed via a Pd/C-catalyzed hydrogenation reaction, producing a mixture of α/β-anomers of compound **5**. It is noteworthy to mention that the benzyl ether in compound **4** exhibited successful cleavage upon treatment with sodium bromate/sodium dithionite in ethyl acetate/water, while other protecting functionalities like acetyl and phenylsulfonylethyl ester groups remained intact [45]. The ratio of α/β-anomers in compound **5** was found to be influenced by the reaction conditions, consistently favoring the β-anomer. Further transformation of compound **5** involved α-selective phosphite formation using dibenzyl *N*,*N*-diisopropylphosphoramidite and 5-(ethylthio)-1*H*-tetrazole. The resulting α-phosphite intermediate was then oxidized with hydrogen peroxide to yield dibenzyl α-phosphate **6**, achieving an overall yield of 89% for these two steps. Removal of the 2-(phenylsulfonyl)ethanol protecting group in compound **6** was successfully achieved through treatment with 1,8-diazabicyclo[5.4.0]undec-7-ene, leading to the formation of the α-phosphoryl GlcNAc-MurNAc-monopeptide derivative. Subsequently, coupling this intermediate with tetrapeptide, TFA·H-ʟ-Ala-γ-ᴅ-Glu(OMe)-ʟ-Lys(COCF_3_)-ᴅ-Ala-ᴅ-Ala-OMe under mild conditions resulted in the synthesis of dibenzyl α-phosphoryl GlcNAc-MurNAc-pentapeptide **7** (see Supporting Information File 1 for comprehensive information on the synthesis details of the tetrapeptide). To avoid loss of valuable material through HPLC purification, crude **7** is used directly in the next step, and purification performed after the final prenyl phosphate coupling and global deprotection.

**Scheme 2:**
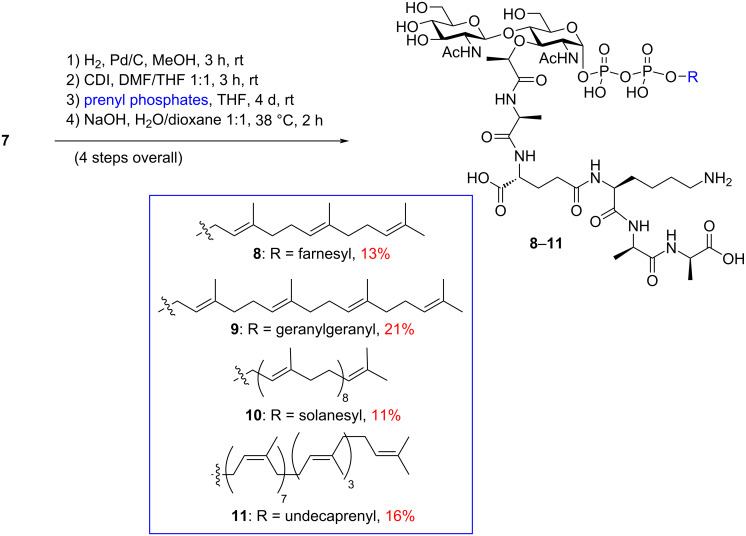
Synthesis of lipid II (**11**) and its analogues **8**–**10**.

Finally, the benzyl-protecting groups in compound **7** were cleaved via hydrogenolysis, followed by co-evaporation of the resulting crude product in pyridine. This yielded a monopyridyl salt, setting the stage for the final lipid coupling and deprotection sequence. To establish the vital lipid diphosphate linkage, we employed the phosphoroimidazolidate method, as previously utilized in other lipid II total syntheses [10–11]. The monopyridyl α-phosphoryl GlcNAc-MurNAc-pentapeptide was activated with CDI, with excess CDI being neutralized using anhydrous methanol. The resulting phosphoroimidazolidate mixture underwent a cross-coupling reaction with prenyl monophosphates [46] in DMF/THF over a four-day period, yielding fully protected versions of lipid II and its analogues. Subsequent global deprotection reactions, using aqueous NaOH, led to the formation of lipid II (**11**), with an overall yield of 16% (from compound **7**) following reversed-phase HPLC purification (Scheme 2). Similarly, farnesyl, geranylgeranyl, and solanesyl-lipid II analogues **8**–**10** were synthesized with overall yields of 13%, 21%, and 11%, respectively, using the corresponding prenyl phosphates (Scheme 2).

## Conclusion

In conclusion, we have successfully optimized a modular approach for the synthesis of lipid II and its analogues, including variants with distinct prenyl-chain lengths. The key to this methodology lies in the optimization of glycosylation conditions, utilizing readily available glycosyl donors, which is a pivotal step in constructing the central disaccharide unit. The adaptability of our method is showcased through the generation of new lipid II analogues, such as geranylgeranyl and solanesyl lipid II analogues, which involve the incorporation of distinct prenyl monophosphates during the final phases of the synthesis. Thus, this strategy holds considerable promise for advancing the synthesis of a diverse range of lipid II analogues, opening avenues for further exploration into their biophysical characteristics, as well as their interactions with antibiotics.

## Supporting Information

File 1 Experimental procedures, characterization data, and selected copies of ^1^H, ^13^C, and ^31^P NMR spectra.

## Data Availability

The data that supports the findings of this study is available from the corresponding author upon reasonable request.
