# Supplementary material for: Optimizations of lipid II synthesis: an essential glycolipid precursor in bacterial cell wall synthesis and a validated antibiotic target
Source: Beilstein J Org Chem. 2024 Feb 6;20:220–7. doi: 10.3762/bjoc.20.22 (PMC10862138; doi:10.3762/bjoc.20.22)
Supplement: File 1 — Experimental procedures, characterization data, and selected copies of 1H, 13C, and 31P NMR spectra. [file Beilstein_J_Org_Chem-20-220-s001.pdf]

BEILSTEIN JOURNAL OF ORGANIC CHEMISTRY

Supporting Information

for

Optimizations of lipid II synthesis: an essential glycolipid precursor in bacterial cell wall synthesis and a validated antibiotic target

Milandip Karak, Cian R. Cloonan, Brad R. Baker, Rachel V. K. Cochrane and Stephen A. Cochrane

Beilstein J. Org. Chem. **2024,** *20,* 220–227. doi:10.3762/bjoc.20.22

Experimental procedures, characterization data, and selected copies of ^{1}H, ^{13}C, and ^{31}P NMR spectra

License and Terms: This is a supporting information file under the terms of the Creative Commons Attribution License (https://creativecommons.org/licenses/by/4.0). Please note that the reuse, redistribution and reproduction in particular requires that the author(s) and source are credited and that individual graphics may be subject to special legal provisions.
The license is subject to the *Beilstein Journal of Organic Chemistry* terms and conditions: (https://www.beilstein-journals.org/bjoc/terms)

Table of contents

General experimental information.......... S3

Experimental procedures and characterization of products.......... S5

*2-Deoxy-2-[[(2,2,2-trichloroethoxy)carbonyl]amino]-3,4,6-triacetyl-1-(2,2,2-trichloroacetimidoyl)-α-D-glucopyranoside (**1a**)*.......... S5

*2-Deoxy-2-[[(2,2,2-trichloroethoxy)carbonyl]amino]-3,4,6-triacetyl-1-(2,2,2-trifluoro-N-phenylacetimidoyl)-α-D-glucopyranoside (**1b**)*.......... S7

*Phenyl-2-deoxy-1-thio-2-[[(2,2,2-trichloroethoxy)carbonyl]amino]-3,4,6-triacetyl-β-D-glucopyranoside (**1c**)*.......... S8

*4-Methylphenyl-2-deoxy-1-thio-2-[[(2,2,2-trichloroethoxy)carbonyl]amino]-3,4,6-triacetyl-β-D-glucopyranoside (**1d**)*.......... S8

*2-(Acetylamino)-2-deoxy-3,4,6-triacetyl-1-(2,2,2-trichloroethanimidate)-α-D-glucopyranoside (**1e**)*.......... S9

*Phenyl-2-(acetylamino)-2-deoxy-1-thio-3,4,6-triacetyl-β-D-glucopyranoside (**1f**)*.......... S11

*2-(Acetylamino)-2-deoxy-3,4,6-triacetyl-1-(2,2,2-trifluoro-N-phenylacetimidoyl)-β-D-glucopyranoside (**1g**)*.......... S11

*Alanine-2-(phenylsulfonyl)ethyl ester (**S7**)*.......... S13

*N-Acetyl-1-O-(phenylmethyl)-4,6-O-(phenylmethylene)-α-D-muramic acid (**S10**)*.......... S14

*Phenylmethyl-2-(acetylamino)-2-deoxy-3-O-[(1R)-1-methyl-2-[[(1S)-1-methyl-2-oxo-2-[2-(phenylsulfonyl)ethoxy]ethyl]amino]-2-oxoethyl]-4,6-O-[(R)-phenylmethylene]-α-D-glucopyranoside (**S11**)*.......... S15

*Phenylmethyl-2-(acetylamino)-2-deoxy-3-O-[(1R)-1-methyl-2-[[(1S)-1-methyl-2-oxo-2-[2-(phenylsulfonyl)ethoxy]ethyl]amino]-2-oxoethyl]-6-O-(phenylmethyl)-α-D-glucopyranoside (**2a**)*.......... S16

*Phenylmethyl-2-(acetylamino)-2-deoxy-3-O-[(1R)-1-methyl-2-[[(1S)-1-methyl-2-oxo-2-[2-(phenylsulfonyl)ethoxy]ethyl]amino]-2-oxoethyl]-6-O-(acetyl)-α-D-glucopyranoside (**2b**)*.......... S17

*Phenylmethyl-2-(acetylamino)-2-deoxy-3-O-[(1R)-1-methyl-2-[[(1S)-1-methyl-2-oxo-2-[2-(phenylsulfonyl)ethoxy]ethyl]amino]-2-oxoethyl]-6-O-(phenylmethyl)-4-O-[3,4,6-tri-O-acetyl-2-deoxy-2-[[(2,2,2-trichloroethoxy)carbonyl]amino]-β-D-glucopyranosyl]-α-D-glucopyranoside (**3a**)*.......... S19

*Phenylmethyl 2-(acetylamino)-2-deoxy-3-O-[(1R)-1-methyl-2-[[(1S)-1-methyl-2-oxo-2-[2-(phenylsulfonyl)ethoxy]ethyl]amino]-2-oxoethyl]-4-O-[3,4,6-tri-O-acetyl-2-(acetylamino)-2-deoxy-β-D-glucopyranosyl]-6-O-acetyl-α-D-glucopyranoside (**4**)*.......... S21

*N-[N-Acetyl-6-O-acetyl-1-hydroxy-4-O-[3,4,6-tri-O-acetyl-2-(acetylamino)-2-deoxy-β-D-glucopyranosyl]-α-muramoyl]-l-alanine-2-(phenylsulfonyl)ethyl ester (**5**)*.......... S22

*N-[N-Acetyl-6-O-acetyl-1-O-[bis(phenylmethoxy)phosphinyl]-4-O-[3,4,6-tri-O-acetyl-2-(acetylamino)-2-deoxy-β-D-glucopyranosyl]-α-muramoyl]-l-alanine-2-(phenylsulfonyl)ethyl ester (**6**)*.......... S23

*Boc-D-Ala-D-Ala-OMe (**S14**)*.......... S25

*Boc-Lys-D-Ala-D-Ala-OMe (**S15**)*.......... S25

*H-γ-D-Glu(α-OMe)-Lys(TFA)-D-Ala-D-Ala-OMe trifluoroacetate salt (**S16**)*.......... S26

*N-[N-Acetyl-6-O-acetyl-1-O-[bis(phenylmethoxy)phosphinyl]-4-O-[3,4,6-tri-O-acetyl-2-(acetylamino)-2-deoxy-β-D-glucopyranosyl]-α-muramoyl]-alanyl-D-γ-glutamyl-N6-(2,2,2-trifluoroacetyl)-lysyl-D-alanyl-D-alanyl-2,5-dimethyl ester (**7**)* S28
*Undecaprenol (**S17**)* S29
*Undecaprenyl phosphate bisammonium salt (Und-P, **S18**)* S30
*Farnesyl phosphate (**S20**)* S31
*Geranylgeranyl phosphate (**S22**)* S32
*Solanesyl phosphate (**S24**)* S32
*Lipid II diammonium salt (**11**)* S33
*Farnesyl lipid II analogue (**8**)* S35
*Geranylgeranyl lipid II analogue (**9**)* S35
Solanesyl lipid II analogue (10) S35
References S36
Spectroscopic data of synthesized compounds S37

General experimental information

Solvents and reagents

All experiments were conducted using flame-dried glassware under an inert atmosphere unless otherwise indicated. To safeguard against undesired reactions of air and moisture-sensitive reagents, they were introduced while maintaining the inert environment. Dry solvents were prepared by adding 20 wt %/v freshly activated 3 Å molecular sieves (MS) from HPLC grade solvents within an inert atmosphere, allowing them to stand for 24–48 hours. All remaining solvents and reagents were sourced directly from commercial suppliers.

Chromatography

Reactions were monitored through the use of thin-layer chromatography (TLC). We employed Merck Kieselgel 60 F254 silica plates with a particle size of 230–400 mesh for TLC. Visualization was achieved through staining with either aqueous potassium permanganate or phosphomolybdic acid solutions, or by employing UV light at wavelengths of 254 or 354 nm.

Spectroscopy

The ^{1}H and ^{13}C Nuclear Magnetic Resonance (NMR) spectra were recorded at 400 MHz using a Bruker spectrometer with TopSpinTM software. The spectra are quoted in parts per million (ppm) relative to tetramethylsilane, and the samples were prepared by dissolving the material in $CDCl_3$, CD_3OD, or DMSO-d_6. The ^{1}H, ^{13}C, and ^{31}P NMR spectra were acquired at 298 K and were processed and viewed using MestreNova. The chemical shifts (δ) are given in ppm and coupling constants (*J*) are given in hertz (Hz). The multiplicity is abbreviated as app = apparent, br = broad, s = singlet, d = doublet, t = triplet, q = quartet, and m = multiplet. The ^{13}C NMR spectra are reported in δ/ppm. High-resolution mass spectra

(HRMS) were recorded on a ToF mass spectrometer using the electrospray ionization (ESI) technique.

Purification of compounds using high-performance liquid chromatography (HPLC)

After the final step, all lipid II analogues were purified by reversed-phase high-performance column chromatography (RP-HPLC). Purification was performed on a Perkin Elmer HPLC system composed of a 200 series binary pump, UV–vis detector, vacuum degasser, Rheodyne 7725i injector equipped with a 2 mL sample loop, and Phenomenex Luna C18 column (5 µm, 250 × 21.2 mm). The system was operated using ThermoFisher Chromeleon 7.2 software. Runs were performed at a flow rate of 10 mL/min with UV detection at 220 nm. Solvent A = 50 mM NH_4HCO_3 (aq), solvent B = MeOH. Gradient = 2 to 98% B over 30 min, 98% B for 10 min, 98 to 2% B over 1 min and 2% B for 4 min. Product-containing fractions were pooled, concentrated under vacuum to remove MeOH, frozen, and then lyophilized to yield pure lipid II analogues as white flocculent solids.

Experimental procedures and characterization of products

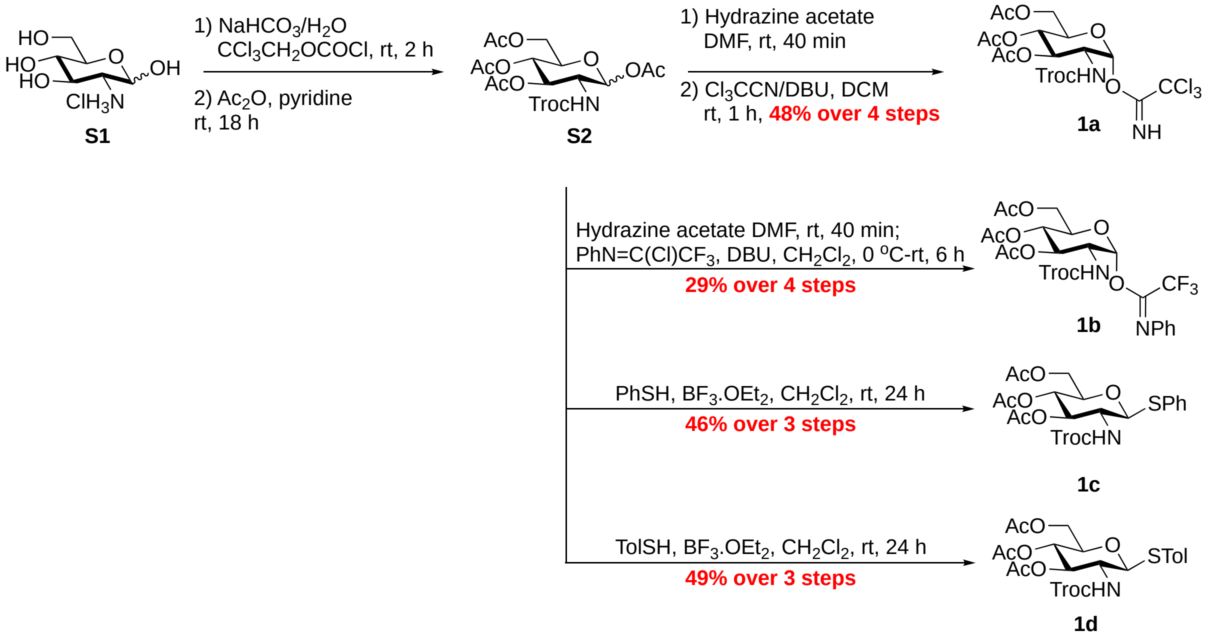

Scheme S1: Synthesis of glycosyl donors **1a–d**.

*2-Deoxy-2-[[(2,2,2-trichloroethoxy)carbonyl]amino]-3,4,6-triacetyl-1-(2,2,2-trichloroacetimidoyl)-α-D-glucopyranoside (**1a**)*

Compound **1a** was synthesized according to a previously reported procedure [1], with modifications. Initially, D-glucosamine hydrochloride (**S1**, 20.0 g, 92.75 mmol) and sodium bicarbonate (15.6 g, 185.6 mmol) were dissolved in 240 mL of water and stirred vigorously for 5 minutes. Subsequently, 2,2,2-trichloroethoxycarbonyl chloride (15.3 mL, 111.2 mmol) was added drop by drop, and the solution was stirred at room temperature for 2 hours, leading to the formation of a white precipitate. The suspension was filtered, washed with water, and co-evaporated with toluene (3 × 50 mL).

The resulting white powder was then dissolved in dry pyridine (150 mL) and acetic anhydride (75 mL) and stirred at room temperature for 18 hours. The reaction mixture was concentrated under vacuum and co-evaporated with toluene (3 × 50 mL). The oily residue obtained was dissolved in $CHCl_3$ (200 mL) and washed with 1 M HCl (100 mL). The aqueous

phase was back-extracted with $CHCl_3$ (200 mL), and the combined organic extracts were washed with brine (100 mL). The organic phase was dried over anhydrous sodium sulfate and concentrated under vacuum, resulting in the formation of 1,3,4,6-tetra-*O*-acetyl-2-troc-D-glucosamine **S2** as a white solid (26.2 g, 50.22 mmol).

Subsequently, compound **S2** was dissolved in dry DMF (200 mL), and hydrazine acetate (5.56 g, 60.26 mmol) was added. The reaction mixture was stirred at room temperature for 40 minutes, then diluted with EtOAc (300 mL) and washed with water (300 mL), saturated sodium bicarbonate (200 mL), and water (200 mL). The combined aqueous phases were back-extracted with EtOAc (300 mL), and the combined organic extracts were washed with brine (200 mL), dried over anhydrous sodium sulfate, and concentrated under vacuum.

The resulting white solid was dissolved in CH_2Cl_2 (200 mL), and trichloroacetonitrile (50.3 mL, 502.2 mmol) was added. To this mixture, 1,8-diazabicycloundec-7-ene (1.5 mL, 10 mmol) was introduced, and the reaction mixture was stirred at room temperature for 90 minutes. The crude reaction mixture was then purified via flash column chromatography (SiO_2, using a 2:1 hexane/EtOAc mixture with 0.1% triethylamine) to obtain product **1a** as a light-yellow foam (48% yield over 4 steps). The spectroscopic data were in agreement with those previously reported.[1] ^{1}H-NMR (400 MHz, $CDCl_3$) δ 8.81 (s, 1H, acetimidate-NH), 6.43 (d, *J* = 3.6 Hz, 1H, H1), 5.36 (dd, *J* = 10.8, 9.5 Hz, H3), 5.29–5.19 (m, 2H, H4, Troc-NH), 4.76–4.68 (m, 2H, Troc-CH_2), 4.32–4.25 (m, 2H, H2 + H6), 4.17–4.09 (m, 2H, H5 + H6), 2.08 (s, 3H, 1 x Ac), 2.06 (s, 6H, 2 x Ac); ^{13}C-NMR (100 MHz, $CDCl_3$) δ 171.1, 170.5, 169.3, 160.4, 154.1, 95.2, 94.5, 90.7, 74.7, 70.3, 67.4, 61.4, 53.9, 20.7, 20.6. HRMS (ESI) Calcd for $C_{17}H_{20}N_2Cl_6O_{10}Na$ $[M+Na]^+$ 644.9147, found 644.9172.

*2-Deoxy-2-[[(2,2,2-trichloroethoxy)carbonyl]amino]-3,4,6-triacetyl-1-(2,2,2-trifluoro-N-phenylacetimidoyl)-α-D-glucopyranoside (**1b**)*

Compound **1b** was synthesized according to a previously reported procedure[2], with modifications. Dissolve Troc-protected 1,3,4,6-tetra-*O*-acetyl-D-glucosamine **S2** (1 g, 2.08 mmol) in 20 mL of dry DMF, and add hydrazine acetate (212 mg, 2.08 mmol). Stir the reaction mixture at room temperature for 40 minutes. Next, dilute the mixture with 30 mL of EtOAc and wash it successively with 30 mL of water, 20 mL of saturated sodium bicarbonate, and 20 mL of water. The combined aqueous phases were back-extracted with 30 mL of EtOAc, and the combined organic extracts were washed with 20 mL of brine, dried using anhydrous sodium sulfate, and concentrated under vacuum. The resulting white solid was then dissolved in 50 mL of dry CH_2Cl_2, and (*N*-phenyl)-2,2,2-trifluoroacetimidoyl chloride (0.90 mL, 6.24 mmol) was added to the mixture. The reaction mixture was cooled to 0 °C, and then 1,8-diazabicycloundec-7-ene (0.31 mL, 2.08 mmol) was added to it. Stir the reaction mixture at room temperature for 6 hours, and concentrate it under vacuum. The crude residue was purified by flash column chromatography using silica gel (SiO_2) with hexane/EtOAc 3:1 mixture to yield product **1b** as a white solid (361 mg, 29% yield over 4 steps). The spectroscopic data were in agreement with those previously reported.[2] ^{1}H-NMR (400 MHz, $CDCl_3$) δ 7.35–7.28 (m, 2H, Ar-H), 7.17–7.10 (m, 1H, Ar-H), 6.82 (d, J = 7.7 Hz, 2H, Ar-H), 5.40–5.23 (m, 2H, H1 + H3), 5.14 (t, J = 9.5 Hz, 1H, H4), 4.81–4.68 (m, 2H, Troc-CH_2), 4.29 (dd, J = 12.7, 4.6 Hz, 1H, H6), 4.20–4.12 (m, 1H, H6'), 2.09 (s, 3H, Ac), 2.06 (s, 3H, AC), 2.03 (s, 3H, Ac). ^{13}C-NMR (100 MHz, $CDCl_3$) δ 170.6, 169.3, 142.9, 128.9, 124.7, 119.2, 94.6, 76.7, 74.6, 72.9, 71.6, 68.0, 61.6, 55.3, 20.7, 20.6, 20.5. HRMS (ESI) Calcd for $C_{23}H_{25}N_2Cl_3F_3O_{10}$ $[M+H]^+$ 651.0521, found 651.0436.

*Phenyl-2-deoxy-1-thio-2-[[(2,2,2-trichloroethoxy)carbonyl]amino]-3,4,6-triacetyl-β-D-glucopyranoside (**1c**)*

Compound **1c** was synthesized according to a previously reported procedure[3], with modifications. Compound **S2** (3.0 g, 5.75 mmol) was dissolved in 20 mL of anhydrous CH_2Cl_2. Then, thiophenol (0.88 mL, 8.62 mmol) and $BF_3{\cdot}OEt_2$ (1.45 mL, 11.3 mmol) were added to the solution. Stir the reaction mixture at room temperature for 24 hours. Afterward, dilute the reaction mixture with 20 mL of CH_2Cl_2, then wash it with saturated $NaHCO_3$ solution (2 × 20 mL), and dry it over $MgSO_4$. Filter the mixture and concentrate it under vacuum. The resulting crude residue was purified through flash column chromatography on silica gel, using a gradient of 8:2 to 7:3 hexane/EtOAc, yielding product **1c** as a white solid (1.51 g, 46% over 4 steps). The spectroscopic data were in agreement with those previously reported.[3] ^{1}H-NMR (400 MHz, $CDCl_3$) δ 7.56–7.48 (m, 2H, Ar-H), 7.31 (dd, *J* = 4.9, 1.9 Hz, 3H, Ar-H), 5.29 (t, *J* = 9.7 Hz, 1H, H1), 5.03 (t, *J* = 9.7 Hz, 1H, H3), 4.87 (d, *J* = 10.3 Hz, 1H, H4), 4.84–4.69 (m, 2H, Troc-CH_2), 4.20 (qd, *J* = 12.3, 3.9 Hz, 2H, H2 + H6), 3.78 – 3.64 (m, 2H, H5 + H6’), 2.08 (s, 3H, 1 x Ac), 2.01 (s, 6H, 2 x Ac). ^{13}C-NMR (100 MHz, $CDCl_3$) δ 170.5, 169.4, 153.9, 133.0, 132.0, 129.0, 128.4, 95.4, 86.6, 75.9, 74.6, 73.2, 68.6, 62.3, 60.4, 55.2, 21.0, 20.7, 20.6, 20.5, 14.2. HRMS (ESI) Calcd for $C_{21}H_{24}Cl_3NO_9SNa$ $[M+Na]^+$ 594.0135, found 594.0123.

*4-Methylphenyl-2-deoxy-1-thio-2-[[(2,2,2-trichloroethoxy)carbonyl]amino]-3,4,6-triacetyl-β-D-glucopyranoside (**1d**)*

Compound **1d** was synthesized according to a previously reported procedure[4], with modifications. Compound **S2** (3 g, 5.75 mmol) was dissolved in anhydrous CH_2Cl_2 (20 mL). *p*-Toluenethiol (1.07 g, 8.62 mmol) and $BF_3{\cdot}OEt_2$ (1.3 mL) were added, and the reaction mixture was stirred at room temperature for 24 h. Then, the reaction mixture was diluted with CH_2Cl_2 (20 mL), washed with saturated aqueous $NaHCO_3$ (2 × 20 mL), dried over $MgSO_4$,

filtered, and concentrated under vacuum. The crude residue was purified by flash column chromatography on silica gel using a gradient of 8:2 to 7:3 hexane/EtOAc, yielding thioglycoside **1d** as a white solid (1.65 g, 49% yield over 4 steps). The spectroscopic data were in agreement with those previously reported.[4] ^{1}H-NMR (400 MHz, $CDCl_3$) δ 7.45–7.38 (m, 2H, Ar-H), 7.12 (d, *J* = 7.9 Hz, 2H, Ar-H), 5.26 (td, *J* = 13.2, 11.4, 8.0 Hz, 2H, H1 + H3), 5.01 (t, *J* = 9.7 Hz, 1H, H4), 4.86 – 4.76 (m, 2H, Troc-CH*2*), 4.73 (d, *J* = 12.0 Hz, 1H, NH), 4.20 (qd, *J* = 12.3, 3.9 Hz, 2H, H6 + H6'), 3.74–3.59 (m, 2H, H5 + H2), 2.35 (s, 3H Tol-CH_3), 2.08 (s, 3H 1 x Ac), 2.00 (s, 6H 2 x Ac). ^{13}C-NMR (100 MHz, $CDCl_3$) δ 170.6, 170.5, 169.4, 153.8, 138.7, 133.7, 129.7, 127.9, 95.4, 86.7, 75.8, 74.6, 73.2, 68.6, 62.3, 55.1, 21.2, 20.7, 20.6, 20.5. HRMS (ESI) Calcd for $C_{22}H_{26}Cl_3NO_9SNa$ $[M+Na]^+$ 608.0286, found: 608.0312.

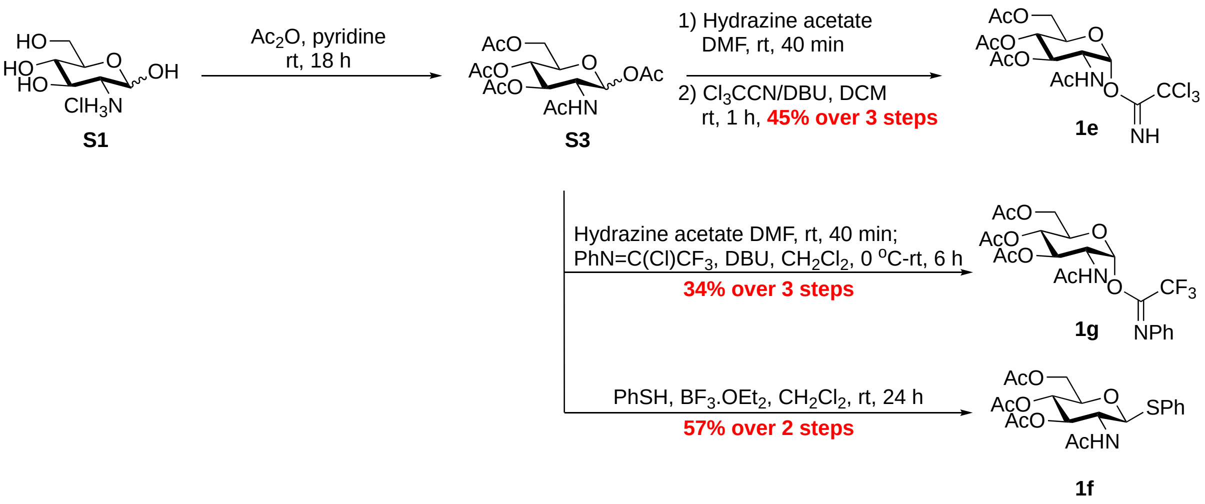

Scheme S2: Synthesis of glycosyl donors **1e–g**.

2-(Acetylamino)-2-deoxy-3,4,6-triacetyl-1-(2,2,2-trichloroethanimidate)-α-D-glucopyranoside (1e)

Compound **1e** was synthesized according to a previously reported procedure[5], with modifications. D-Glucosamine hydrochloride (**S1**, 20 g, 92.75 mmol) was dissolved in 150 mL

of dry pyridine and 75 mL of acetic anhydride, and the mixture was stirred at room temperature for 18 hours. The resulting reaction mixture was concentrated under vacuum and co-evaporated with toluene (3 × 50 mL). The oily residue obtained was dissolved in 200 mL of $CHCl_3$ and washed with 100 mL of 1 M HCl. The aqueous phase was back-extracted with 200 mL of $CHCl_3$, and the combined organic extracts were washed with 100 mL of brine. After drying the organic phase with anhydrous sodium sulfate, it was concentrated under vacuum to yield 1,3,4,6-tetra-*O*-acetyl-2-acetyl-ᴅ-glucosamine (**S3**) as a white solid.

Compound **S3** (6 g, 15.41 mmol) was then dissolved in 40 mL of dry DMF, and hydrazine acetate (1.7 g, 18.4 mmol) was added to it. The reaction mixture was stirred at room temperature for 40 minutes, diluted with 100 mL of EtOAc, and washed with 100 mL of water, saturated sodium bicarbonate, and water. The combined aqueous phases were back-extracted with 100 mL of EtOAc, and the combined organic extracts were washed with 100 mL of brine. After drying with anhydrous sodium sulfate, the organic phase was concentrated under vacuum.

The resulting white solid was dissolved in 100 mL of CH_2Cl_2, and trichloroacetonitrile (15.5 mL, 154.1 mmol) was added to it. Then, 1,8-diazabicycloundec-7-ene (0.46 mL, 3.08 mmol) was added, and the reaction mixture was stirred at room temperature for 90 minutes and concentrated under vacuum. The crude reaction mixture was purified via flash column chromatography on silica gel, using a 2:1 hexane/EtOAc mixture with 0.1% triethylamine, to obtain product **1e** as a light-yellow foam (3.4 g, 45% yield in 3 steps). The spectroscopic data were in agreement with those previously reported.[5] ^{1}H-NMR (400 MHz, $CDCl_3$) δ 8.80 (s, 1H, acetimidate NH), 6.37 (d, *J* = 3.6 Hz, 1H, H1), 5.67 (d, *J* = 8.9 Hz, 1H, H3), 5.37–5.21 (m, 2H, H4 + NH), 4.56 (ddd, *J* = 10.5, 8.9, 3.7 Hz, 1H, H2), 4.25 (dd, *J* = 12.9, 4.7 Hz, 1H, H6), 4.12 (dq, *J* = 11.9, 2.5 Hz, 2H, H6’ + H5), 2.16–2.02 (m, 9H, 3 x Ac), 1.94 (s, 3H, Ac). ^{13}C-NMR (100 MHz, $CDCl_3$) δ 171.6, 170.6, 170.0, 169.2, 160.3, 94.8, 75.6, 75.3, 75.0, 74.3, 74.0,

73.6, 70.7, 70.3, 67.3, 61.5, 51.8, 23.1, 20.8, 20.7, 20.6. HRMS (ESI) Calcd for $C_{16}H_{21}N_2Cl_3O_9Na$ $[M+Na]^+$ 513.0205, found 513.0218.

*Phenyl-2-(acetylamino)-2-deoxy-1-thio-3,4,6-triacetyl-β-D-glucopyranoside (**1f**)*

Compound **1f** was synthesized according to a previously reported procedure[6], with modifications. Compound **S3** (2 g, 5.14 mmol) was dissolved in 30 mL of anhydrous CH_2Cl_2. Then, thiophenol (0.80 mL, 7.71 mmol) and 1.3 mL of $BF_3{\cdot}OEt_2$ were added to it. Stir the reaction mixture at room temperature for 24 h. Following this, dilute the reaction mixture with 50 mL of CH_2Cl_2, then wash with saturated aqueous $NaHCO_3$ (2 x 50 mL), dry over $MgSO_4$, filtered, and concentrate under vacuum. Purify the compound by column chromatography using a gradient of 8:2 to 7:3 hexane/EtOAc, to yield thioglycoside **1f** as a white solid (1.28 g, 57% in 2 steps). The spectroscopic data were in agreement with those previously reported.[6] ^{1}H-NMR (400 MHz, $CDCl_3$) δ 7.53–7.47 (m, 2H, Ar-H), 7.33–7.27 (m, 3H, Ar-H), 5.59 (d, *J* = 9.2 Hz, 1H), 5.22 (dd, *J* = 10.2, 9.4 Hz, 1H), 5.06 (dd, *J* = 10.0, 9.3 Hz, 1H), 4.85 (d, *J* = 10.4 Hz, 1H), 4.22 (dd, *J* = 12.2, 5.3 Hz, 1H), 4.17 (dd, *J* = 12.2, 2.6 Hz, 1H), 4.03 (td, *J* = 10.3, 9.1 Hz, 1H), 3.72 (ddd, *J* = 10.0, 5.3, 2.6 Hz, 1H), 2.08 (s, 3H, Ac), 2.02 (d, *J* = 2.6 Hz, 6H, 2 x Ac). ^{13}C-NMR (100 MHz, $CDCl_3$) δ 171.0, 170.6, 170.0, 169.3, 132.5, 128.9, 128.1, 86.7, 75.9, 73.7, 68.4, 62.4, 53.5, 23.3, 20.8, 20.7, 20.6. HRMS (ESI) Calcd for $C_{20}H_{26}NO_8S$ $[M+H]^+$ 440.1374, found 440.1396.

*2-(Acetylamino)-2-deoxy-3,4,6-triacetyl-1-(2,2,2-trifluoro-N-phenylacetimidoyl)-β-D-glucopyranoside (**1g**)*

Compound **1g** was synthesized according to a previously reported procedure[7], with modifications. Compound **S3** (1 g, 2.57 mmol) was dissolved in dry DMF (10 mL), along with hydrazine acetate (284 mg, 3.08 mmol). The reaction mixture was stirred at room temperature

for 40 minutes, then diluted with EtOAc (20 mL) and washed with water (20 mL), saturated sodium bicarbonate (10 mL), and water (20 mL). The combined aqueous phases were subsequently back-extracted with EtOAc (20 mL), and the combined organic extracts were washed with brine (20 mL), dried over anhydrous sodium sulfate, and concentrated under vacuum.

The resulting white solid was dissolved in dry CH_2Cl_2 (20 mL), and (*N*-phenyl)-2,2,2-trifluoroacetimidoyl chloride (1.22 mL, 7.71 mmol) was added after cooling the reaction mixture to 0 °C. Then, 1,8-diazabicycloundec-7-ene (0.31 ml, 2.08 mmol) was added, and the reaction mixture was stirred at room temperature for 12 hours, then concentrated under vacuum. The crude residue was purified by chromatography on silica gel, using a gradient of 8:2 to 3:2 hexane/EtOAc, resulting in the isolation of product **1g** as a light-yellow solid (453 mg, 34% in 3 steps). The spectroscopic data were in agreement with those previously reported.[7] ^{1}H-NMR (400 MHz, $CDCl_3$) δ 7.36–7.28 (m, 2H, Ar-H), 7.18–7.10 (m, 1H, Ar-H), 6.81 (d, *J* = 7.8 Hz, 2H, Ar-H), 6.26 (s, 1H, NH), 5.74 (d, *J* = 8.8 Hz, 1H, H1 + H3), 5.34–5.17 (m, 2H, H4 + H2), 4.50 (t, *J* = 9.8 Hz, 1H), 4.25 (dd, *J* = 12.5, 4.5 Hz, 1H, H6), 4.10 (dd, *J* = 12.4, 2.3 Hz, 1H, H6’), 4.01 (d, *J* = 9.9 Hz, 1H, H5), 2.09 (s, 3H, AC), 2.06 (s, 6H, 2 x Ac), 1.97 (s, 3H, Ac). ^{13}C-NMR (100 MHz, $CDCl_3$) δ 171.6, 170.5, 170.1, 169.1, 142.8, 128.9, 124.9, 119.3, 70.5, 70.0, 67.3, 61.5, 51.6, 49.8, 37.0, 30.3, 29.9, 29.7, 28.5, 23.4, 23.0, 20.7, 20.6, 20.5. HRMS (ESI) Calcd for $C_{22}H_{25}F_3N_2O_9Na$ $[M+Na]^+$ 541.1404, found 541.1398.

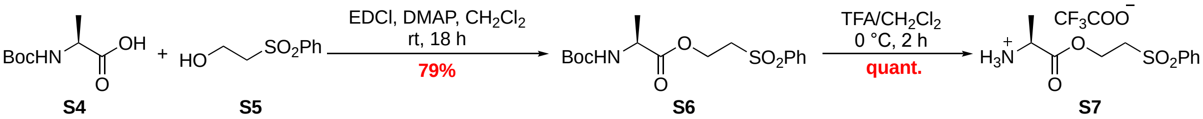

Scheme S3: Synthesis of alanine ester **S7**. Compound **S7** was synthesized using our previously established synthetic method.[1]

*Alanine-2-(phenylsulfonyl)ethyl ester (**S7**)*

In an inert atmosphere, Boc-alanine (**S4**, 4.0 g, 21.1 mmol), 2-phenylsulfonylethanol (**S5**, 3.93 g, 21.1 mmol), 1-(3-dimethylaminopropyl)-3-ethylcarbodiimide hydrochloride (4.1 g, 21.1 mmol), and 4-dimethylaminopyridine (258 mg, 0.21 mmol) were dissolved in 120 mL of dry CH_2Cl_2 and stirred at room temperature for 18 hours. The reaction mixture was subsequently treated with 1 M HCl (80 mL) and saturated $NaHCO_3$ (80 mL). Each aqueous phase was further extracted with CH_2Cl_2 (50 mL), and the combined organic extracts were washed with brine (100 mL). The organic extracts were then dried using anhydrous Na_2SO_4 and concentrated under reduced pressure. The resulting impure product underwent purification through silica gel column chromatography (3:7 EtOAc/hexane), resulting in the isolation of Boc-alanine ester **S6** as a white solid. (5.97 g, 79%). ^{1}H-NMR ($CDCl_3$, 400 MHz) δ 7.95 – 7.89 (m, 2H, *o*-ArH), 7.72 – 7.65 (m, 1H, *p*-ArH), 7.63 – 7.55 (m, 2H, *m*-ArH), 4.87 (d, *J* = 2.8, BocNH), 4.46 (t, 2H, *J* = 5.8 Hz, O-CH_2), 4.05 (q, 1H, *J* = 6.7, 6.1 Hz, Ha); 3.46 (td, *J* = 6.1, 2.3, 2H, S-CH_2), 1.42 (s, 9H, Boc), 1.22 (d, *J* = 7.2 Hz, 3H, Hβ); ^{13}C-NMR ($CDCl_3$, 100 MHz) δ 171.7, 154.0, 138.2, 133.1, 128.5, 127.1, 79.0, 57.2, 54.0, 48.0, 27.3, 17.2. Compound **S6** was dissolved in 30 mL of CH_2Cl_2 and cooled to 0 °C. Trifluoroacetic acid (30 mL) was gradually added, and the solution was allowed to warm to room temperature, where it was stirred for 2 h. The resulting solution, appearing in an orange hue, was then concentrated under reduced pressure, employing azeotropes with toluene (3 × 10 mL) to yield product **S7** as a colourless oil in a quantitative yield.

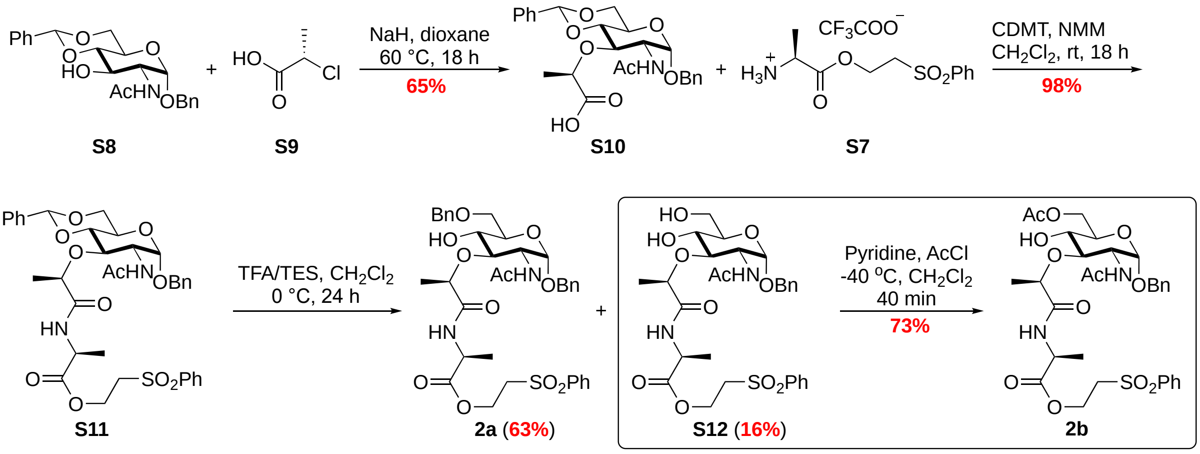

Scheme S4: Synthesis of glycosyl acceptors **2a** and **2b**. Compound **2a** was synthesized using our previously established synthetic method.[1]

*N-Acetyl-1-O-(phenylmethyl)-4,6-O-(phenylmethylene)-α-D-muramic acid (**S10**)*

Glycol **S8** (10.0 g, 25.03 mmol) was suspended in 400 mL of dry dioxane under an argon atmosphere and stirred at 60 °C. A 60% dispersion of NaH in mineral oil (2.0 g, 50.06 mmol) was added portion-wise, and the mixture was stirred for 5 minutes. Subsequently, *S*-2-chloropropionic acid (**S9**, 4.08 g, 37.55 mmol) was added, and the mixture was stirred at 60 °C for an additional 10 minutes. Another portion of a 60% dispersion of NaH in mineral oil (5.0 g, 125.15 mmol) was added, and the mixture was stirred at 60 °C for 16 hours. The reaction mixture was then cooled to room temperature, and 100 mL of water was slowly added with stirring. The volume of the reaction mixture was reduced to ≈ 100 mL using a rotary evaporator. The resulting yellow solution was subjected to extraction with 150 mL of $CHCl_3$, cooled on ice water, acidified to a pH of 1 with 6 M HCl, and then extracted with $CHCl_3$ (3 × 150 mL). The combined organic extracts were washed with 200 mL of H_2O, dried over anhydrous Na_2SO_4, and concentrated under reduced pressure. The resulting yellow solid was washed with a mixture of hexane and diethyl ether (1:1, 3 × 50 mL) to obtain product **S10** as an off-white solid (7.68 g, 65%). ^{1}H-NMR (DMSO-d_6, 400 MHz) δ 12.92 (br s, 1H, COOH), 8.09 (br s, 1H,

NHAc), 7.50-7.24 (m, 10H, Ar-H), 5.71 (s, 1H, PhCH), 5.05 (d, 1H, *J* = 3.4 Hz, H1), 4.70 (d, 1H, *J* = 12.4 Hz, PhCHH), 4.50 (d, 1H, *J* = 12.4 Hz, PhCHH), 4.28 (q, 1H, *J* = 6.9 Hz, Ala1-Hα), 4.15 (dd, 1H, *J* = 9.7, 3.8 Hz, H6), 3.83-3.67 (m, 4H, H6' + H2 + H3 + H5), 1.85 (s, 3H, NHAc), 1.27 (d, *J* = 6.8 Hz, 3H, Ala1-Hβ). ^{13}C-NMR ($CDCl_3$, 100 MHz) δ 175.7, 169.8, 138.1, 138.0, 129.3, 128.7, 128.6, 128.1, 128.0, 126.3, 100.7, 97.3, 82.0, 75.7, 75.5, 69.4, 68.3, 63.4, 54.0, 23.1, 19.2. HRMS (ESI) Calcd for $C_{25}H_{29}NO_8Na$ $[M+Na]^+$ 494.1791, found 494.1774.

*Phenylmethyl-2-(acetylamino)-2-deoxy-3-O-[(1R)-1-methyl-2-[[(1S)-1-methyl-2-oxo-2-[2-(phenylsulfonyl)ethoxy]ethyl]amino]-2-oxoethyl]-4,6-O-[(R)-phenylmethylene]-α-D-glucopyranoside (**S11**)*

Compound **S10** (5.5 g, 11.66 mmol) was suspended in dry CH_2Cl_2 (80 mL) and cooled to 0 °C. NMM (1.28 mL, 11.66 mmol) and CDMT (2.46 g, 13.99 mmol) were added, and the resulting cloudy mixture was stirred at 0 °C for 45 minutes. A solution of amine **S7** (3.3 g, 12.82 mmol) and NMM (1.28 mL, 11.66 mmol) in CH_2Cl_2 (80 mL) was then added, and the solution was stirred at room temperature. After 6 hours, DIPEA (2.3 mL, 23.32 mmol) was added to the reaction mixture and stirred overnight. The reaction mixture was subsequently filtered, and the filtrate was washed with 1 M HCl (80 mL) and brine (100 mL), dried over anhydrous Na_2SO_4, and concentrated under reduced pressure. The resulting solid was subjected to azeotropic distillation with toluene (2 × 50 mL) and $CHCl_3$ (2 × 50 mL) to yield product **S11** as a white solid (8.1 g, 98%). ^{1}H-NMR ($CDCl_3$, 400 MHz) δ 7.96-7.88 (m, 2H, ArH), 7.70-7.63 (m, 1H, ArH), 7.63-7.53 (m, 2H, ArH), 7.49-7.43 (m, 2H, ArH), 7.41-7.27 (m, 8H, ArH), 6.94 (d, *J* = 7.1 Hz, 1H, Ala1-NH), 6.19 (d, *J* = 9.0 Hz, 1H, AcNH), 5.57 (s, 1H, O_2CH), 4.95 (d, *J* = 3.8 Hz, 1H, H1), 4.71 (d, *J* = 11.8 Hz, 1H, PhCHH), 4.56 – 4.38 (m, 3H, PhCHH + OCH_2), 4.34-4.21 (m, 2H, H2 + H6), 4.16 (t, *J* = 7.2 Hz, 1H, Ala1-Hα), 4.07 (q, *J* = 6.7 Hz, 1H, OCH), 3.91-3.85 (m, 1H, H5), 3.80-3.64 (m, 3H, H6' + H3 + H4), 3.49-3.34 (m, 2H,

SC<u>H</u>$_2$), 1.93 (s, 3H, Ac), 1.37 (d, *J* = 6.8 Hz, 3H, MurNAc-CH_3), 1.30 (d, *J* = 7.3 Hz, 3H, Ala1-Hβ); ^{13}C-NMR ($CDCl_3$, 100 MHz) δ 173.0, 171.8, 170. 5, 137.1, 136.7, 134.1, 129.5, 128.7, 128.4, 128.3, 128.2, 128.1, 125.9, 101.5, 97.6, 81.5, 78.5, 78.2, 70.2, 68.9, 63.2, 58.0, 55.0, 53.0, 48.0, 23.4, 19.4, 17.2. HRMS (ESI) Calcd for $C_{36}H_{42}N_2NaO_{11}S$ $[M+Na]^+$ 733.2402, found 733.2403.

Phenylmethyl-2-(acetylamino)-2-deoxy-3-O-[(1R)-1-methyl-2-[[(1S)-1-methyl-2-oxo-2-[2-(phenylsulfonyl)ethoxy]ethyl]amino]-2-oxoethyl]-6-O-(phenylmethyl)-α-D-glucopyranoside ***(2a)***

Benzylidene **S11** (8.00 g, 11.25 mmol) was initially dissolved in 100 mL of anhydrous CH_2Cl_2 and then cooled to 0 °C. Following this, triethylsilane (8.98 mL, 56.27 mmol) was slowly added, and the solution was stirred for 5 minutes. Over 5 minutes, 4.31 mL of trifluoroacetic acid (56.27 mmol) were added, and the reaction mixture was maintained at 0 °C for 6 h. Another portion of TFA (2.6 mL, 33.75 mmol) was added, and the reaction mixture was stirred for an additional 18 hours at 0 °C. Subsequently, the reaction mixture was diluted with 100 mL of CH_2Cl_2 and washed with 100 mL of saturated aqueous $NaHCO_3$. The aqueous phase was then back-extracted using 2 × 100 mL of CH_2Cl_2, and the combined organic extracts were washed with 100 mL of brine. The organic phase was subsequently dried over anhydrous Na_2SO_4, followed by concentration under reduced pressure. The resulting crude product was further purified using column chromatography on silica gel, employing an eluent mixture of 8:2 to 10:0 EtOAc/hexane, resulting in the isolation of glycosyl acceptor **2a** as a white solid (5.1 g, 63%) and **S12** as an orange solid (1.1 g, 16%). *Characteristic data for **2a***: ^{1}H-NMR ($CDCl_3$, 400 MHz) δ 7.94-7.86 (m, 2H, ArH), 7.70-7.64 (m, 1H, ArH), 7.61-7.53 (m, 2H, ArH), 7.39-7.28 (m, 10H, ArH), 6.92 (d, *J* = 7.3 Hz, 1H, Ala1-NH), 6.07 (d, *J* = 9.0 Hz, 1H, MurNAc-NH), 4.92 (d, *J* = 3.6 Hz, 1H, H1), 4.70 (d, *J* = 11.7 Hz, 1H, OC*H*H), 4.65-4.53 (m, 2H, OC*H*H

+ OCH*H*), 4.49-4.36 (m, 3H, OCH*H* + OCH_2), 4.27-4.18 (m, 2H, H2 + Ala1-Hα), 4.14 (q, *J* = 6.7 Hz, 1H, MurNAc-OCH), 3.81 (dt, *J* = 9.2, 4.3 Hz, 1H, H5), 3.75 (dd, *J* = 10.2, 4.5 Hz, 1H, H6), 3.72-3.66 (m, 2H, H3 + H4), 3.53 (dd, *J* = 10.4, 8.6 Hz, 1H, H6), 3.47-3.33 (m, 2H, S-CH_2), 2.98 (d, *J* = 3.1, 1H, OH), 1.90 (s, 3H, NHAc), 1.41 (d, *J* = 6.7 Hz, 3H, MurNAc-CH_3), 1.31 (d, *J* = 7.2 Hz, 3H, Ala1-Hβ); ^{13}C-NMR ($CDCl_3$, 100 MHz) δ 173.0, 171.9, 170.3, 139.1, 137.8, 137.0, 134.1, 129.5, 128.6, 128.5, 128.2, 128.1, 127.8, 127.7, 97.1, 80.5, 77.8, 73.7, 71.6, 70.4, 70.2, 69.9, 58.0, 54.9, 52.5, 47.9, 23.4, 19.0, 17.1. HRMS (ESI) Calcd for $C_{36}H_{44}N_2O_{11}SNa$ $[M+Na]^+$ 735.2558, found 735.2566. *Characteristic data for **S12***: : ^{1}H-NMR ($CDCl_3$, 400 MHz) δ 7.93-7.89 (m, 2H, Ar-H), 7.70-7.65 (m, 1H, Ar-H), 7.61-7.56 (m, 2H, Ar-H), 7.36-7.28 (m, 5H, Ar-H), 7.11 (d, *J* = 7.3 Hz, 1H, Ala-N<u>H</u>), 6.47 (d, *J* = 8.2 Hz, 1H, Ac<u>N</u>H), 4.95 (d, *J* = 3.6 Hz, 1H, H1), 4.68 (d, *J* = 11.8 Hz, 1H, H8), 4.48-4.32 (m, 3H, H8' + H15 + H15'), 4.31-4.20 (m, 2H, H6 + H6'), 4.15 (ddd, *J* = 10.4, 8.6, 3.6 Hz, 1H, H9), 3.89-3.54 (m, 6H, H3 + H4 + H5 + H2), 3.49-3.31 (m, 2H, H16 + H16'), 1.92 (s, 3H, NH<u>Ac</u>), 1.40 (d, *J* = 6.7 Hz, 3H, H11), 1.31 (d, *J* = 7.3 Hz, 3H, H10); ^{13}C-NMR ($CDCl_3$, 100 MHz) δ 173.5, 172.1, 170.7, 139.1, 137.1, 134.2, 129.5, 128.6, 128.2, 128.1, 128.0, 97.1, 79.9, 77.2, 72.0, 70.1, 69.7, 61.9, 58.1, 54.9, 52.7, 47.9, 23.3, 19.2, 17.0. HRMS (ESI) Calcd for $C_{29}H_{39}N_2O_{11}S$ $[M+H]^+$ 623.2269, found 623.2298.

*Phenylmethyl-2-(acetylamino)-2-deoxy-3-O-[(1R)-1-methyl-2-[[(1S)-1-methyl-2-oxo-2-[2-(phenylsulfonyl)ethoxy]ethyl]amino]-2-oxoethyl]-6-O-(acetyl)-α-ᴅ-glucopyranoside **(2b)***

800 mg (1.29 mmol) of diol **S12** was dissolved in 20 mL of anhydrous CH_2Cl_2 and cooled to −40 °C. Subsequently, 0.1 mL of pyridine (1.29 mmol) was added, and the solution was stirred for 5 minutes, after which 0.09 mL of acetyl chloride (1.29 mmol) was added dropwise. The reaction mixture was allowed to stir for 40 minutes at the same temperature. Following this, the reaction mixture was diluted with 20 mL of CH_2Cl_2 and washed with 20 mL

of saturated aqueous $NaHCO_3$. The aqueous phase underwent back-extraction with 2 × 10 mL of CH_2Cl_2, and the combined organic extracts were further washed with 20 mL of brine. After drying over anhydrous Na_2SO_4, the organic phase was concentrated under reduced pressure. The resulting crude product was subsequently purified through column chromatography using silica gel with an eluent mixture ranging from 3:7 to 3:2 EtOAc/hexane, yielding glycosyl acceptor **2b** as a white solid (780 mg, 73%). The spectroscopic data were in agreement with those previously reported.[8] ^{1}H-NMR ($CDCl_3$, 400 MHz) δ 7.93-7.90 (m, 2H, Ar-H), 7.71-7.67 (m, 1H, Ar-H), 7.61-7.57 (m, 2H, Ar-H), 7.38-7.30 (m, 5H, Ar-H), 6.92 (d, *J* = 7.4 Hz, 1H, Ala-NH), 6.13 (d, *J* = 9.0 Hz, 1H, AcNH), 4.93 (d, *J* = 3.7 Hz, 1H, H1), 4.69 (d, *J* = 11.7 Hz, 1H, H8), 4.54-4.39 (m, 4H, H8' + H15 + H15' + H9), 4.30-4.09 (m, 5H, H3 + H4 + H5 + H2), 3.80 (ddd, *J* = 9.6, 3.8, 2.1, 1H), 3.59-3.33 (m, 5H, + H16 + H16'), 3.15 (d, *J* = 4.0 Hz, 1H), 2.13 (s, 3H OAc), 1.92 (s, 3H, NHAc), 1.42 (d, *J* = 6.8 Hz, 3H, H13), 1.33 (d, *J* = 7.2 Hz, 3H, H10); ^{13}C-NMR ($CDCl_3$, 100 MHz) δ 172.6, 171.9, 171.8, 170.4, 139.1, 136.9, 134.2, 129.5, 128.7, 128.3, 128.2, 128.1, 97.3, 80.0, 77.9, 70.5, 70.1, 69.7, 63.0, 58.0, 54.9, 52.6, 47.9, 23.4, 20.9, 19.0, 17.1 HRMS (ESI) Calcd for $C_{31}H_{41}N_2O_{12}S$ $[M+H]^+$ 665.2375, found 665.2346.

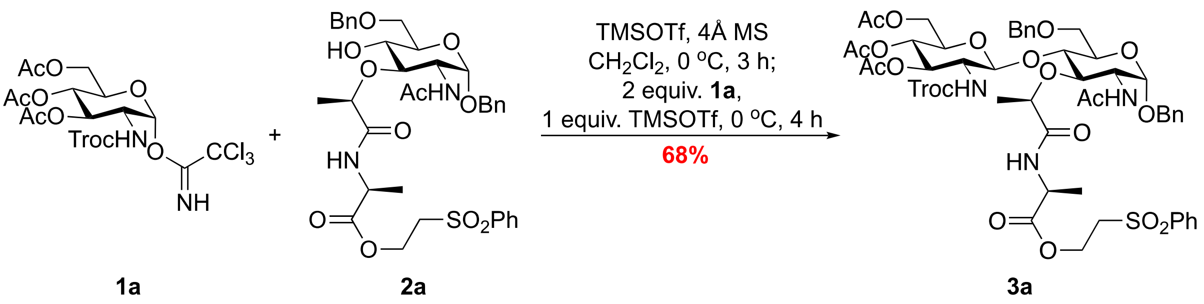

Scheme S5: Optimized glycosylation reaction **3a**.

*Phenylmethyl-2-(acetylamino)-2-deoxy-3-O-[(1R)-1-methyl-2-[[(1S)-1-methyl-2-oxo-2-[2-(phenylsulfonyl)ethoxy]ethyl]amino]-2-oxoethyl]-6-O-(phenylmethyl)-4-O-[3,4,6-tri-O-acetyl-2-deoxy-2-[[(2,2,2-trichloroethoxy)carbonyl]amino]-β-D-glucopyranosyl]-α-D-glucopyranoside (**3a**)*

Compound **3a** was synthesized according to optimized literature procedure.[1] 25 g of 4 Å molecular sieves were placed in a round-bottomed flask and heated under vacuum using a heat gun for 5 minutes before cooling to ambient temperature. The flask was then depressurized with argon and directly employed in the subsequent reaction. A solution of acceptor **1a** (3.0 g, 4.2 mmol) in dry CH_2Cl_2 (50 mL) was added into this flask under an argon atmosphere, and the suspension was gently stirred. TMSOTf (0.91 mL, 5.04 mmol) was added, followed by a solution of acetimidate donor **2a** (7.9 g, 12.6 mmol) in dry CH_2Cl_2 (50 mL). The resulting suspension was stirred at 0 °C for 3 hours. Subsequently, another portion of donor **2a** (5.25 g, 8.4 mmol) and TMSOTf (0.76 mL, 4.2 mmol) were added, and the reaction continued to be stirred for an additional 3 hours at 0 °C. Then, the reaction mixture was filtered, and the filtrate was diluted with CH_2Cl_2 (100 mL). The organic part was washed with 100 mL of saturated sodium bicarbonate and 100 mL of brine and then dried over anhydrous sodium sulfate. The crude was concentrated under vacuum and subjected to purification through column chromatography using silica gel with a gradient eluent mixture of 3:2 EtOAc/hexane to yield product **3a** in the form of a white foam (3.3 g, 68%). The spectroscopic data were in agreement with our previously reported data.[1] ^{1}H-NMR ($CDCl_3$, 400 MHz) δ 7.95-7.88 (m, 2H, ArH), 7.69-7.63 (m, 1H, ArH), 7.59-7.41 (m, 6H, ArH), 7.37-7.24 (m, 6H, ArH), 6.89 (d, *J* = 7.5 Hz, 1H, Ala1NH), 6.66 (d, *J* = 7.3 Hz, 1H, MurNAc-NH), 5.11 (d, *J* = 3.6 Hz, 1H, MurNAc-H1), 4.98 (t, *J* = 9.6 Hz, 1H, GlcNAc-H4), 4.87 (d, *J* = 11.9 Hz, 1H, MurNAc-1-CHHPh), 4.81-4.74 (m, 2H, GlcNAc-H3 + Troc-CHH), 4.64-4.55 (m, 2H, Troc-CHH + MurNAc-6-CHHPh), 4.49-4.30 (m, 4H, OCHH + MurNAc-6-CHHPh + OCHH + MurNAc-1-CHHPh), 4.27–4.06 (m,

5H, MurNAc-H2 + MurNAc-CHO + GlcNAc-H1 + GlcNAc-H6 + Ala1Hα), 3.98 (dd, *J* = 12.3, 2.2 Hz, 1H, GlcNAc-H6), 3.92 (t, *J* = 9.5 Hz, 1H, MurNAc-H3), 3.71-3.52 (m, 3H, MurNAc-H6 + MurNAc-H4 + MurNAc-H5), 3.47-3.37 (m, 4H, CH_2S + GlcNAc-H2 + GlcNAc-H5), 2.06 – 1.94 (m, 9H, 3 x Ac), 1.90 (s, 3H, Ac), 1.34 (d, *J* = 6.7 Hz, 3H, Ala1Hβ), 1.27-1.23 (m, 3H, MurNAc-CH_3). ^{13}C-NMR ($CDCl_3$, 100 MHz) δ 173.5, 171.8, 170.7, 170.4, 170.3, 169.4, 154.2, 139.2, 137.4, 137.2, 134.1, 129.4, 129.3, 129.1, 129.0, 128.5, 128.1, 128.0, 99.9, 97.1, 95.6, 77.5, 77.3, 76.6, 75.6, 74.4, 73.7, 72.1, 71.2, 70.4, 70.2, 68.3, 67.2, 61.4, 58.1, 56.2, 54.9, 53.6, 47.7, 23.2, 20.6, 20.6, 18.3, 17.4; HRMS (ESI) Calcd for $C_{51}H_{62}Cl_3N_3O_{20}SNa$ $[M+Na]^+$ 1196.2604, found 1196.2579.

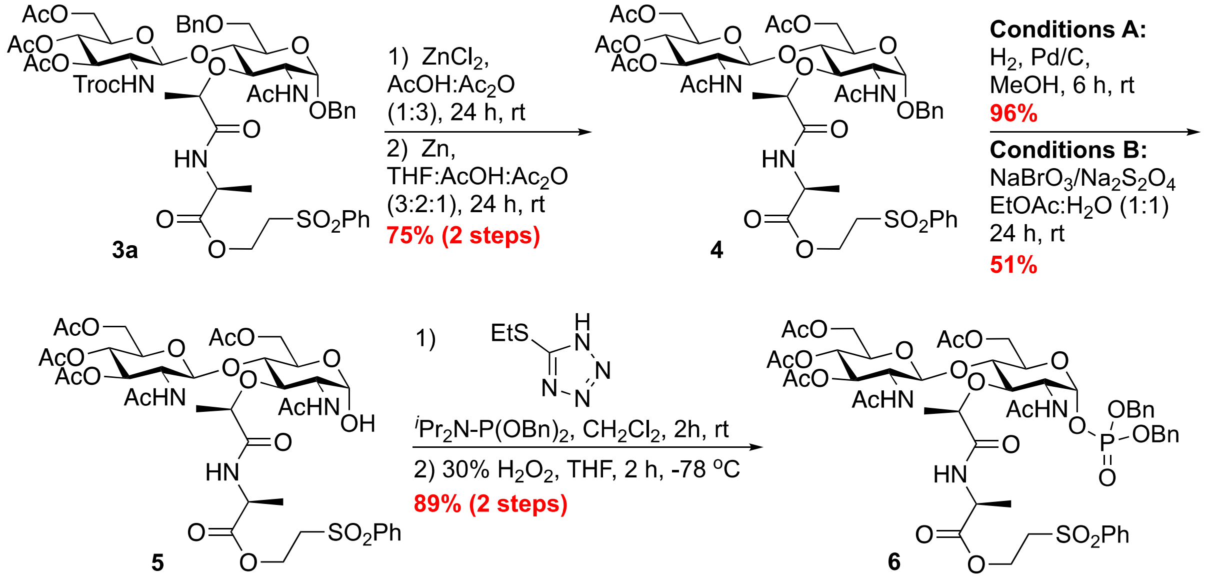

Scheme S6. Synthesis of GlcNAc-MurNAc disaccharide phosphate core **6**. Compound **6** was synthesized using our previously established synthetic method.[1]

*Phenylmethyl 2-(acetylamino)-2-deoxy-3-O-[(1R)-1-methyl-2-[[(1S)-1-methyl-2-oxo-2-[2-(phenylsulfonyl)ethoxy]ethyl]amino]-2-oxoethyl]-4-O-[3,4,6-tri-O-acetyl-2-(acetylamino)-2-deoxy-β-D-glucopyranosyl]-6-O-acetyl-α-D-glucopyranoside (**4**)*

The Troc-disaccharide **3a** (2.0 g, 1.70 mmol) was dissolved in Ac_2O (9 mL) and AcOH (4 mL), and to this solution, a solution of anhydrous $ZnCl_2$ (2.3 g, 17.0 mmol) in Ac_2O (4 mL) and AcOH (2 mL) was added. The reaction mixture was stirred for 24 hours at room temperature, after which zinc dust (4.45 g, 68.0 mmol) and a mixture of THF (18 mL), Ac_2O (10 mL), and AcOH (5 mL) were added. The reaction mixture was further stirred for 24 h at room temperature, filtered through celite, washed with 200 mL of EtOAc, and concentrated under reduced pressure. The resulting residue was co-evaporated with toluene (2 × 30 mL) and re-dissolved in EtOAc (200 mL). The organic layer was washed with saturated sodium bicarbonate (2 × 10 mL), which was then back-extracted with EtOAc (100 mL). The combined organic phases were washed with 100 mL of water and 100 mL of brine, dried over anhydrous Na_2SO_4, and concentrated under reduced pressure. The crude product underwent purification by silica gel column chromatography using an eluent mixture of EtOAc/MeOH ranging from 100:0 to 98:2, resulting in the isolation of product **4** as a white foam (1.27 grams, 75% yield). ^{1}H-NMR ($CDCl_3$, 400 MHz) δ 7.92-7.86 (m, 2H, *o*-ArH), 7.71-7.63 (m, 1H, *p*-ArH), 7.61-7.54 (m, 2H, *m*-ArH), 7.36-7.27 (m, 5H, ArH), 7.20 (d, *J* = 7.6 Hz, 1H, MurNAc-NH), 6.88 (d, *J* = 6.8 Hz, 1H, Ala1NH), 6.10 (d, *J* = 9.5 Hz, 1H, GlcNAc-NH), 5.16-5.09 (m, 2H, MurNAc-H1 + GlcNAc-H3), 4.65 (d, *J* = 12.1 Hz, 1H, MurNAc-1-C<u>H</u>HPh), 4.50 (d, *J* = 12.1 Hz, 1H, MurNAc-1-C<u>H</u>HPh), 4.45-4.24 (m, 7H, OC$\underline{H_2}$ + MurNAc-C<u>H</u>O + GlcNAc-H1 + GlcNAc-H6 + MurNAc-H6 2H), 4.16 (d, *J* = 12.0 Hz, 1H, GlcNAc-H6), 4.11-3.96 (m, 3H, GlcNAc-H2 + MurNAc-H2 + MurNAc-H3), 3.78 (d, *J* = 5.2 Hz, MurNAc-H5), 3.63-3.49 (m, 2H, GlcNAc-H5 + MurNAc-H4), 3.40-3.30 (m, 2H, C$\underline{H_2}$S), 2.15 (s, 3H), 2.03 (s, 3H), 2.02 (s, 3H), 2.01 (s, 3H), 1.96 (s, 3H), 1.94 (s, 3H), 1.37 (d, *J* = 6.6 Hz, 3H, MurNAc-CH_3), 1.29 (d, *J* = 7.2 Hz,

3H, Ala1Hβ); ^{13}C-NMR ($CDCl_3$, 400 MHz) δ 173.8, 171.9, 171.3, 170.9, 170.8, 170.6, 170.6, 169.3, 139.2, 137.3, 134.1, 129.4, 128.5, 128.1, 128.0, 127.9, 100.3, 96.9, 76.0, 75.6, 72.5, 71.9, 70.3, 69.5, 68.1, 62.3, 61.6, 58.0, 54.9, 54.6, 53.6, 47.9, 23.2, 23.2, 21.0, 20.6, 20.6, 20.6, 18.4, 17.3. HRMS (ESI) Calcd for $C_{45}H_{60}N_3O_{20}S$ $[M+H]^+$ 994.3491, found 994.3489.

*N-[N-Acetyl-6-O-acetyl-1-hydroxy-4-O-[3,4,6-tri-O-acetyl-2-(acetylamino)-2-deoxy-β-ᴅ-glucopyranosyl]-α-muramoyl]-l-alanine-2-(phenylsulfonyl)ethyl ester (**5**)*

Conditions A: The benzylated sugar **4** (1.1 g, 1.11 mmol) was dissolved in a mixture of 32 mL of THF and 8 mL of MeOH, and the solution was degassed using an argon balloon. Then, 10% palladium on charcoal (2.3 g, 2.22 mmol) was portion-wise added to the solution. The reaction mixture was stirred under a hydrogen balloon at room temperature and atmospheric pressure overnight and subsequently filtered through a thin layer of celite. The celite was washed with 2 × 50 mL of MeOH, and the filtrate was concentrated under reduced pressure. The resulting solid was washed with a mixture of ether and hexanes (1:2) and dried under reduced pressure to obtain lactol **5** as a white solid (960 mg, 96%).

Conditions B: The benzylated sugar **4** (0.11 grams, 0.11 mmol) was first dissolved in 2 mL of ethyl acetate, and then a solution of $NaBrO_3$ (17 mg, 0.9 mmol) in 1 mL of water was added. To the vigorously stirred two-phase system, an aqueous solution of $Na_2S_2O_4$ (15 mg, dissolved in 1 mL of water) was added drop-wise over 2 minutes at room temperature. Upon completion of the reaction, the reaction mixture was diluted with ethyl acetate, and the organic phase was washed with an aqueous solution of sodium thiosulfate. The crude product was subsequently purified through silica gel chromatography to obtain **5** as a white solid (51 mg, 51%). ^{1}H-NMR (DMSO-d_6, 400 MHz) δ 8.40 (d, *J* = 5.9 Hz, 1H, MurNAc-NH), 8.16-8.06 (m, 2H, Ala1NH and GlcNAc-NH), 8.03-7.94 (m, 2H, *o*-ArH), 7.88-7.79 (m, 1H, *p*-ArH), 7.78-7.68 (m, 2H, *m*-ArH), 6.81 (d, *J* = 4.6 Hz, 1H, MurNAc-OH), 5.27 (t, *J* = 9.9 Hz, 1H), 5.16 (t,

J = 3.5 Hz, 1H), 4.96 (t, J = 9.8 Hz, 1H), 4.74 (d, J = 8.2 Hz, 1H), 4.53-4.25 (m, 5H), 4.14-3.99 (m, 3H), 3.93-3.76 (m, 5H), 3.72 (t, J = 9.3 Hz, 1H), 3.60-3.53 (m, 1H), 3.49-3.42 (m, 2H), 2.12 (s, 3H), 2.03 (s, 3H), 2.00 (s, 3H), 1.97 (s, 3H), 1.84 (m, 3H), 1.83 (s, 3H), 1.32 (d, J = 6.7 Hz, 3H, MurNAc-CH_3), 1.15 (d, J = 7.2 Hz, 3H, Ala1Hβ); ^{13}C-NMR (DMSO-d_6, 100 MHz) δ 174.4, 172.0, 170.5, 170.4, 170.1, 170.0, 169.9, 169.8, 139.8, 134.5, 129.9, 128.2, 100.1, 90.2, 77.2, 76.3, 75.4, 72.9, 70.8, 68.9, 68.5, 63.0, 62.1, 58.6, 54.6, 54.5, 54.2, 47.7, 23.2, 23.1, 21.2, 20.9, 20.8, 20.8, 19.1, 17.2.

*N-[N-Acetyl-6-O-acetyl-1-O-[bis(phenylmethoxy)phosphinyl]-4-O-[3,4,6-tri-O-acetyl-2-(acetylamino)-2-deoxy-β-D-glucopyranosyl]-α-muramoyl]-l-alanine-2-(phenylsulfonyl)ethyl ester (**6**)*

Compound **5** (900 mg, 1 mmol) was dissolved in 10 mL of anhydrous CH_2Cl_2 and rapidly added *via* syringe to a vigorously stirred mixture of 5-ethylthio-1H-tetrazole (586 mg, 4.5 mmol) and dibenzyl-*N*,*N*'-diisopropylphosphoramidite (1.0 mL, 3.0 mmol) in anhydrous CH_2Cl_2 (10 mL) under an argon atmosphere at room temperature. After 2 hours, the reaction mixture was diluted with 60 mL of CH_2Cl_2 and subjected to washing with saturated sodium bicarbonate (50 mL), water (50 mL), and brine (50 mL). The organic solution was dried over anhydrous sodium sulfate and concentrated under reduced pressure to yield a colourless oil, which was then precipitated from a mixture of diethyl ether and hexanes (1:1), resulting in the formation of the phosphite as a light-yellow solid. The product was then dissolved in 20 mL of THF and cooled to −78 °C. Hydrogen peroxide (30%, 2.0 mL) was added dropwise *via* a syringe into the vigorously stirred solution. After the addition was complete, the cooling bath was removed, and the mixture was allowed to warm to room temperature for over 2 h. The reaction mixture was subsequently diluted with ice-cold saturated sodium sulfite (5 mL), followed by the addition of 50 mL of EtOAc, and stirred for 5 minutes. The organic layer was

washed with 20 mL of saturated $NaHCO_3$ and 20 mL of brine, dried over anhydrous sodium sulfate, and concentrated under reduced pressure, yielding phosphate **6** as a light-yellow solid. (1.0 g, 89%). ^{1}H-NMR (DMSO-d_6, 400 MHz) δ 8.70 (d, *J* = 4.5 Hz, 1H, NHAc), 8.43 (d, *J* = 7.0 Hz, 1H, NHAc), 8.09 (d, *J* = 9.1 Hz, 1H, NHAc), 7.90-7.83 (m, 2H, *o*-ArH), 7.79-7.71 (m, 1H, *p*-ArH), 7.68-7.60 (m, 2H, *m*-ArH), 7.43-7.30 (m, 10H, 2 x Bn-ArH), 5.82 (dd, *J* = 6.5, 3.1 Hz, 1H, MurNAc-H1), 5.24 (t, *J* = 9.9 Hz, 1H, GlcNAc-H3), 5.05-4.96 (m, 4H, 2 x CH_2Ph), 4.92 (t, *J* = 9.7 Hz, 1H, GlcNAc-H4), 4.74 (d, *J* = 8.3 Hz, 1H, GlcNAc-H1), 4.61 (d, *J* = 6.7 Hz, 1H, MurNAc-C*H*O), 4.35-4.19 (m, 3H, MurNAc-H6 + GlcNAc-H6 + OC*H*H), 4.11-3.96 (m, 4H, MurNAc-H6 + GlcNAc-H6 + OC*H*H + Ala1Hα), 3.88-3.73 (m, 4H, GlcNAc-H2 + GlcNAc-H5 + MurNAc-H3 + MurNAc-H5), 3.68-3.52 (m, 3H, MurNAc-H2 + SCH_2), 3.43 (dd, *J* = 9.0, 6.8 Hz, 1H, MurNAc-H4), 1.98 (s, 3H), 1.97 (s, 3H), 1.96 (s, 3H), 1.93 (s, 3H), 1.76 (s, 3H), 1.70 (s, 3H), 1.30 (d, *J* = 6.7 Hz, 3H, MurNAc-CH_3), 1.11 (d, *J* = 7.4 Hz, 3H, AlaHβ); ^{13}C-NMR (DMSO-d_6, 100 MHz) δ 175.0, 171.9, 170.4, 170.3, 170.1, 169.8, 139.8, 136.3, 134.4, 129.9, 129.0, 128.9, 128.9, 128.8, 128.3, 128.2, 128.2, 128.1, 100.1, 76.5, 76.3, 74.4, 72.9, 71.2, 70.9, 69.2, 69.2, 69.0, 68.9, 68.8, 62.1, 58.4, 54.6, 47.9, 23.1, 22.9, 22.5, 21.0, 20.9, 20.8, 19.5, 17.0; ^{31}P-NMR (DMSO-d_6, 162 MHz) δ -2.76; HRMS (ESI) Calcd for $C_{52}H_{66}N_3NaO_{23}PS$ $[M+Na]^+$ 1186.3443, found 1186.3456.

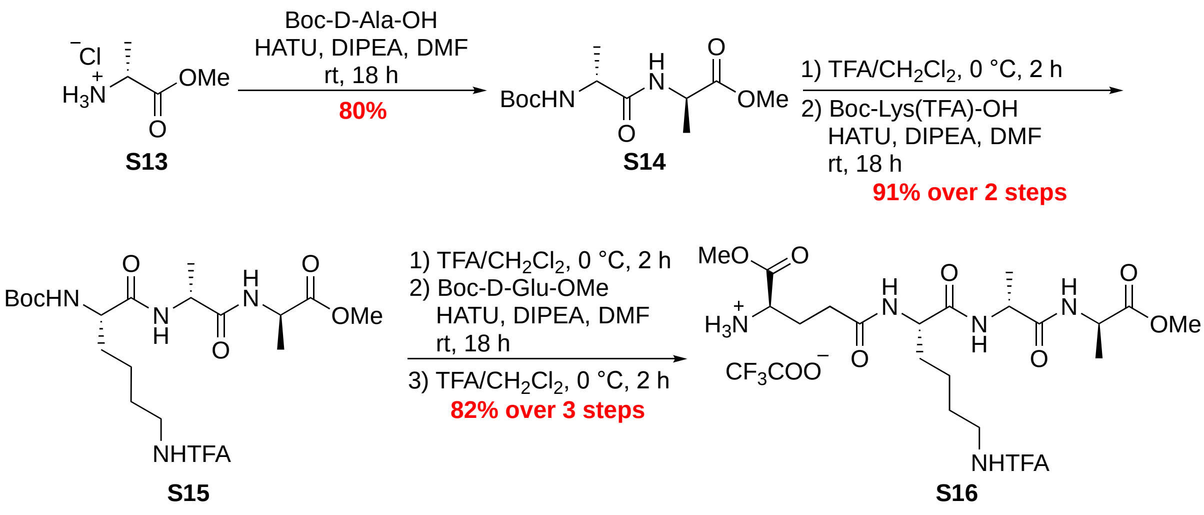

Scheme S7. Synthesis of tetrapeptide **S16**. Compound **S16** was synthesized using our previously established synthetic method.[1]

*Boc-D-Ala-D-Ala-OMe (**S14**)*

H-D-Ala-OMe·HCl **S13** (5.0 g, 35.8 mmol), Boc-D-Ala-OH (6.78 g, 35.8 mmol), and HATU (13.6 g, 35.8 mmol) were dissolved in 100 mL of dry DMF and cooled to 0 °C. Subsequently, 6.25 mL of DIPEA (107.4 mmol) was added, and the reaction mixture was stirred at room temperature for 18 h. The solution was then concentrated under reduced pressure, re-dissolved in 200 mL of EtOAc, and washed with 100 mL of 1 M HCl, 100 mL of saturated sodium bicarbonate, and 100 mL of brine. The organic phase was dried over anhydrous sodium sulfate and concentrated under reduced pressure. The crude material was dissolved in 100 mL of $CHCl_3$, filtered through celite, and concentrated under reduced pressure to obtain Boc-dipeptide **S14** as a white foam (7.86 g, 80%). ^{1}H-NMR ($CDCl_3$, 400 MHz) δ 6.70 (br, 1H, D-Ala5NH), 5.07 (br, 1H, D-Ala4NH), 4.57 (pentet, J = 7.3 Hz, 1H, D-Ala5Hα), 4.18 (br, 1H, D-Ala4Hα), 3.75 (s, 3H, D-Ala5-OMe), 1.45 (m, 9H, Boc), 1.41 (d, J = 7.2 Hz, 3H, D-Ala5Hβ), 1.36 (d, J = 7.1 Hz, 3H, D-Ala4Hβ). ^{13}C-NMR ($CDCl_3$, 100 MHz) δ 173.2, 172.2, 52.5, 48.0, 28.3, 18.3, 18.2.

*Boc-Lys-D-Ala-D-Ala-OMe (**S15**)*

Boc-dipeptide **S14** (2.1 g, 7.66 mmol) was dissolved in 20 mL of CH_2Cl_2 and cooled to 0 °C. Subsequently, 30 mL of TFA was added, and the reaction mixture was stirred at room temperature for 2 h. The reaction mixture was then concentrated under reduced pressure, azeotroped with toluene, and dried under a high vacuum for 1 h. Simultaneously, in a separate flask, Boc-Lys(TFA)-OH (2.6 g, 7.66 mmol) and HATU (2.9 g, 7.66 mmol) were dissolved in 30 mL of dry DMF and cooled to 0 °C. 4.0 mL of DIPEA (22.98 mmol) was added, and the

resulting yellow solution was stirred at 0 °C for 15 minutes. The deprotected dipeptide was dissolved in 7 mL of DMF and added to the activated acid solution. The resulting reaction mixture was stirred at room temperature for 18 h and then concentrated under reduced pressure. The resulting oil was redissolved in 100 mL of EtOAc, washed with 100 mL of 1M HCl, 100 mL of saturated aqueous $NaHCO_3$, and 100 mL of brine. After drying over anhydrous Na_2SO_4 and subsequent concentration under reduced pressure, the Boc-tripeptide **S15** was obtained as a white foam (3.5 g, 91%). ^{1}H-NMR (DMSO-d_6, 400 MHz) δ 9.38 (t, 1H, J = 5.8 Hz, Lys3-NHTFA), 8.20 (d, 1H, J = 7.1 Hz, D-Ala4-NH), 8.01 (d, 1H, J = 8.0 Hz, D-Ala5-NH), 6.92 (d, 1H, J = 7.5 Hz, Lys3-NH), 4.37-4.22 (m, 2H, Lys3-Hα + D-Ala4-Hα), 3.87 (q, J = 7.3 Hz, 1H, D-Ala5-Hα), 3.61 (s, 3H, OMe), 3.15 (q, J = 6.7 Hz, 2H, Lys3-Hε), 1.61-1.42 (m, 4H, Lys3-Hβ + Lys3-Hδ), 1.37 (s, 9H, *t*Bu), 1.31-1.13 (m, 8H, Lys3-Hγ + D-Ala4-Hβ + D-Ala5-Hβ); ^{13}C-NMR (DMSO-d_6, 100 MHz) δ 173.2, 172.4, 172.2, 156.6 (d, J = 35.9), 155.9, 116.5 (q, J = 288.3), 78.6, 54.7, 52.3, 48.0, 47.9, 39.5, 31.7, 28.6, 28.4, 23.1, 18.6, 17.2.

*H-γ-D-Glu(α-OMe)-Lys(TFA)-D-Ala-D-Ala-OMe trifluoroacetate salt (**S16**)*

Boc-Tripeptide **S15** (2.7 g, 5.42 mmol) was dissolved in 15 mL of CH_2Cl_2 and cooled to 0 °C. Subsequently, 15 mL of TFA was added, and the solution was stirred at room temperature for 2 h. The reaction mixture was then concentrated under reduced pressure, azeotroped with toluene, and dried under a high vacuum for 1 h. Concurrently, in a separate flask, Boc-γ-D-Glu(α-OMe)-OH (1.45 g, 5.42 mmol) and HATU (2.1 g, 5.42 mmol) were dissolved in 20 mL of dry DMF and cooled to 0 °C. Then, 2.83 mL of DIPEA (16.26 mmol) was added, and the resulting yellow solution was stirred at 0 °C for 15 minutes. The deprotected tripeptide was dissolved in 8 mL of DMF and added to the activated acid solution. The resulting reaction mixture was stirred at room temperature for 18 h and then concentrated under reduced pressure. The resulting oil was redissolved in 50 mL of EtOAc and 2 mL of DMF, washed with 50 mL of 1 M HCl, 50 mL of saturated aqueous $NaHCO_3$, and 100 mL of brine. After drying

over anhydrous Na_2SO_4 and subsequent concentration under reduced pressure, the Boc-tetrapeptide was obtained as a white powder. The crude Boc-tetrapeptide was suspended in 10 mL of CH_2Cl_2, and 10 mL of TFA was added. The reaction mixture was stirred for 2 hours at room temperature, concentrated under reduced pressure, and subjected to azeotroping with MeOH (2 × 8 mL) and CH_2Cl_2 (2 × 10 mL), resulting in the formation of the tetrapeptide **S16** as an off-white solid (2.91 g, 82%). ^{1}H-NMR (DMSO-d_6, 400 MHz) δ 9.42 (t, 1H, J = 5.7 Hz, Lys3-NHTFA), 8.46 (br. s, 3H, D-Glu2-H_3N^+), 8.26 (d, 1H, J = 7.0 Hz, D-Ala4-NH), 8.21 (d, 1H, J = 7.9 Hz, D-Ala5-NH), 8.13 (d, 1H, J = 7.7 Hz, Lys3-NH), 4.35-4.19 (m, 4H, D-Glu2-Hα + Lys3-Hα + D-Ala4-Hα + D-Ala5-Hα), 3.73 (s, 3H, D-Glu2-OMe), 3.61 (s, 3H, D-Ala5-OMe), 3.15 (q, 2H, J = 6.6 Hz, Lys3-Hε), 2.41-2.23 (m, 2H, D-Glu-Hγ), 2.06-1.91 (m, 2H D-Glu-Hβ), 1.67-1.39 (m, 4H, Lys3-Hβ + Lys3-Hδ), 1.31-1.14 (m, 8H, Lys3-Hγ + Ala4-Hβ + Ala5-Hβ); ^{13}C-NMR (DMSO-d_6, 100 MHz) δ 173.3, 172.5, 171.7, 171.3, 170.2, 158.8 (q, J = 33.6), 156.6 (q, J = 35.8), 117.0 (q, J = 296.4), 116.4 (q, J = 288.2), 53.2, 53.0, 52.3, 52.1, 48.0, 39.5, 32.1, 30.7, 28.4, 26.4, 23.0, 18.5, 17.2.

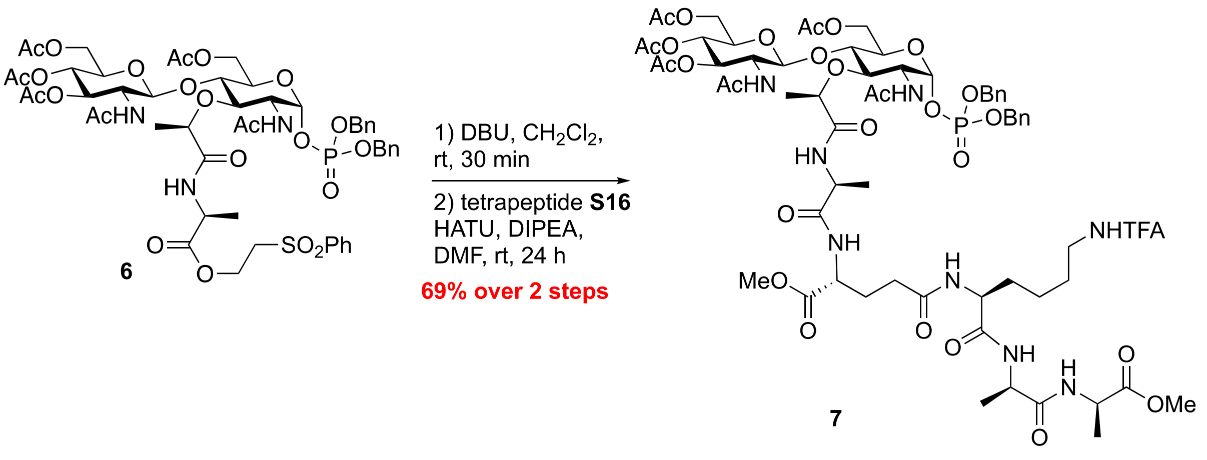

Scheme S8. Synthesis of GlcNAc-MurNAc disaccharide pentapeptide core **7**. Compound **7** was synthesized using our previously established synthetic method.[1]

N-[N-Acetyl-6-O-acetyl-1-O-[bis(phenylmethoxy)phosphinyl]-4-O-[3,4,6-tri-O-acetyl-2-(acetylamino)-2-deoxy-β-D-glucopyranosyl]-α-muramoyl]-alanyl-D-γ-glutamyl-N6-(2,2,2-trifluoroacetyl)-lysyl-D-alanyl-D-alanyl-2,5-dimethyl ester (**7**)

Disaccharide **6** (700 mg, 0.6 mmol) was dissolved in 6 mL of dry CH_2Cl_2 and stirred at room temperature under argon. A solution of diazabicycloundec-7-ene (90 μL, 0.6 mmol) was added, and the resulting solution was stirred for 30 minutes. The reaction mixture was then diluted with 30 mL of CH_2Cl_2, washed with 10 mL of 1 M HCl and 15 mL of brine, dried over anhydrous Na_2SO_4, and concentrated under reduced pressure. The crude oil was precipitated with Et_2O and dried under a high vacuum for 2 h, resulting in the formation of the corresponding acid as a white solid. The acid was dissolved in 10 mL of dry DMF and cooled to 0 °C using an ice bath. Next, HATU (228 mg, 0.6 mmol), followed by 314 μL of DIPEA (1.8 mmol), was added, and the resulting yellow solution was stirred for 15 minutes. Subsequently, tetrapeptide **S16** (382 mg, 0.6 mmol) was added, and the resulting solution was stirred at room temperature for 24 h. The reaction mixture was then concentrated under reduced pressure and re-dissolved in a mixture of $CHCl_3$ and IPA (9:1, 20 mL), washed with 10 mL of 1 M HCl and 10 mL of saturated sodium bicarbonate. Both aqueous washes were back-extracted with $CHCl_3$ (3 × 5 mL), and the combined organic extracts were washed with 2 × 5 mL of brine, dried over anhydrous sodium sulfate, and concentrated under reduced pressure. The crude product precipitated from Et_2O, yielding the pentapeptide disaccharide **7** as an off-white solid (628 mg, 69%), which was used directly in the next step without further purification. HRMS (ESI) Calcd for $C_{65}H_{91}F_3N_8PO_2$ $[M+H]^+$ 1519.5632, found 1519.5546.

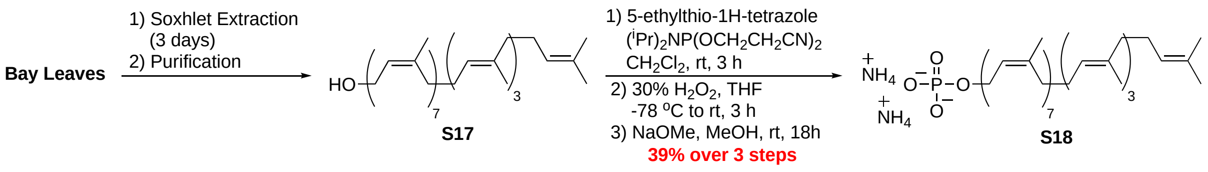

Scheme S9. Synthesis of undecaprenyl phosphate bisammonium salt (**S18**). Alcohol **S17** was isolated using a protocol previously established by our research group.[9] Phosphate **S18** was synthesized using our previously established synthetic method.[1]

*Undecaprenol (**S17**)*

Ground bay leaves (500 g, *Laurus nobilis*) were subjected to soxhlet extraction using 1200 mL of refluxing petroleum ether for 2 days. After this, methanol (300 mL) and anhydrous K_2CO_3 (20 g) were added, and the resulting mixture was stirred at room temperature for 3 days. Filtration through glass wool separated solids from the extract solution, resulting in the concentration of the extract to a sticky green tar. This crude extract was then dissolved in approximately 200 mL of a 5:95 mixture of EtOAc and hexanes. The entire solution was loaded onto a glass column containing 1 kg of silica gel, pre-wetted with a 5:95 EtOAc/hexanes mixture, for further purification. Fractions containing undecaprenol were isolated by comparison with a commercial standard from American Radiolabeled Chemicals, combined, and concentrated under reduced pressure to obtain crude undecaprenol in the form of orange oil. The crude undecaprenol was dissolved in pyridine (5 mL) and acetic anhydride (10 mL), and the mixture was stirred at room temperature for 6 h. The resulting solution was diluted with brine (50 mL) and extracted with EtOAc (3 × 50 mL). The organic phase underwent washing with 1 M HCl (50 mL), saturated aqueous $NaHCO_3$ (50 mL), and brine (50 mL), followed by drying over anhydrous Na_2SO_4 and concentration under reduced pressure. The crude material was subsequently subjected to flash column chromatography using silica gel and a 3:97 mixture of EtOAc and hexanes as the eluent, leading to the isolation of undecaprenyl acetate as a light-yellow oil. Undecaprenyl acetate was dissolved in a mixture of 3:2 THF and MeOH (72 mL), and anhydrous K_2CO_3 (5.0 g) was added. The resulting suspension was stirred for 18 h at room temperature, then diluted with hexanes (100 mL), and washed with water

(100 mL) and brine (100 mL). The organic phase was dried over anhydrous Na_2SO_4 and concentrated under reduced pressure, yielding pure undecaprenol **S17** (1.1 g) as a light-yellow oil. The spectroscopic data were in agreement with our previously reported data.[9] ^{1}H-NMR ($CDCl_3$, 400 MHz) δ 5.44 (td, *J* = 7.2, 1.5 Hz, 1H, C<u>H</u>CH_2OH), 5.17-5.06 (m, 10H, prenyl alkene protons); 4.09 (t, 2H, *J* = 6.2, C<u>H</u>$_2$OH), 2.11-1.94 (m, 40H), 1.76-1.73 (m, 3H), 1.70-1.66 (m, 21H), 1.62-1.59 (m, 12H); HRMS (ESI) Calcd for $C_{55}H_{90}NaO$ $[M+Na]^+$ 789.6884, found 789.6851.

*Undecaprenyl phosphate bisammonium salt (Und-P, **S18**)*

Undecaprenol (**S17**, 356 mg, 0.50 mmol) was dissolved in anhydrous CH_2Cl_2 (5 mL) and rapidly added *via* a syringe into a vigorously stirred suspension of 5-ethylthio-1*H*-tetrazole (285 mg, 2.21 mmol) and bis(2-cyanoethyl)-*N*,*N*'-diisopropylphosphoramidite (0.37 mL, 1.43 mmol) in anhydrous CH_2Cl_2 (5 mL) under argon at room temperature. After 3 h, the mixture was diluted with CH_2Cl_2 (40 mL) and washed with saturated sodium bicarbonate (20 mL), water (20 mL), and brine (25 mL). The organic solution was dried over anhydrous sodium sulfate and concentrated under reduced pressure to obtain the phosphite in the form of a yellow oil. This product was dissolved in THF (10 mL) and cooled to −78 °C. Hydrogen peroxide (30%, 1.0 mL) was slowly added dropwise via a syringe into the vigorously stirred solution. After the completion of the addition, the ice bath was removed, and the mixture was allowed to warm to room temperature over 2 h. The reaction mixture was then diluted with ice-cold saturated sodium sulfite (5 mL) and stirred at 0 °C for 5 minutes. After this, the reaction mixture was extracted with EtOAc (40 mL), and the organic layer was washed with saturated $NaHCO_3$ (30 mL), water (30 mL), and brine (50 mL). Subsequently, it was dried over anhydrous sodium sulfate and concentrated under reduced pressure, resulting the phosphate as a yellow oil. The crude phosphate was suspended in anhydrous MeOH (8 mL), and a 25% NaOMe in MeOH

solution (0.35 mL) was added. The mixture was stirred at room temperature for 16 hours, and then further diluted with MeOH (15 mL) and $CHCl_3$ (15 mL). To neutralize it, DOWEX 50WX8 H+ form resin was used. After filtering the resin, the filtrate was concentrated under reduced pressure. The resulting yellow oil was subjected to purification through silica gel column chromatography using a gradient solvent system of $CHCl_3$, MeOH, H_2O, and NH_4OH (90:10:0:0.1 to 65:25:5:0.1), resulting in the formation of Und-P **S18** as a light-yellow oil. The spectroscopic data are consistent with our previous findings documented in scientific literature.[1] HRMS (ESI) Calcd for $C_{55}H_{90}O_4P$ $[M-H]^-$ 845.6577, found 845.6598.

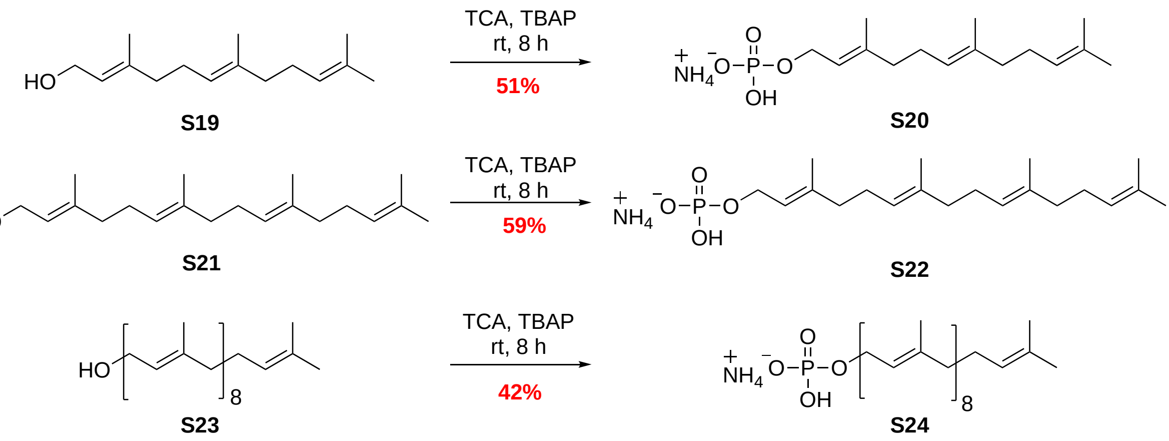

Scheme S10. Synthesis of farnesyl (**S20**), geranylgeranyl (**S22**), and solanesyl (**S24**) phosphates. The aforementioned phosphates were synthesized following established protocols outlined in the literature.[10]

*Farnesyl phosphate **(S20)***

Farnesol (**S19**, 200 mg, 0.9 mmol) is dissolved in acetonitrile (1 mL), and trichloroacetonitrile (0.23 mL, 2.25 mmol) is added, followed by the dropwise addition of tetrabutylammonium dihydrogenphosphate (611 mg, 1.8 mmol) in acetonitrile (5 mL). The reaction mixture is stirred at room temperature for 8 h. After removing the solvent under

vacuum, the crude material is preliminarily purified using silica gel flash chromatography (isopropanol/NH_4OH/H_2O 7:2:1). The fractions from the column, showing the presence of phosphate via TLC (with PMA), are combined and concentrated under reduced pressure. For further purification, a Dowex 50WX8 ion-exchange column is loaded and equilibrated with a mixture of NH_4OH and H_2O in a 3:1 ratio. The residue left on the silica column is percolated through the DOWEX column using the same NH_4OH buffer, collected, and subsequently dried via lyophilization to obtain farnesyl phosphate **S20** as a fluffy white solid (138 mg, 51%). The spectroscopic data were in agreement with those previously reported.[10] ^{31}P-NMR ($CDCl_3$, 162 MHz) δ 0.46; HRMS (ESI) Calcd for $C_{15}H_{26}O_4P$ $[M-H]^-$ 301.1569, found 301.1578.

*Geranylgeranyl phosphate **(S22)***

Geranylgeranyl phosphate **S22** was synthesized using a method like that described for farnesyl phosphate and isolated as a fluffy off-white solid (75 mg, 59%). The spectroscopic data were in agreement with those previously reported.[11] ^{31}P-NMR ($CDCl_3$, 162 MHz) δ 0.46; HRMS (ESI) Calcd for $C_{20}H_{34}O_4P$ $[M-H]^-$ 369.2195, found 369.2190.

*Solanesyl phosphate **(S24)***

Solanesyl phosphate **S24** was synthesized using a method like that described for farnesyl phosphate and isolated as a fluffy light-yellow solid (94 mg, 42%). The spectroscopic data were in agreement with those previously reported.[12] ^{31}P-NMR ($CDCl_3$, 162 MHz) δ 1.72; HRMS (ESI) Calcd for $C_{45}H_{74}O_4P$ $[M-H]^-$ 709.5325, found 709.5425.

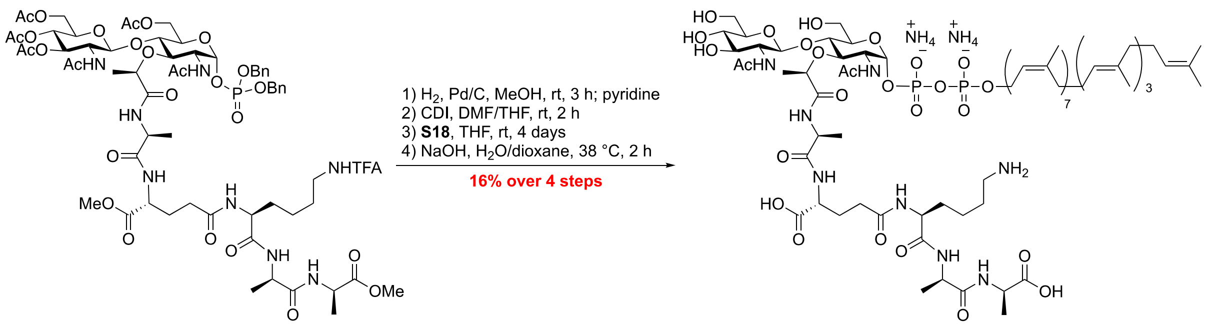

Scheme S11: Synthesis of lipid II diammonium salt (**11**). Lipid II (**11**) was synthesized using our previously established method.[1]

*Lipid II diammonium salt (**11**)*

In a sequential process, dibenzyl phosphate **7** (75 mg, 50 µmol) was dissolved in anhydrous MeOH (8 mL) with the flask purged using an argon balloon. Pd/C (10 wt %/w, 159 mg, 149 µmol) was introduced, and the resulting suspension was stirred under an H_2 atmosphere for 3 h. After filtration through celite, with MeOH washes (2 × 4 mL), pyridine (0.5 mL) was added to the filtrate, which was then concentrated in vacuo and subjected to high vacuum for 1 h to obtain the sugar-phosphate salt as a white solid. This salt was dissolved in dry DMF (1.5 mL) and dry THF (1.5 mL), followed by the addition of carbonyl diimidazole (40.2 mg, 247 µmol). The clear solution was stirred at room temperature for 3 h. Excess carbonyl diimidazole was quenched with dry MeOH (10.7 µL, 264 µmol), and stirring continued for 45 minutes. After concentration in vacuo and drying under high vacuum for 1 h, the resulting activated phosphate was combined with a solution of Und-P (**S18**) (45 mg, 50 µmol) in THF (2 mL) and 5-ethylthio-1*H*-tetrazole (6.4 mg, 50 µmol). The mixture was stirred under argon at room temperature for 96 h, followed by concentration in vacuo. To this crude mixture, 1,4-dioxane (2 mL) and a solution of sodium hydroxide (60 mg, 1 mmol) in water (2 mL) were added. The resulting mixture was stirred at 38 °C for 2 h, and filtered through an aqueous filter disc with a wash of 1:1 H_2O/1,4-dioxane (2 mL). The crude lipid II was

subsequently purified by HPLC using a Phenomenex Luna C_{18}(2) 100 Å prep-scale column with a flow rate of 10 mL/min, UV detection at 220 nm, and a gradient of solvent A (50 mM aqs. NH_4HCO_3) and solvent B (MeOH). The eluted product-containing fractions were concentrated by rotary evaporation, diluted with H_2O, frozen, and lyophilized to yield Gram-positive lipid II (**11**) as a fluffy white powder (20.1 mg, 16% over 4 steps). Retention time (tR): 31.049 min; The spectroscopic data were in agreement with our previously reported data.[1] ^{31}P-NMR (CD_3OD: $CDCl_3$ 1:1 162 MHz) δ -12.86, -10.44; HRMS (ESI) Calcd for $C_{94}H_{154}N_8O_{26}P_2$ $[M-2H]^{2-}$ 936.5225, found 936.5221.

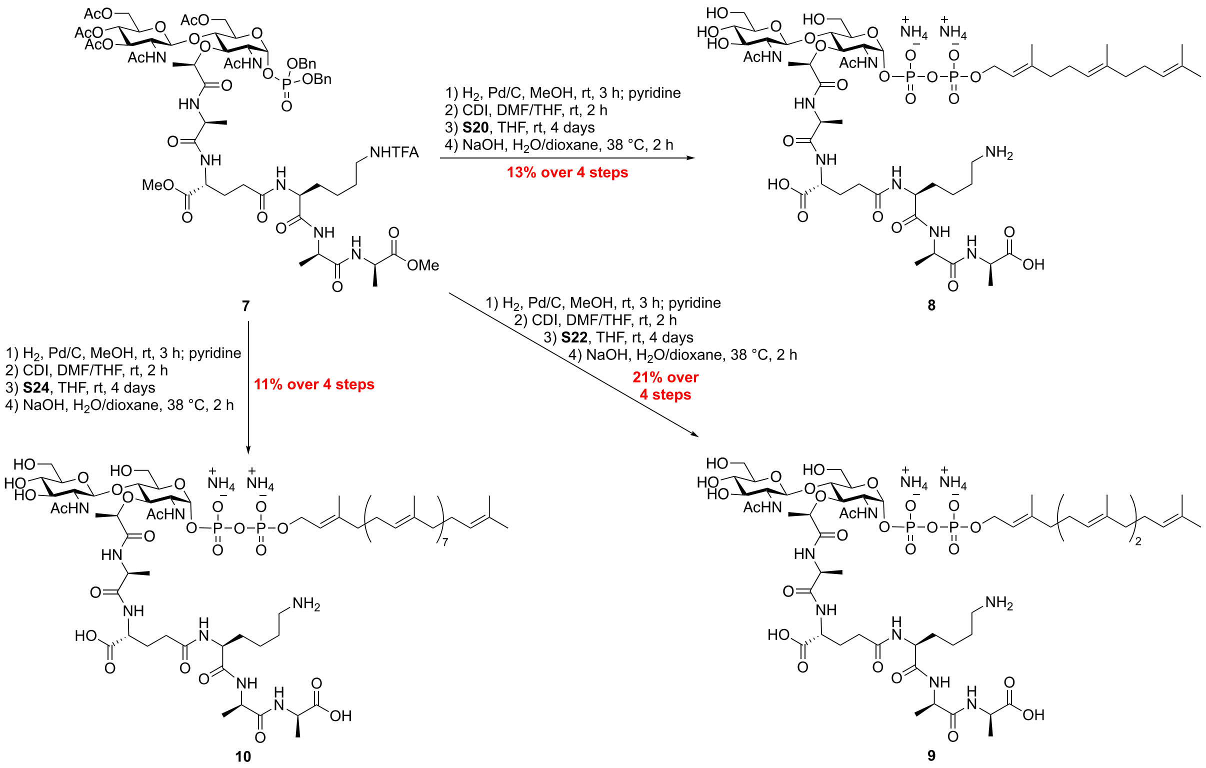

Scheme S12: Synthesis of farnesyl (**8**) geranylgeranyl (**9**), and solanesyl (**10**) analogues of lipid II. The aforementioned lipid II analogues were synthesized using our previously

established method.[1] Note, the final products **8**–**11** proved too insoluble to obtain suitable ^{1}H or ^{13}C NMR data. This difficulty in NMR analysis of polyprenyl-linked glycosyl diphosphates has been reported by several other groups.[13-15] Characterization is therefore limited to ^{31}P NMR and HRMS.

*Farnesyl lipid II analogue **(8)***

Compound **8** was synthesized using a method like that described for lipid II **11** and isolated as a fluffy white powder (9.5 mg, 13% over 4 steps). Retention time (tR): 28.329 min. The spectroscopic data were in agreement with those previously reported.[16] HRMS (ESI) Calcd for $C_{54}H_{92}N_8O_{26}P_2$ $[M\text{-}H]^-$ 1329.5520, found 1329.5251; $[M\text{-}2H]^{2-}$ 664.2721, found 664.2598.

*Geranylgeranyl lipid II analogue **(9)***

Compound **9** was synthesized using a method like that described for lipid II **11** and isolated as a fluffy white powder (17.8 mg, 21% over 4 steps). Retention time (tR): 32.409 min. The spectroscopic data were in agreement with those previously reported.[17] ^{31}P-NMR (CD_3OD: $CDCl_3$ 1:1 162 MHz) δ -12.66, -10.38; HRMS (ESI) Calcd for $C_{59}H_{100}N_8O_{26}P_2$ $[M\text{-}H]^-$ 1397.6164, found 1397.5920; $[M\text{-}2H]^{2-}$ 698.3034, found 698.2925.

*Solanesyl lipid II analogue **(10)***

Compound **10** was synthesized using a method like that described for lipid II **11** and isolated as a fluffy white powder (14.6 mg, 11% over 4 steps). Retention time (tR): 32.863 min. The spectroscopic data were in agreement with those previously reported.[18] ^{31}P-NMR

(CD_3OD: $CDCl_3$ 1:1 162 MHz) δ -12.58, -10.42; HRMS (ESI) Calcd for $C_{84}H_{140}N_8O_{26}P_2$ [M-H]$^-$ 1737.9276, found 1737.9263; [M-2H]$^{2-}$ 868.4599, found 868.4505.

References

1. Dong, Y. Y.; Wang, H.; Pike, A. C. W.; Cochrane, S. A.; Hamedzadeh, S.; Wyszyński, F. J.; Bushell, S. R.; Royer, S. F.; Widdick, D. A.; Sajid, A.; Boshoff, H. I.; Park, Y.; Lucas, R.; Liu, W.-M.; Lee, S. S.; Machida, T.; Minall, L.; Mehmood, S.; Belaya, K.; Liu, W.-W.; Chu, A.; Shrestha, L.; Mukhopadhyay, S. M. M.; Strain-Damerell, C.; Chalk, R.; Burgess-Brown, N. A.; Bibb, M. J.; Barry Iii, C. E.; Robinson, C. V.; Beeson, D.; Davis, B. G.; Carpenter, E. P. *Cell* **2018**, *175*, 1045-1058.e16.
2. Walczewska, A.; Grzywacz, D.; Bednarczyk, D.; Dawgul, M.; Nowacki, A.; Kamysz, W.; Liberek, B.; Myszka, H. *Beilstein J. Org. Chem.* **2015**, *11*, 869-874.
3. Balmond, E. I.; Benito-Alifonso, D.; Coe, D. M.; Alder, R. W.; McGarrigle, E. M.; Galan, M. C. *Angew. Chem. Int. Ed.* **2014**, *53*, 8190-8194.
4. Grann Hansen, S.; Skrydstrup, T. *Eur. J. Org. Chem.* **2007**, *2007*, 3392-3401.
5. Sudibya, H. G.; Ma, J.; Dong, X.; Ng, S.; Li, L. J.; Liu, X. W.; Chen, P. *Angew. Chem. Int. Ed.* **2009**, *48*, 2723-2726.
6. Deng, S.; Gangadharmath, U.; Chang, C.-W. T. *J. Org. Chem.* **2006**, *71*, 5179-5185.
7. Sun, J.; Han, X.; Yu, B. *Carb. Res.* **2003**, *338*, 827-833.
8. Wang, Y.; Aleiwi, B. A.; Wang, Q.; Kurosu, M. *Organic Letters* **2012**, *14*, 4910-4913.
9. Cochrane, R. V. K.; Alexander, F. M.; Boland, C.; Fetics, S. K.; Caffrey, M.; Cochrane, S. A. *Chem. Commun.* **2020**, *56*, 8603-8606.
10. Lira, L. M.; Vasilev, D.; Pilli, R. A.; Wessjohann, L. A. *Tetrahedron Lett.* **2013**, *54*, 1690-1692.
11. Huang, L. Y.; Huang, S. H.; Chang, Y. C.; Cheng, W. C.; Cheng, T. J. R.; Wong, C. H. *Angew. Chem. Int. Ed.* **2014**, *53*, 8060-8065.
12. Hsu, T.-W.; Hsu, H.-C.; Chan, H.-Y.; Fang, J.-M. *J. Org. Chem.* **2020**, *85*, 12747-12753.
13. Gale, R. T.; Sewell, E. W.; Garrett, T. A.; Brown, E. D. *Chem. Sci.* **2014**, *5*, 3823-3830.
14. Holkenbrink, A.; Koester, D. C.; Kaschel, J.; Werz, D. B. *Eur. J. Org. Chem.* **2011**, *2011*, 6233-6239.
15. Woodward, R.; Yi, W.; Li, L.; Zhao, G.; Eguchi, H.; Sridhar, P. R.; Guo, H.; Song, J. K.; Motari, E.; Cai, L.; Kelleher, P.; Liu, X.; Han, W.; Zhang, W.; Ding, Y.; Li, M.; Wang, P. G. *Nat. Chem. Biol.* **2010**, *6*, 418-423.
16. Wingen, L. M.; Rausch, M.; Schneider, T.; Menche, D. *J. Org. Chem.* **2020**, *85*, 10206-10215.
17. Shih, H. W.; Chang, Y. F.; Li, W. J.; Meng, F. C.; Huang, C. Y.; Ma, C.; Cheng, T. J. R.; Wong, C. H.; Cheng, W. C. *Angew. Chem. Int. Ed.* **2012**, *51*, 10123-10126.
18. Ye, X.-Y.; Lo, M.-C.; Brunner, L.; Walker, D.; Kahne, D.; Walker, S. *J. Am. Chem. Soc.* **2001**, *123*, 3155-3156.

Spectroscopic data of synthesized compounds

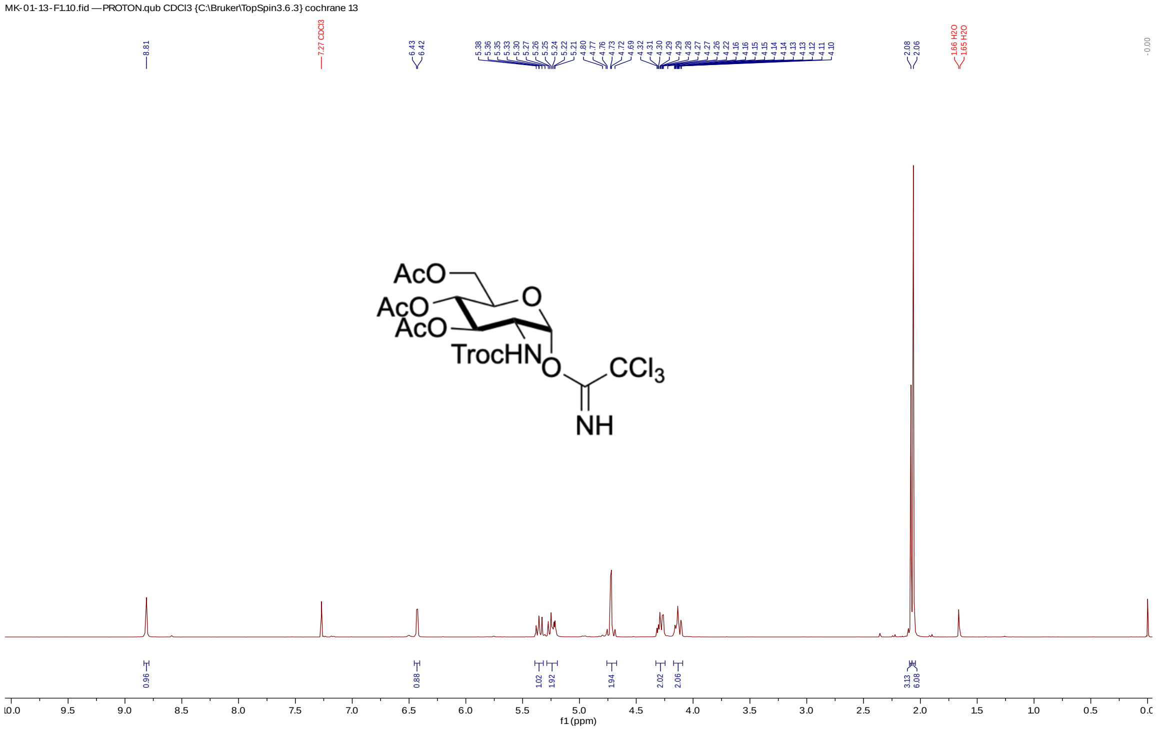

Figure S1: ^{1}H NMR spectrum (400 MHz, $CDCl_3$) of compound **1a**

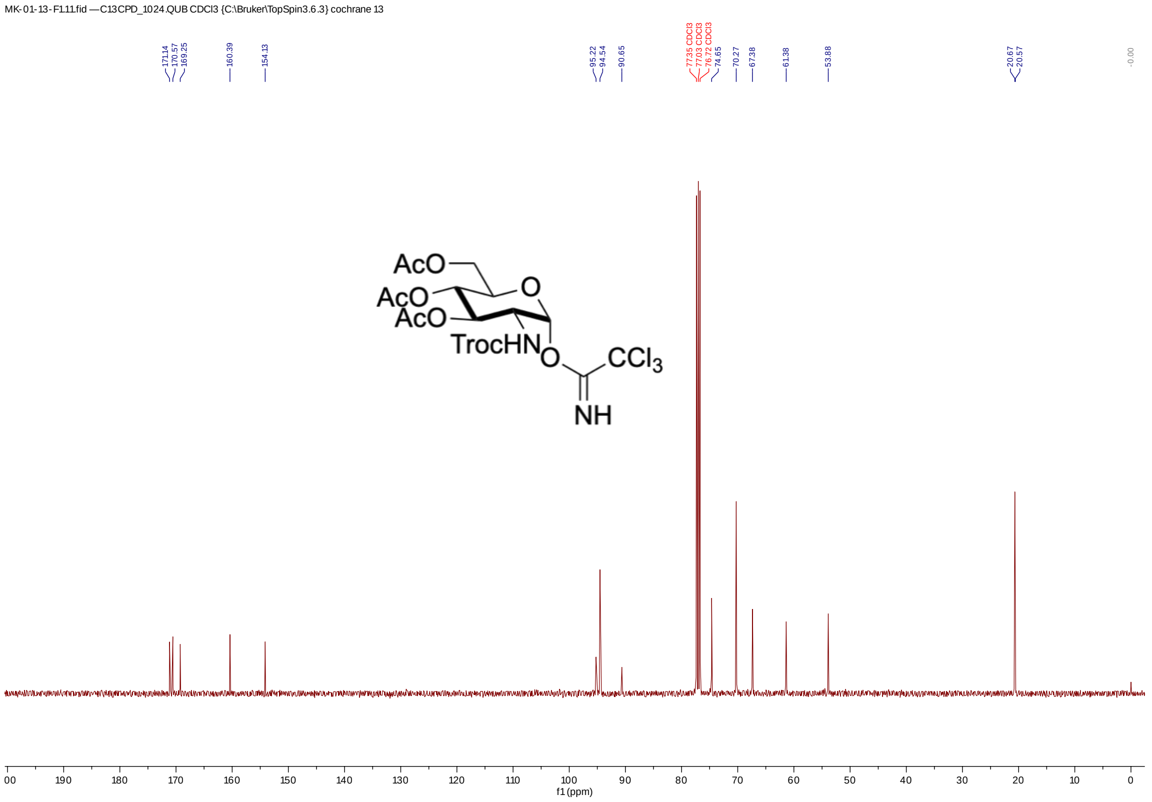

Figure S2: ^{13}C NMR spectrum (100 MHz, $CDCl_3$) of compound **1a**

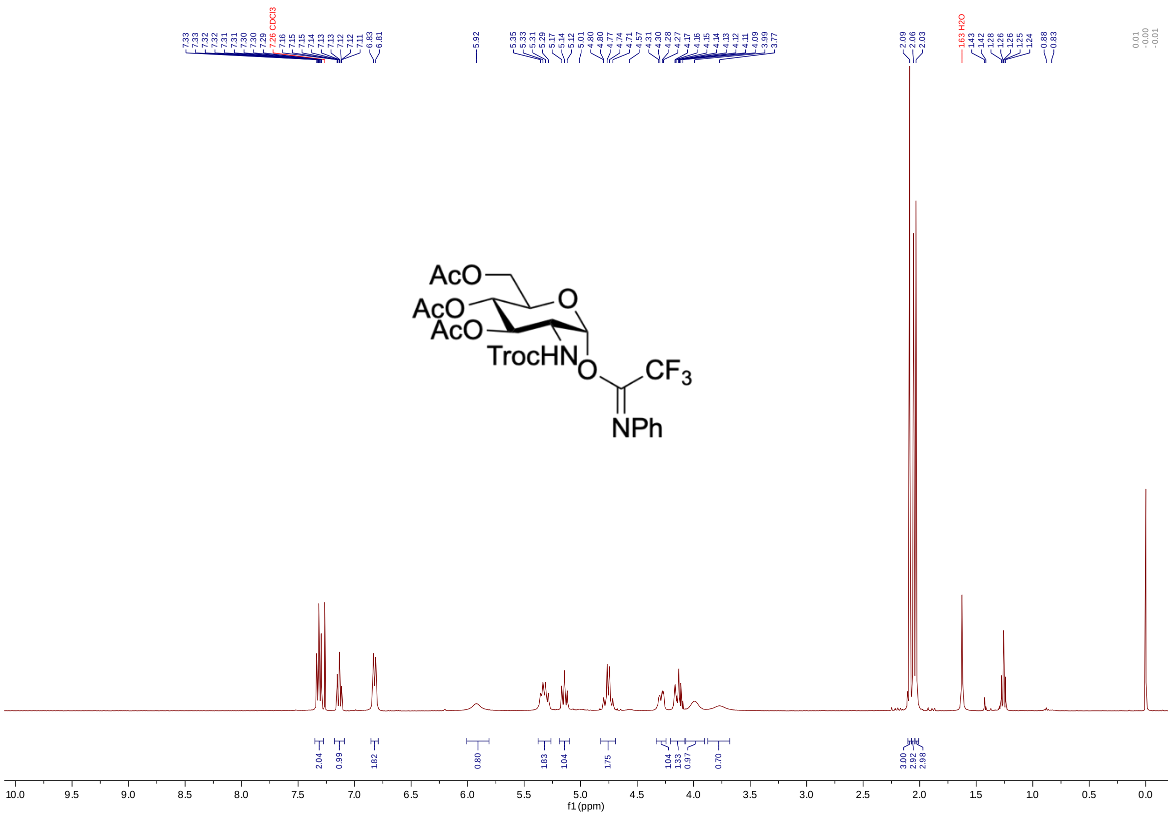

Figure S3: ^{1}H NMR spectrum (400 MHz, $CDCl_3$) of compound **1b**

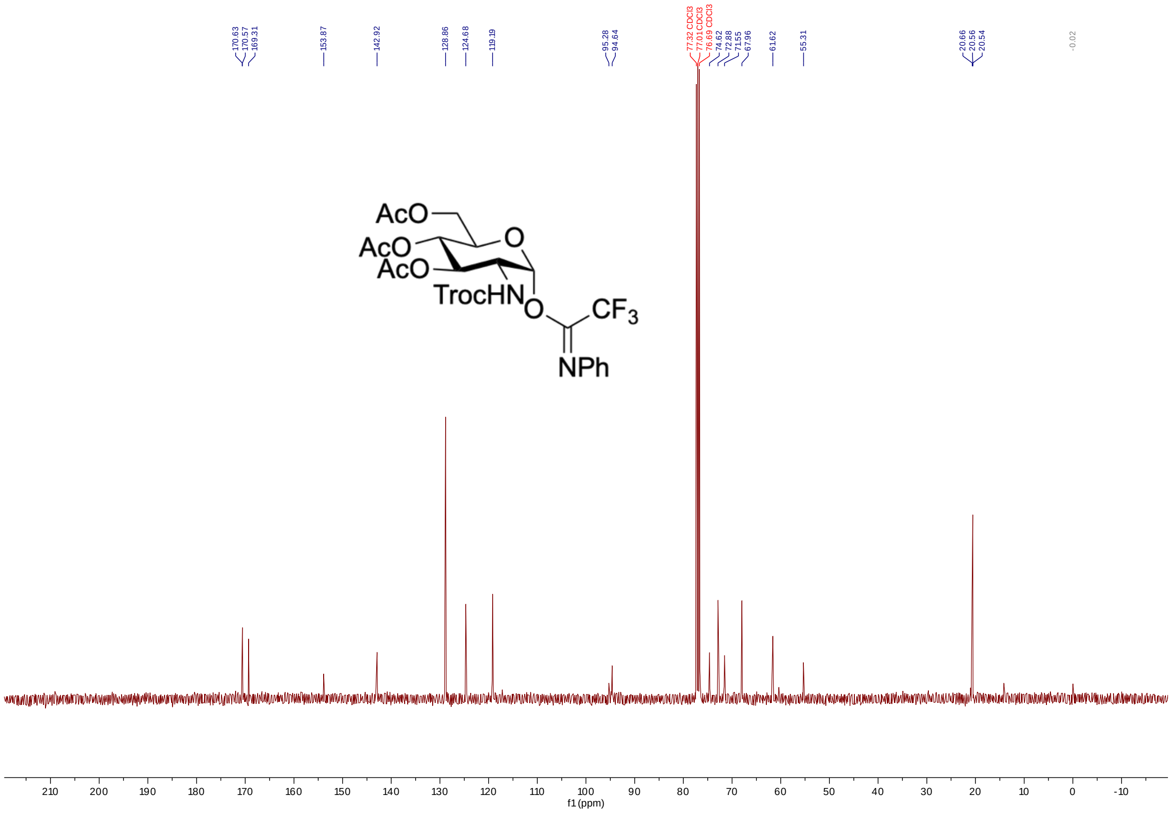

Figure S4: ^{13}C NMR spectrum (100 MHz, $CDCl_3$) of compound **1b**

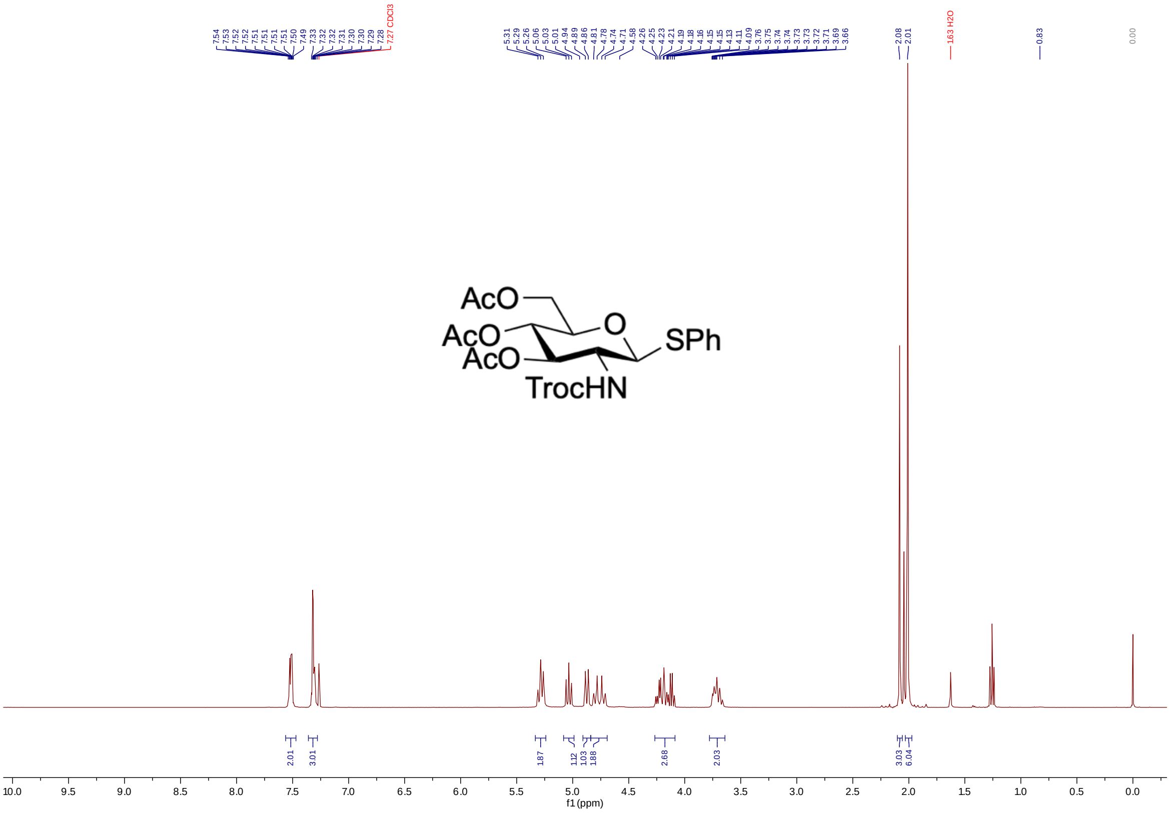

Figure S5: ^{1}H NMR spectrum (400 MHz, $CDCl_3$) of compound **1c**

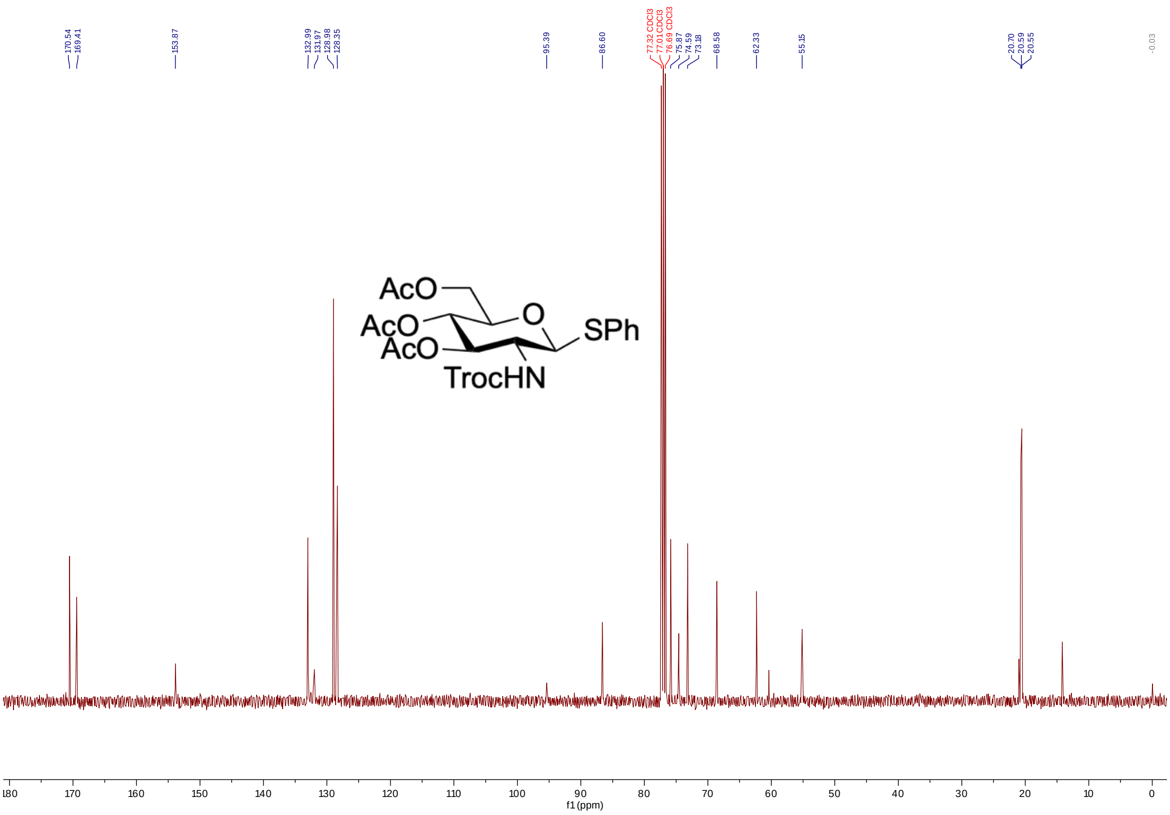

Figure S6: ^{13}C NMR spectrum (100 MHz, $CDCl_3$) of compound **1c**

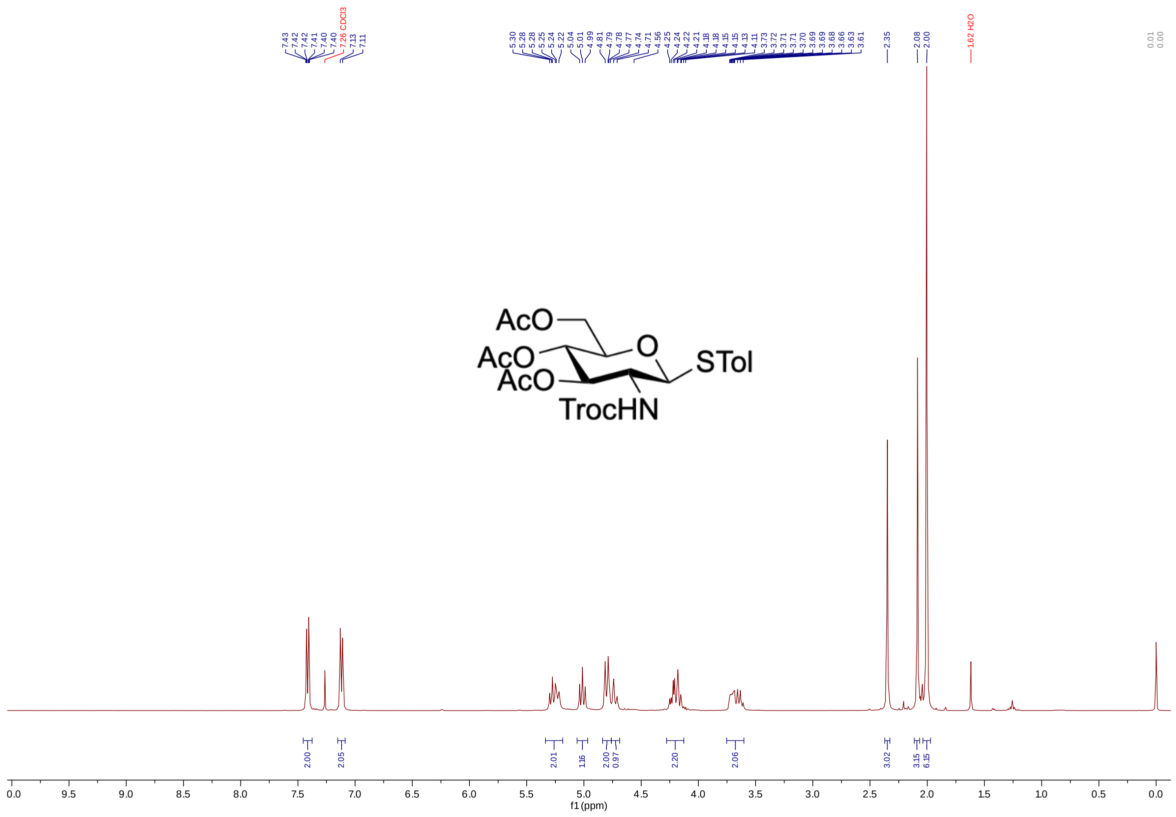

Figure S7: ^{1}H NMR spectrum (400 MHz, $CDCl_3$) of compound **1d**

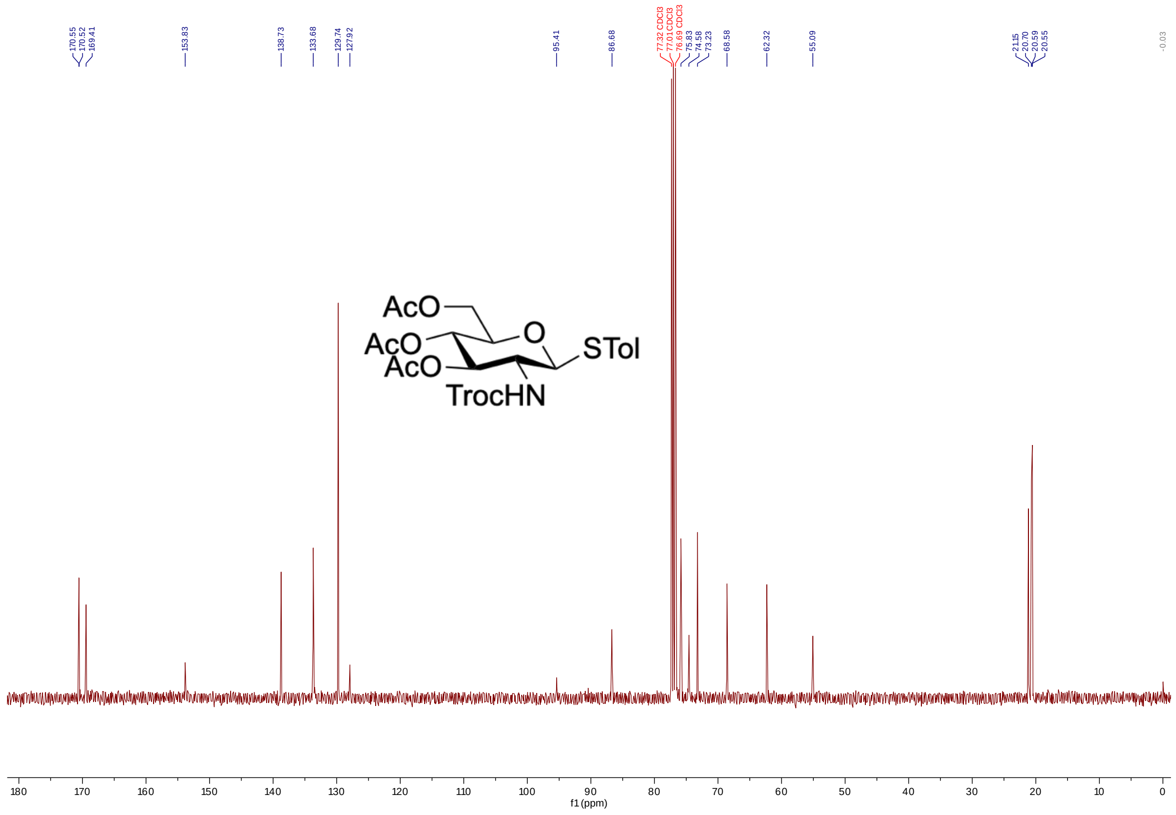

Figure S8: ^{13}C NMR spectrum (100 MHz, $CDCl_3$) of compound **1d**

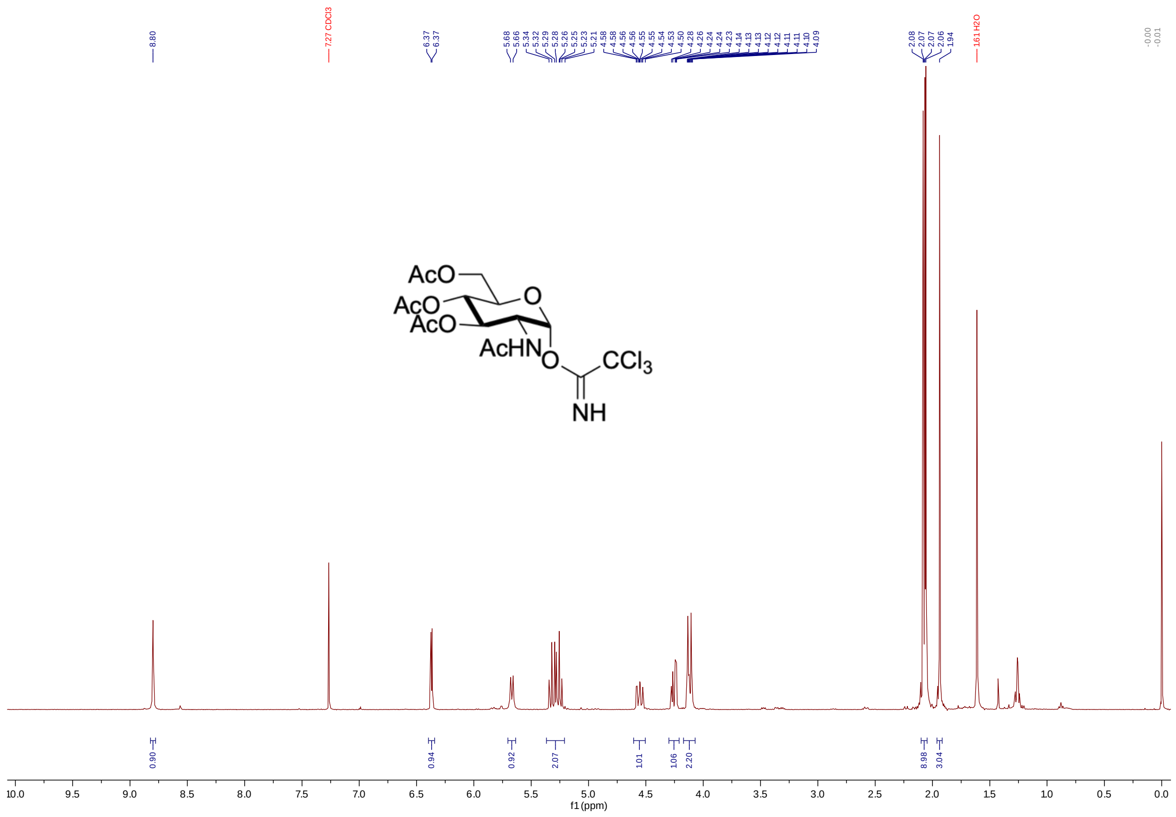

Figure S9: ^{1}H NMR spectrum (400 MHz, $CDCl_3$) of compound **1e**

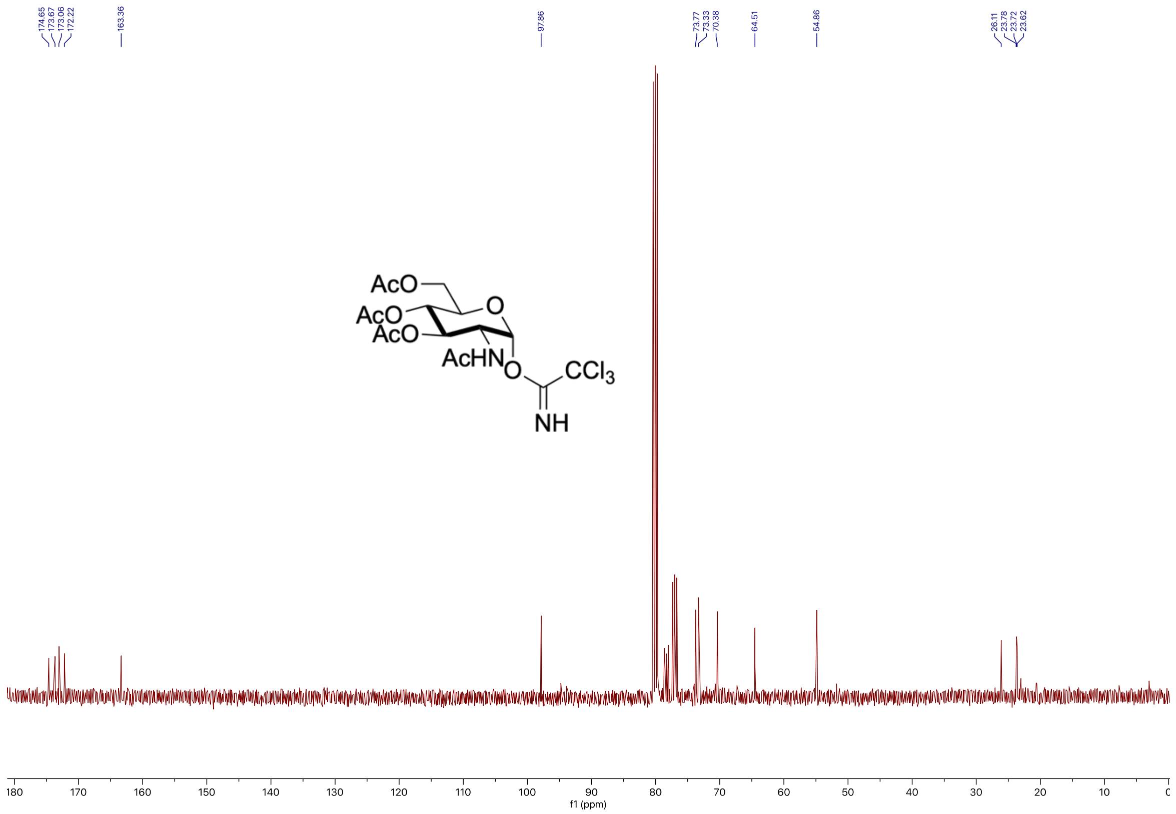

Figure S10: ^{13}C NMR spectrum (100 MHz, $CDCl_3$) of compound **1e**

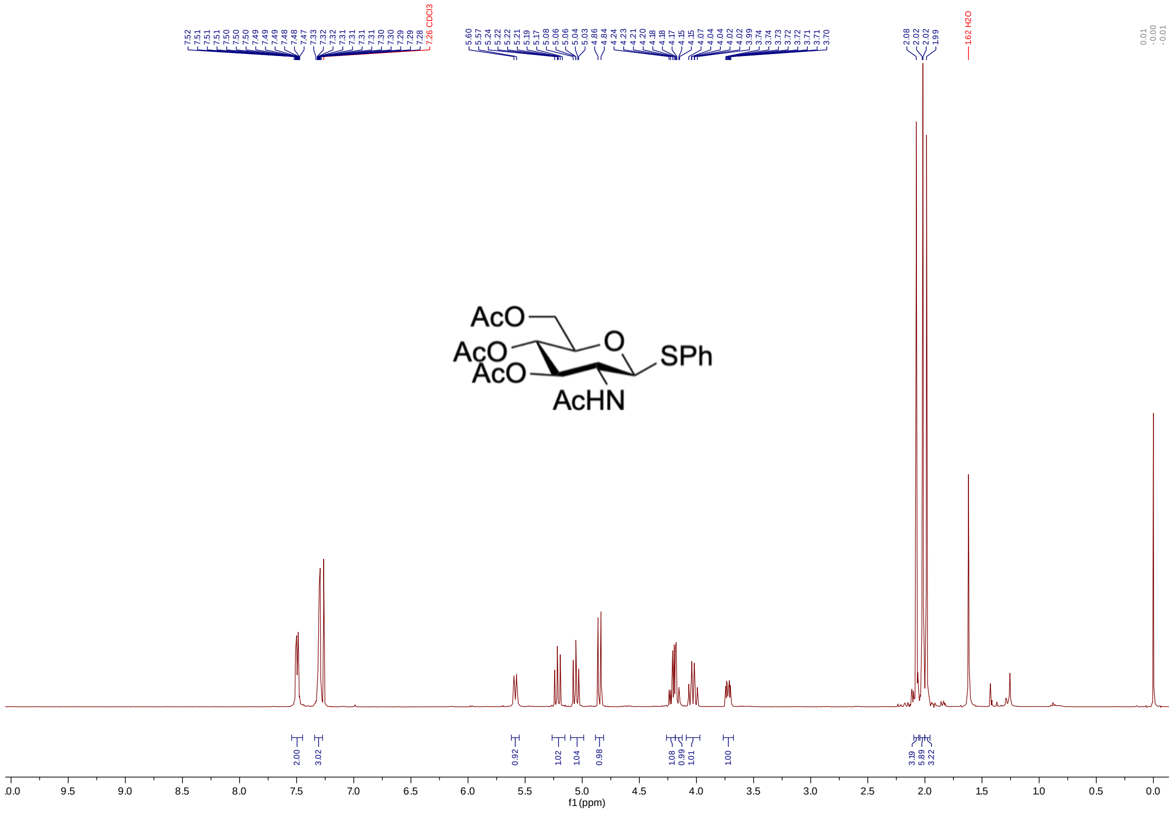

Figure S11: ^{1}H NMR spectrum (400 MHz, $CDCl_3$) of compound **1f**

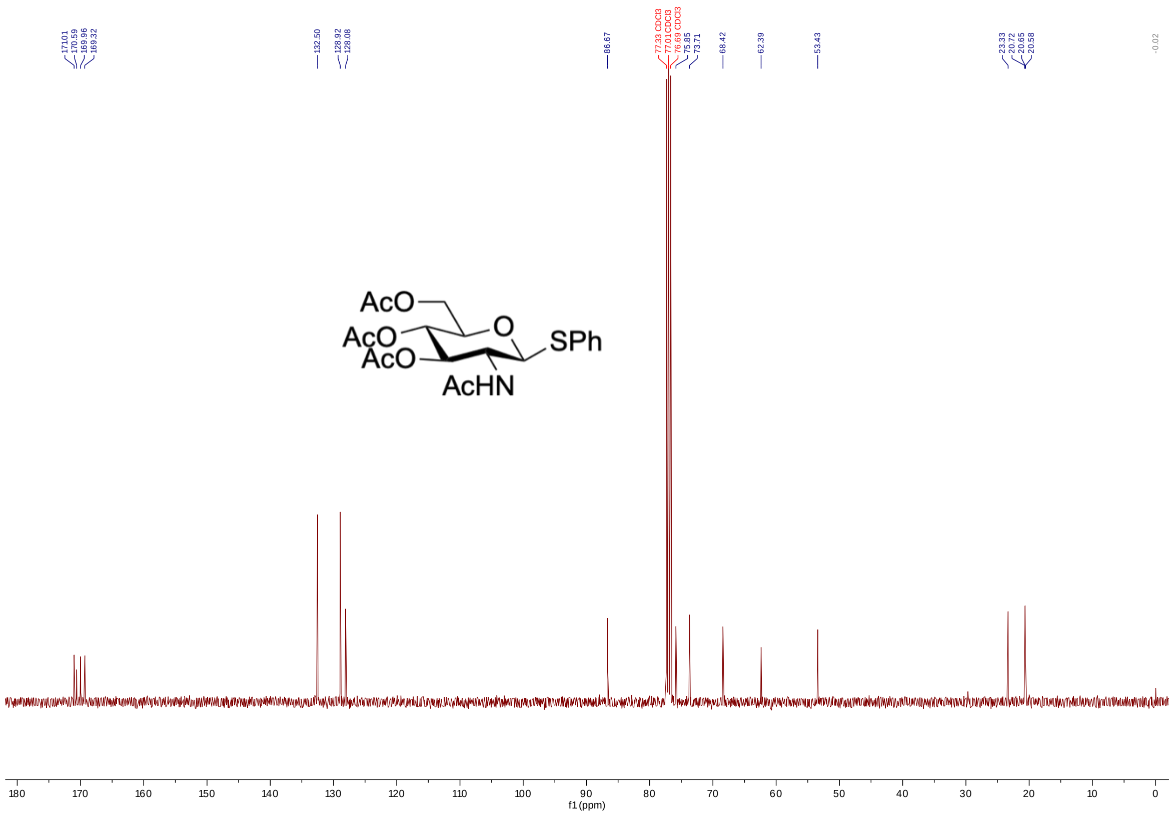

Figure S12: ^{13}C NMR spectrum (100 MHz, $CDCl_3$) of compound **1f**

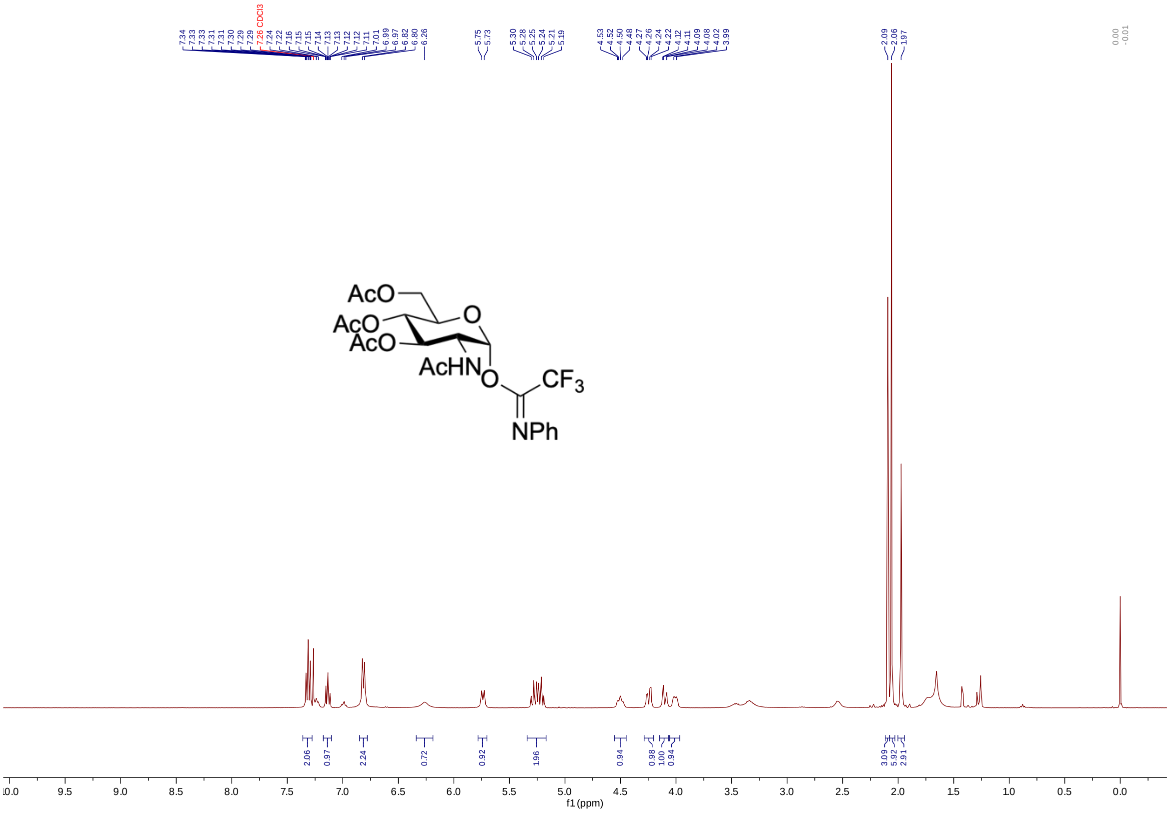

Figure S13: ^{1}H NMR spectrum (400 MHz, $CDCl_3$) of compound **1g**

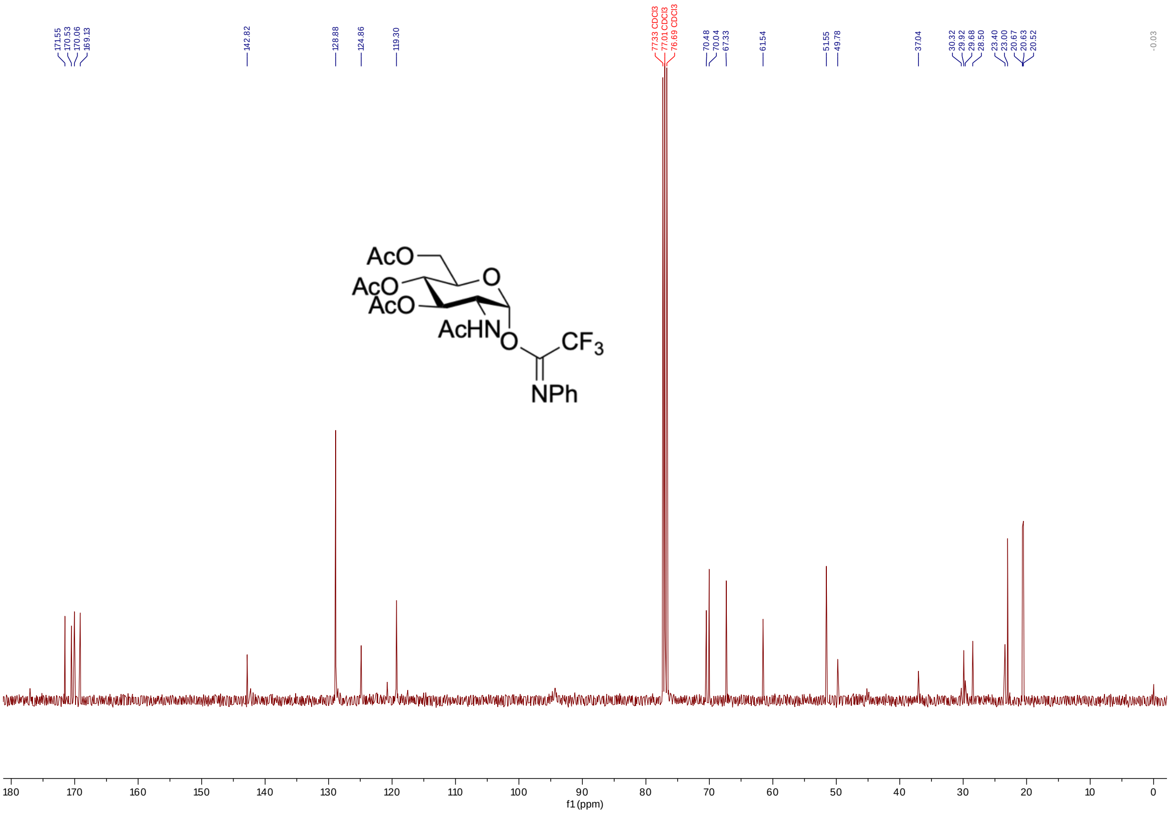

Figure S14: ^{13}C NMR spectrum (100 MHz, $CDCl_3$) of compound **1g**

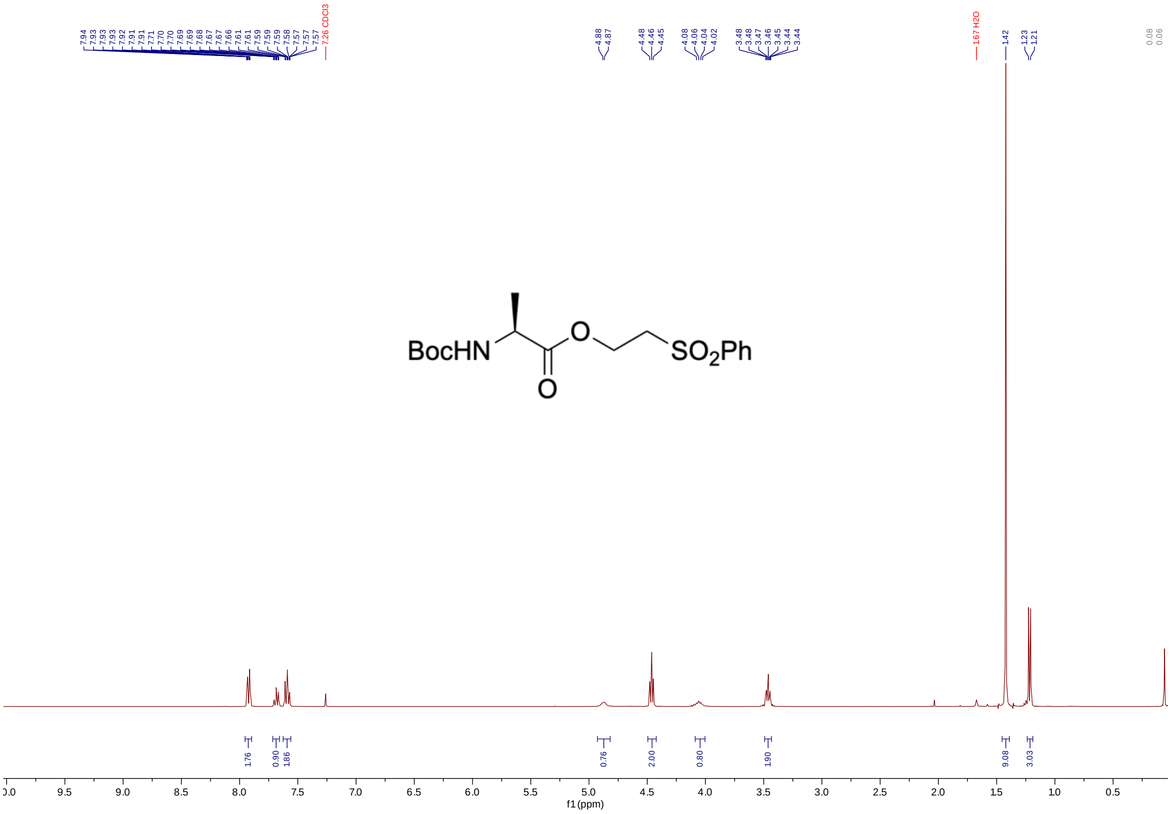

Figure S15: ¹H NMR spectrum (400 MHz, $CDCl_3$) of compound **S6**

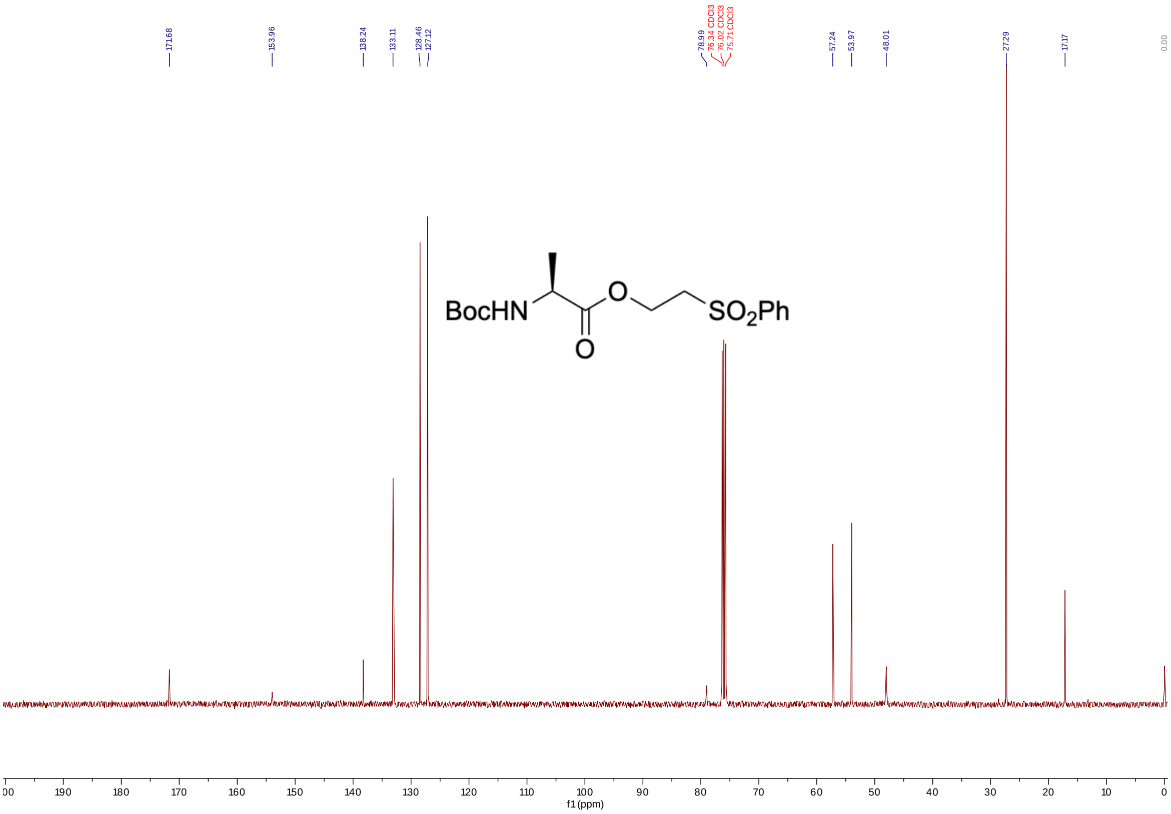

Figure S16: ¹³C NMR spectrum (100 MHz, $CDCl_3$) of compound **S6**

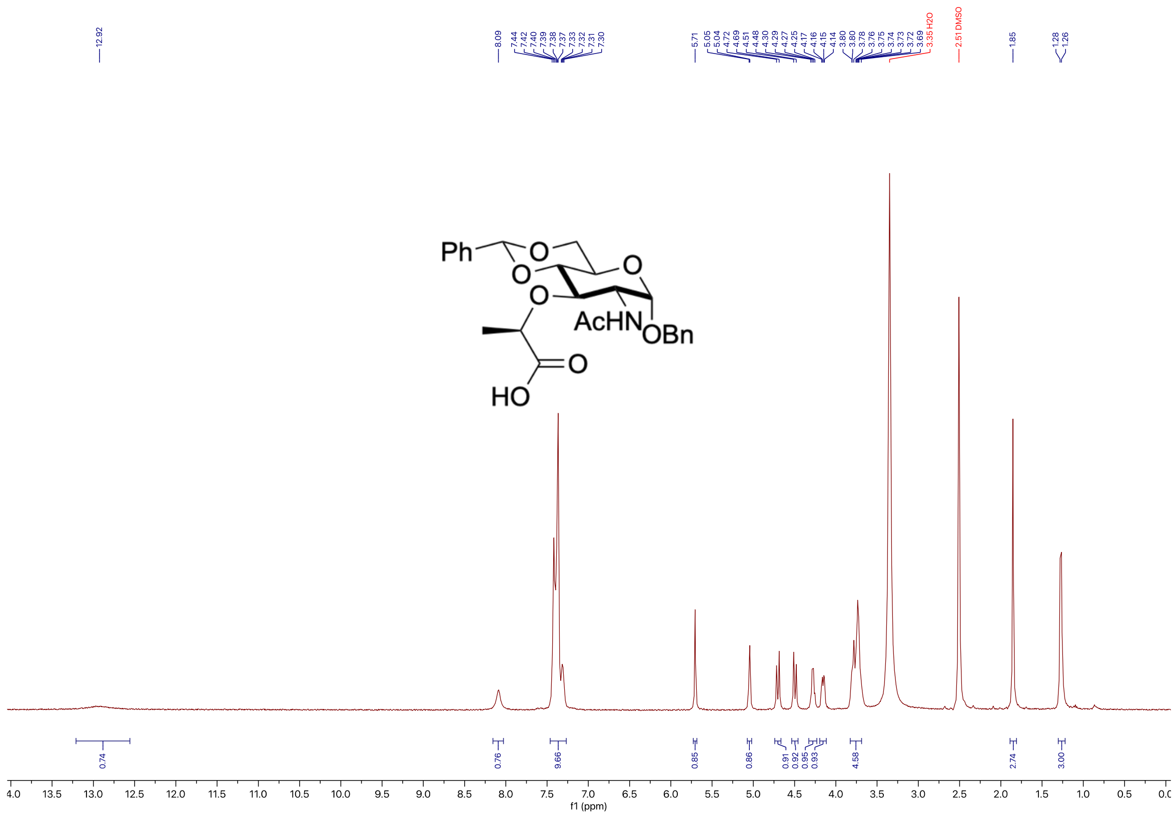

Figure S17: ^{1}H NMR spectrum (400 MHz, DMSO-d_6) of compound **S10**

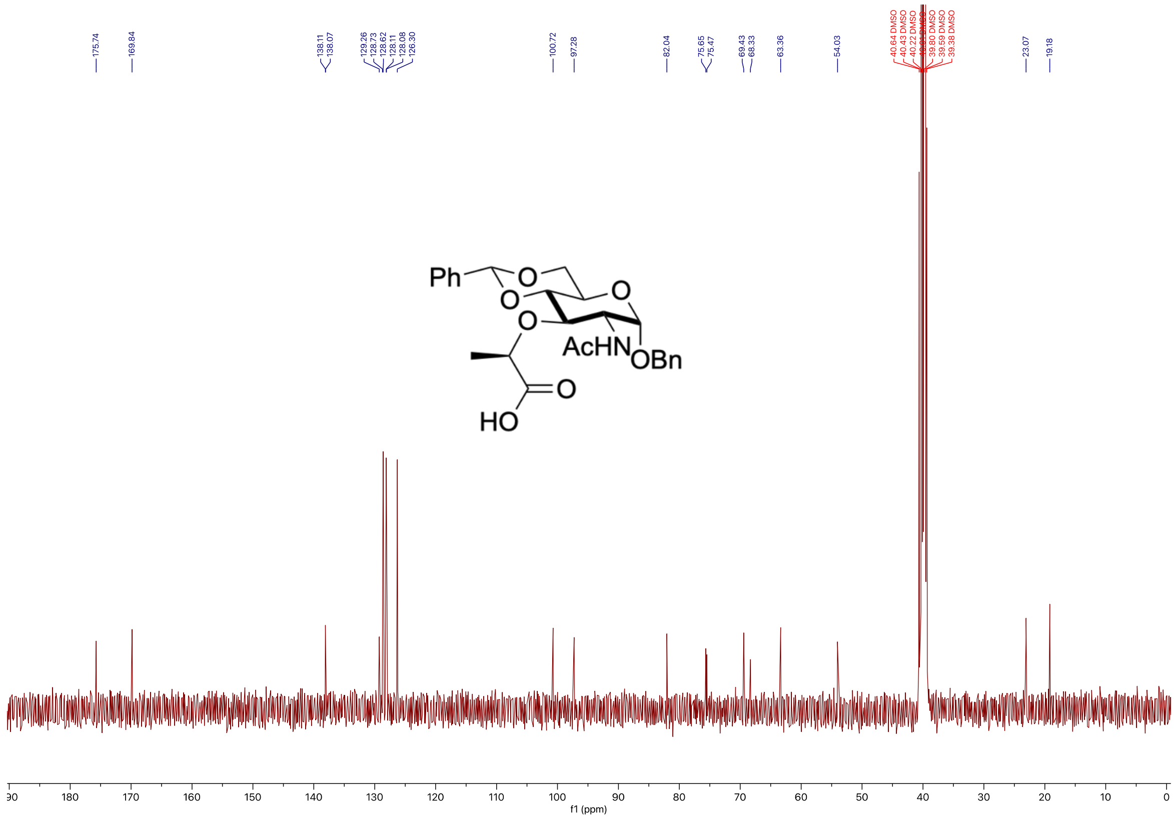

Figure S18: ^{13}C NMR spectrum (100 MHz, DMSO-d_6) of compound **S10**

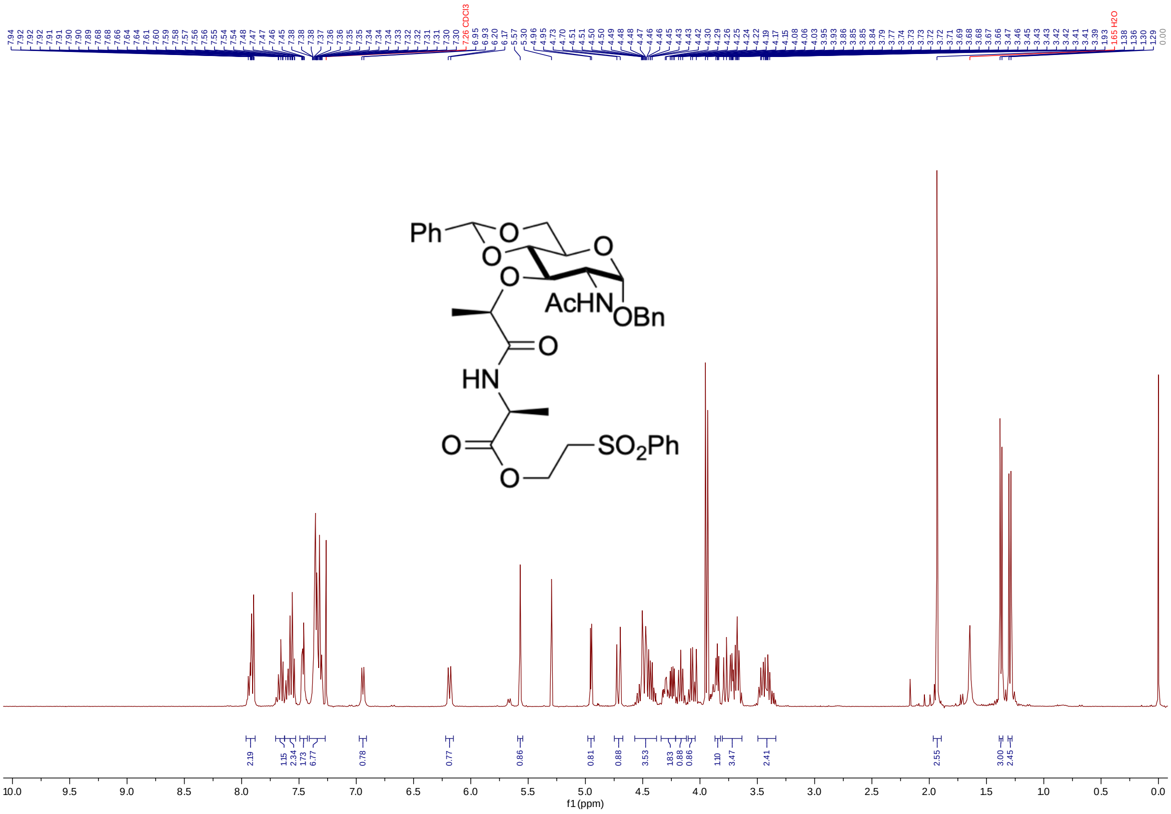

Figure S19: ^{1}H NMR spectrum (400 MHz, $CDCl_3$) of compound **S11**

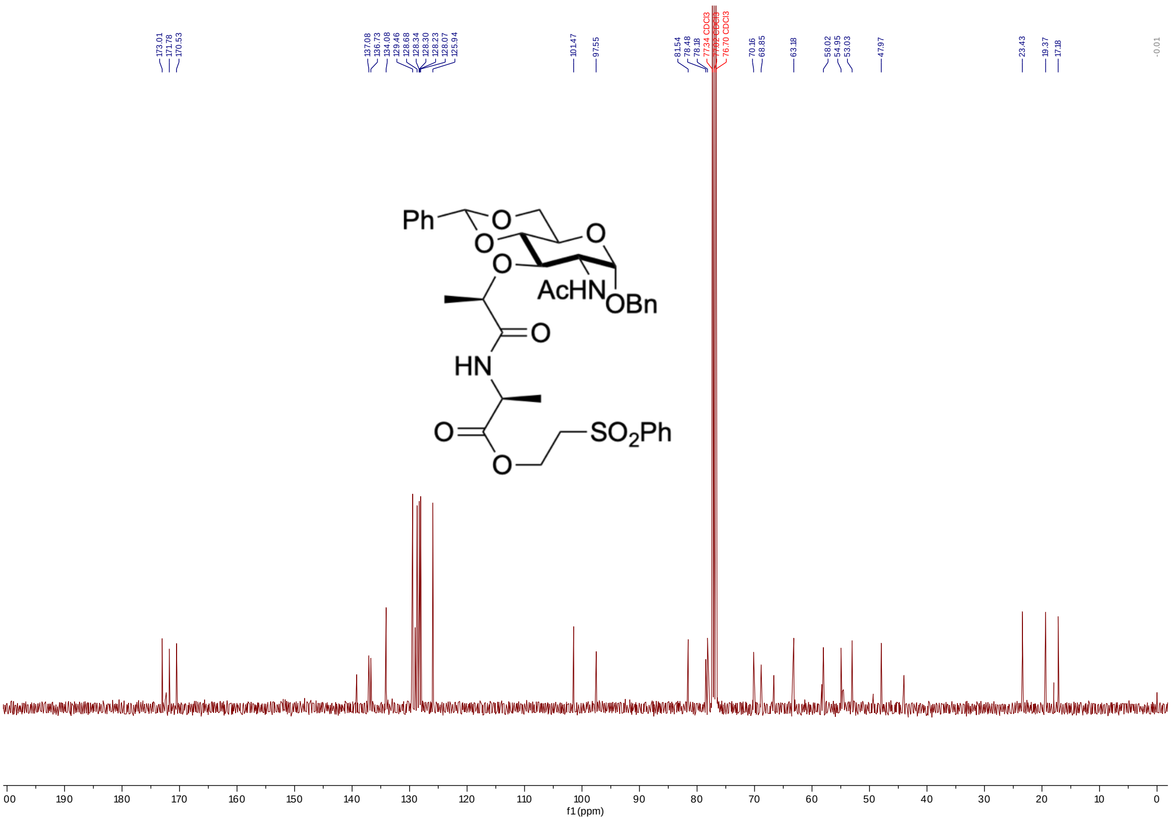

Figure S20: ^{1}H NMR spectrum (100 MHz, $CDCl_3$) of compound **S11**

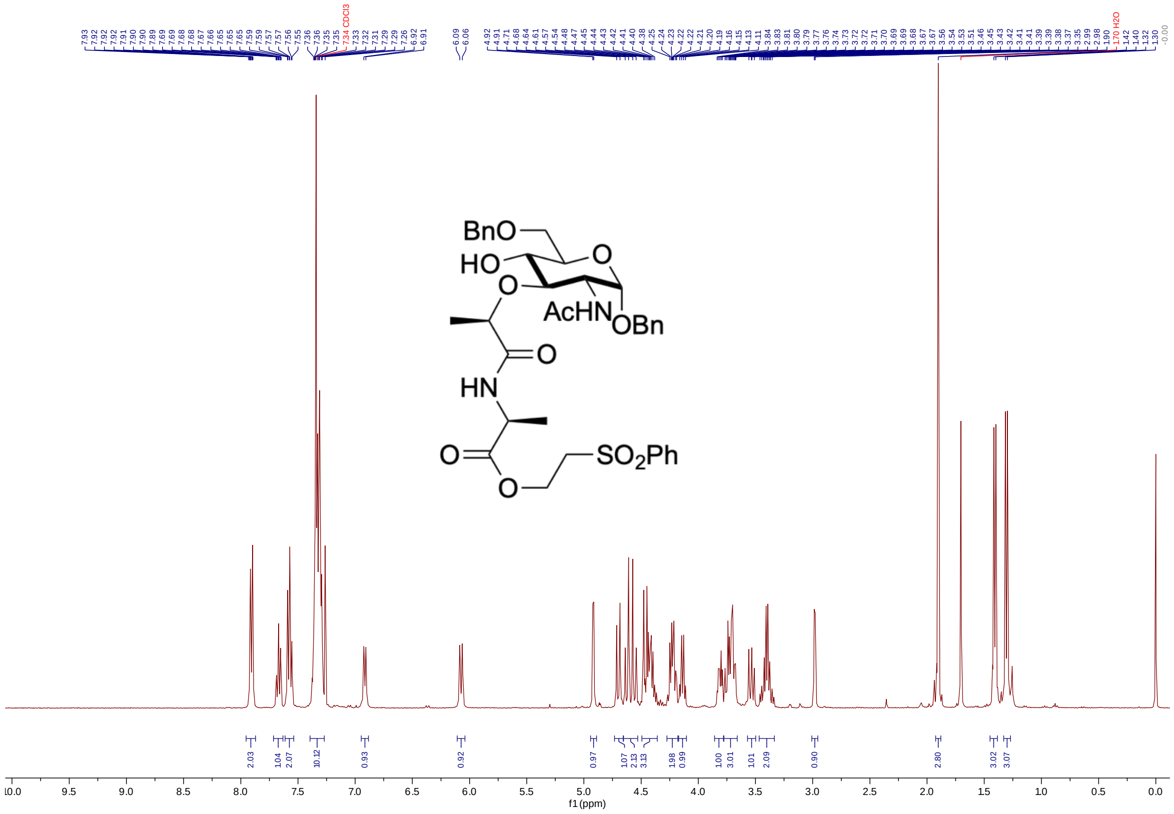

Figure S21: ^{1}H NMR spectrum (400 MHz, $CDCl_3$) of compound **2a**

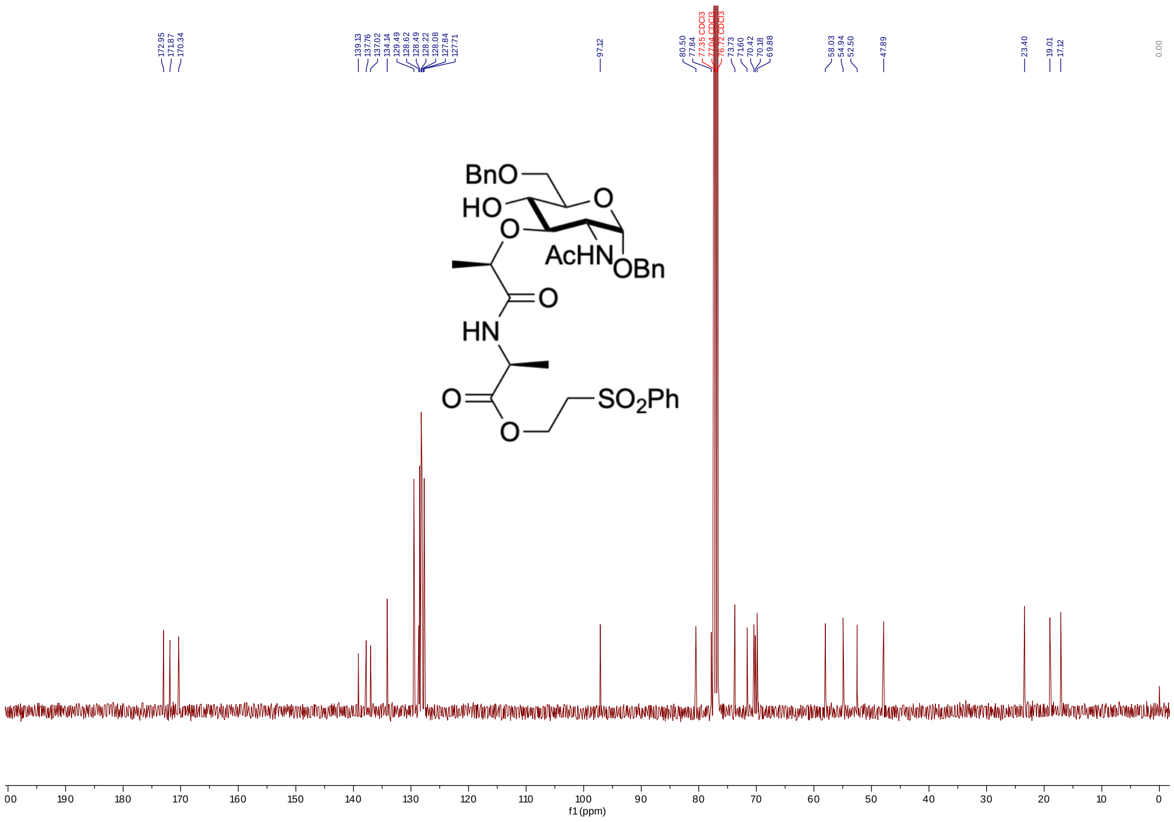

Figure S22: ^{13}C NMR spectrum (100 MHz, $CDCl_3$) of compound **2a**

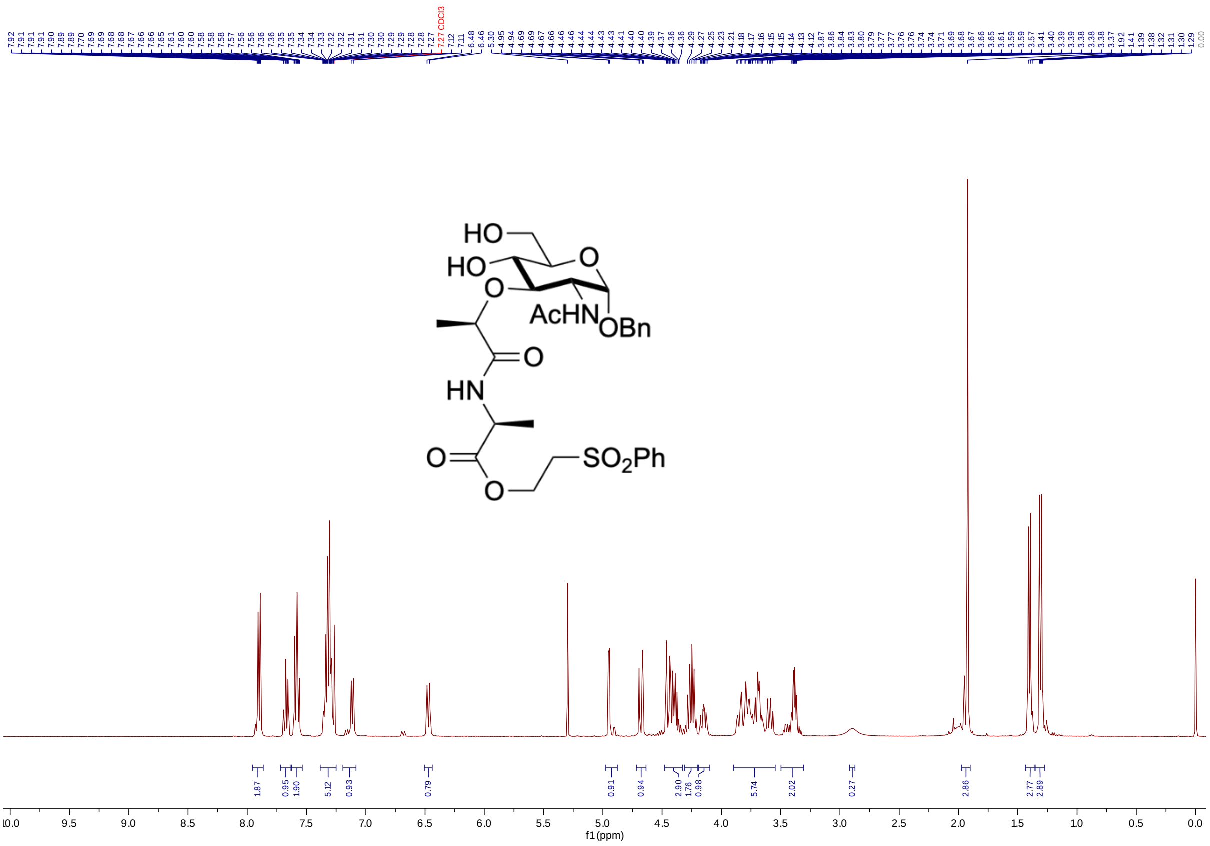

Figure S23: ^{1}H NMR spectrum (400 MHz, $CDCl_3$) of compound **S12**

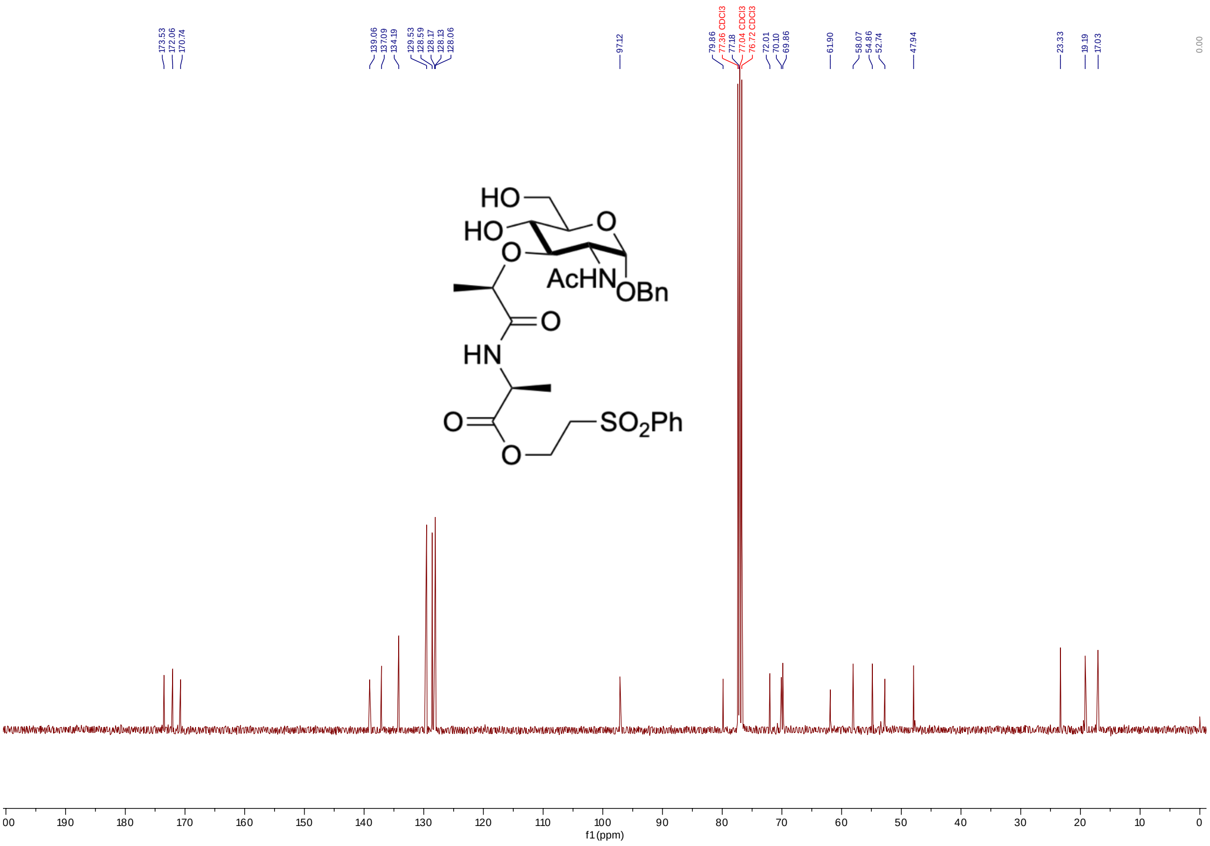

Figure S24: ^{13}C NMR spectrum (100 MHz, $CDCl_3$) of compound **S12**

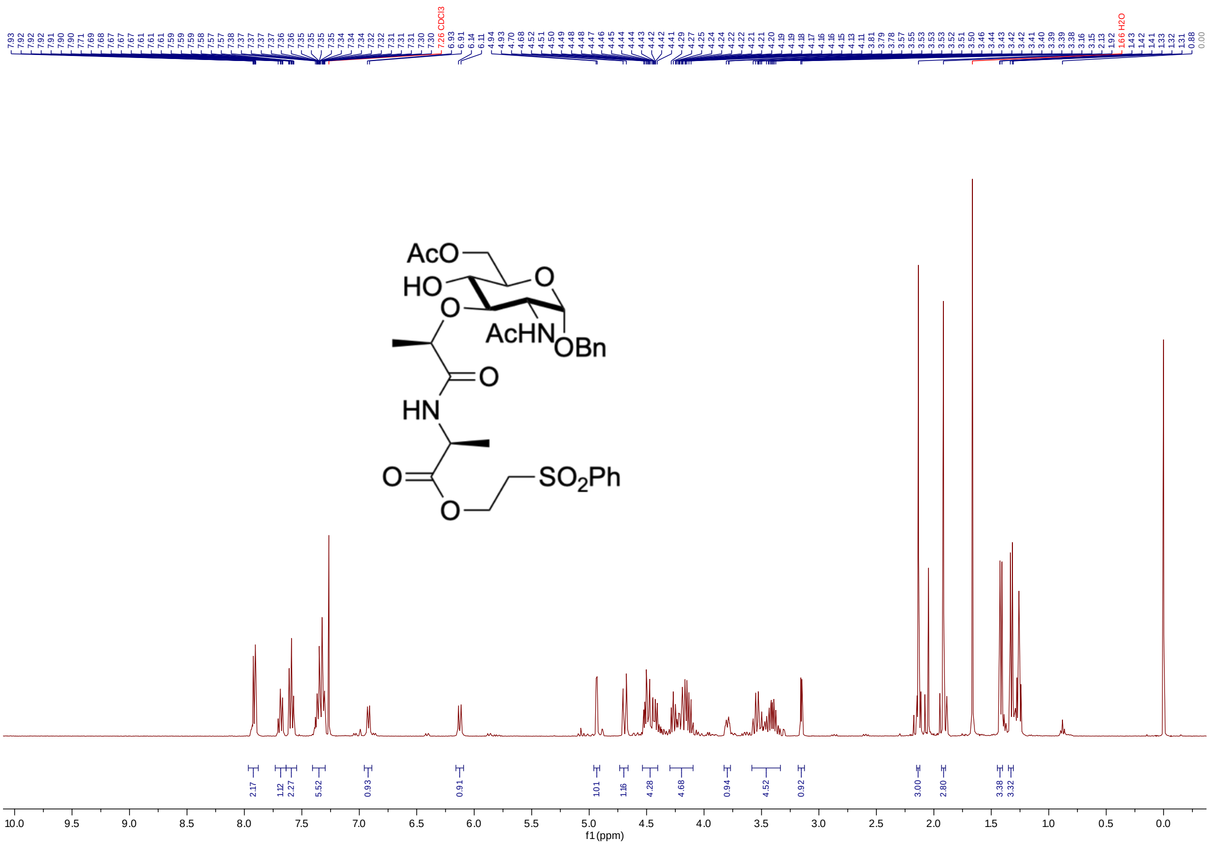

Figure S25: ^{1}H NMR spectrum (400 MHz, $CDCl_3$) of compound **2b**

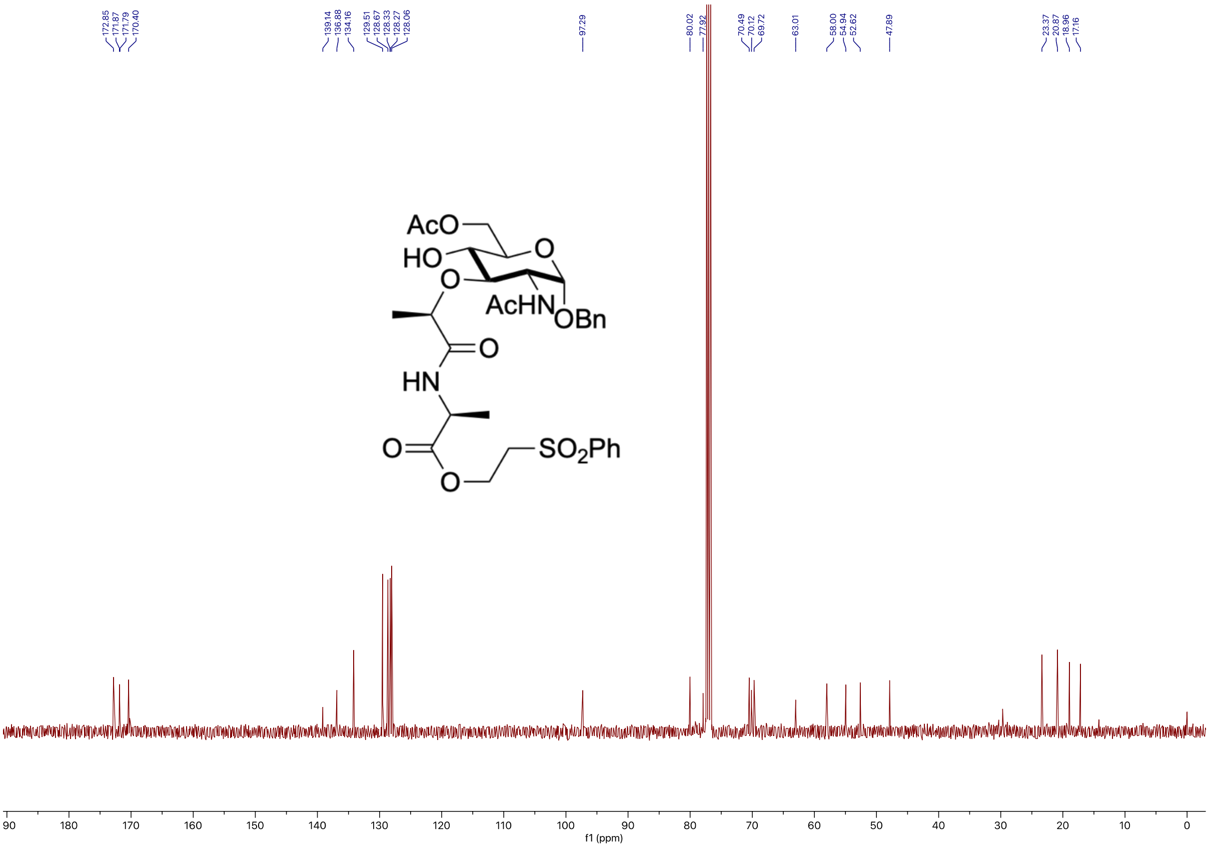

Figure S26: ^{13}C NMR spectrum (100 MHz, $CDCl_3$) of compound **2b**

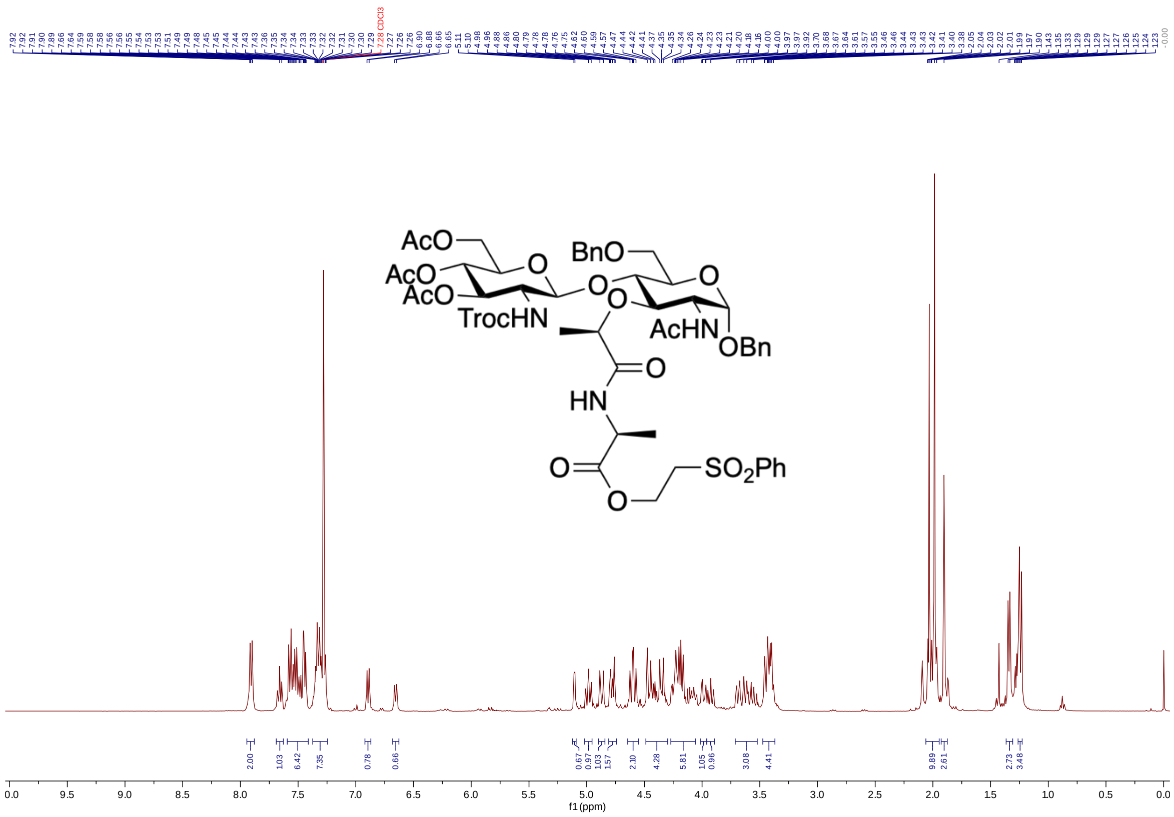

Figure S27: ^{1}H NMR spectrum (400 MHz, $CDCl_3$) of compound **3a**

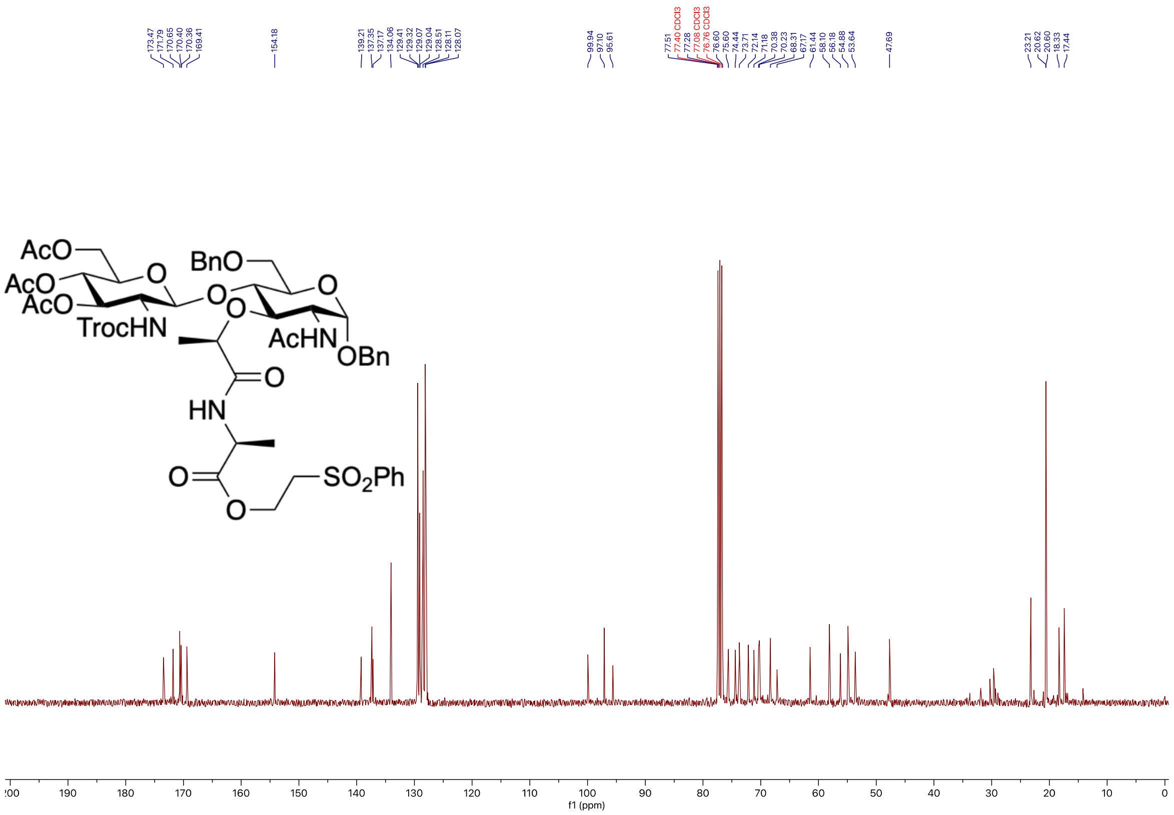

Figure S28: ^{13}C NMR spectrum (100 MHz, $CDCl_3$) of compound **3a**

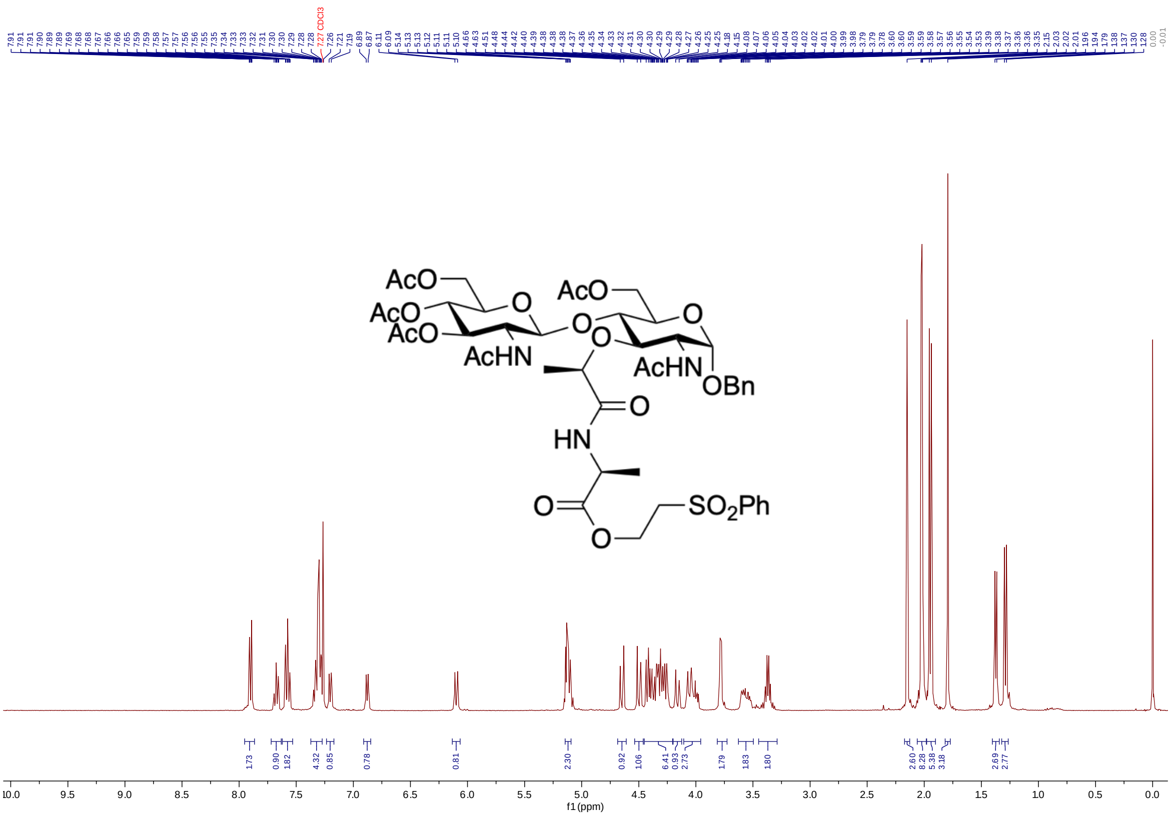

Figure S29: ^{1}H NMR spectrum (400 MHz, $CDCl_3$) of compound **4**

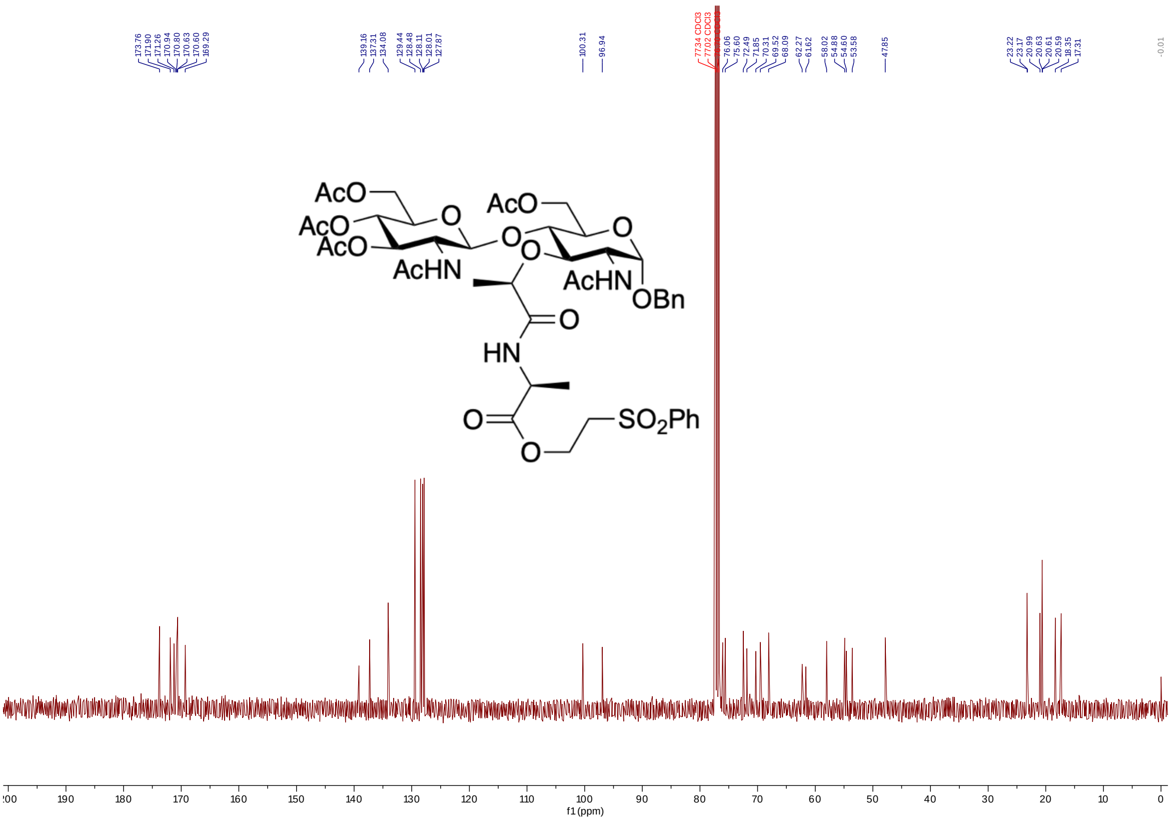

Figure S30: ^{13}C NMR spectrum (100 MHz, $CDCl_3$) of compound **4**

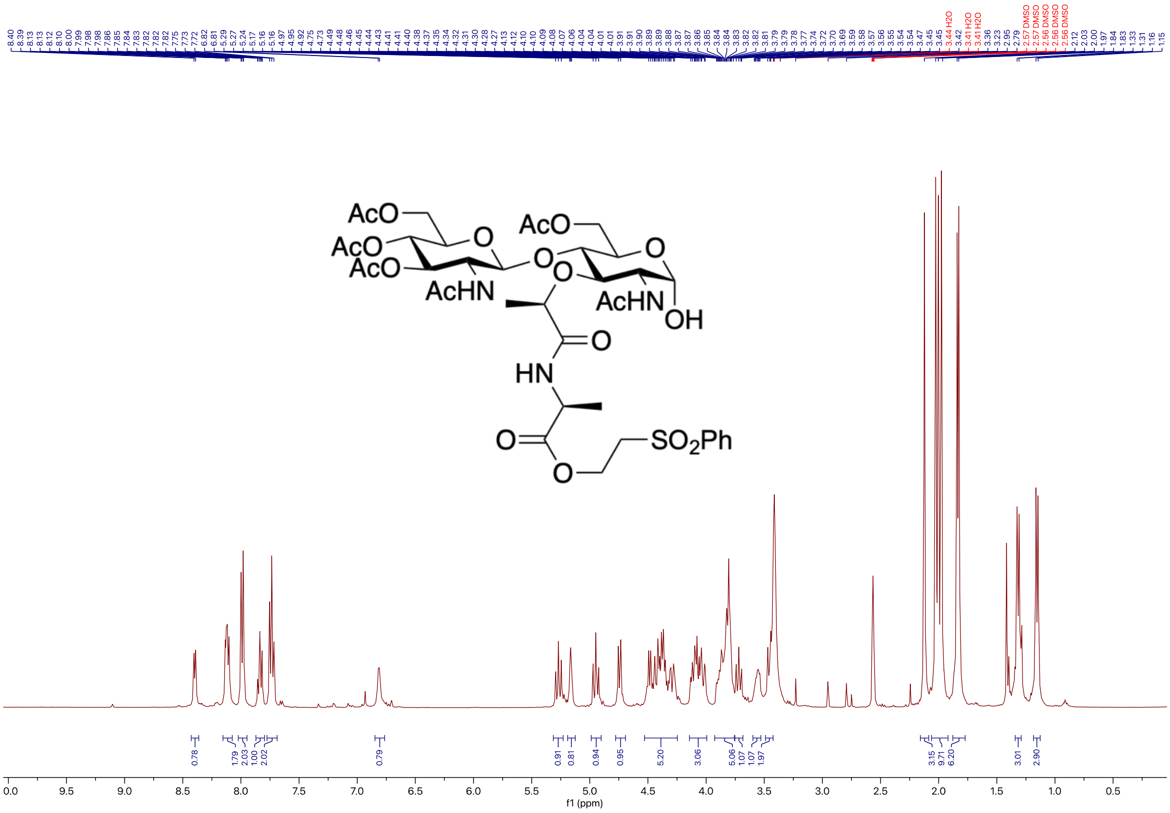

Figure S31: ^{1}H NMR spectrum (400 MHz, DMSO-d$_6$) of compound **5**

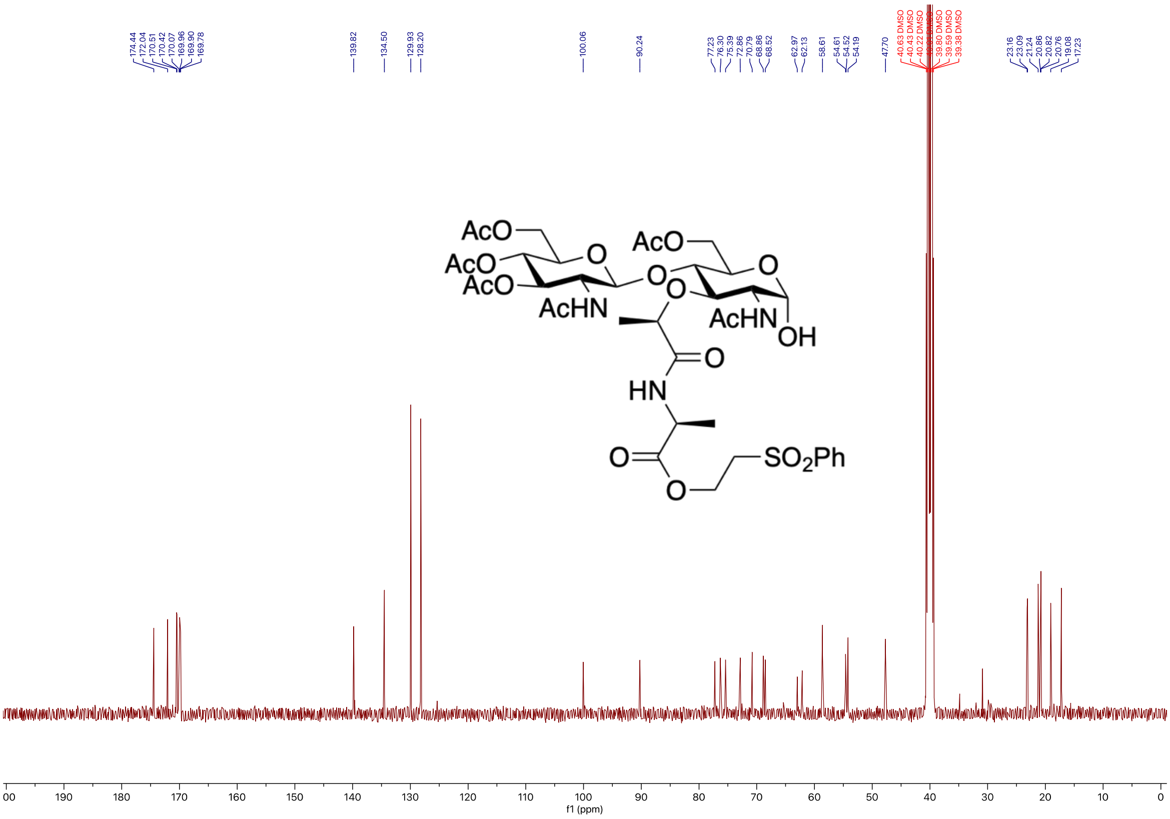

Figure S32: ^{1}H NMR spectrum (100 MHz, DMSO-d$_6$) of compound **5**

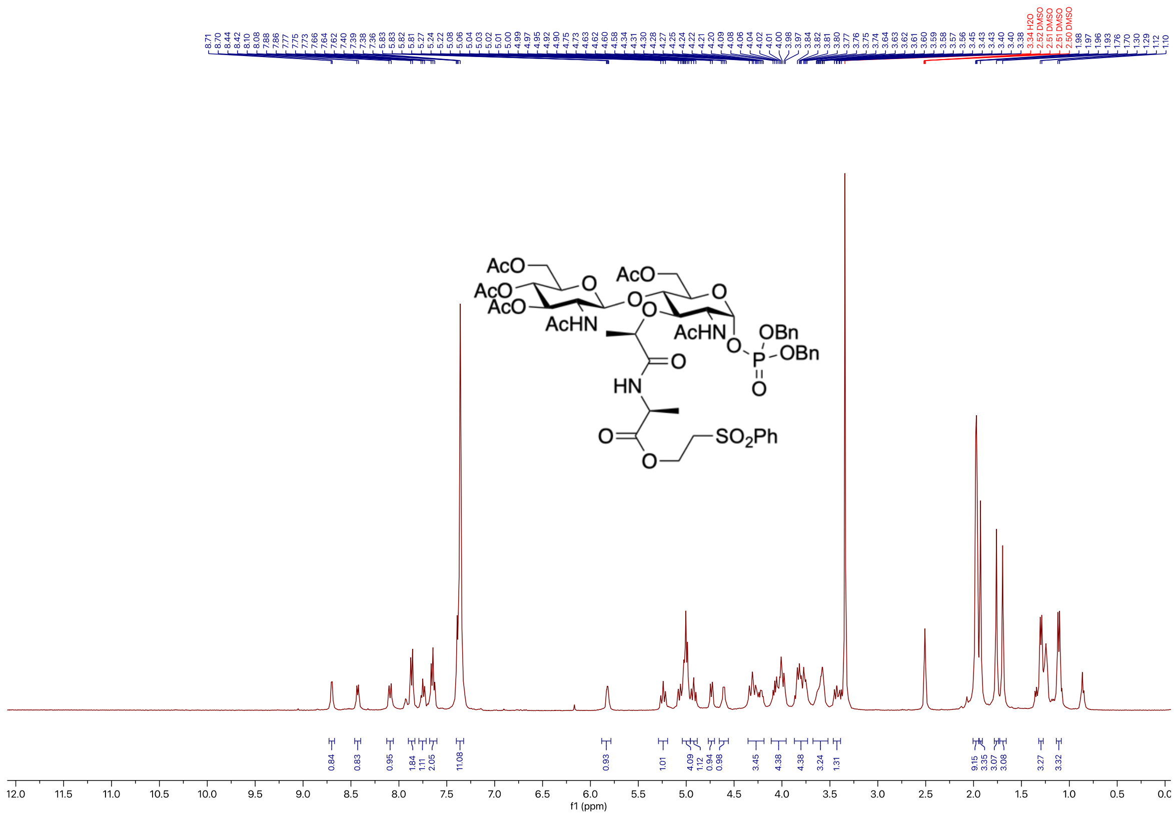

Figure S33: ^{1}H NMR spectrum (400 MHz, DMSO-d_6) of compound **6**

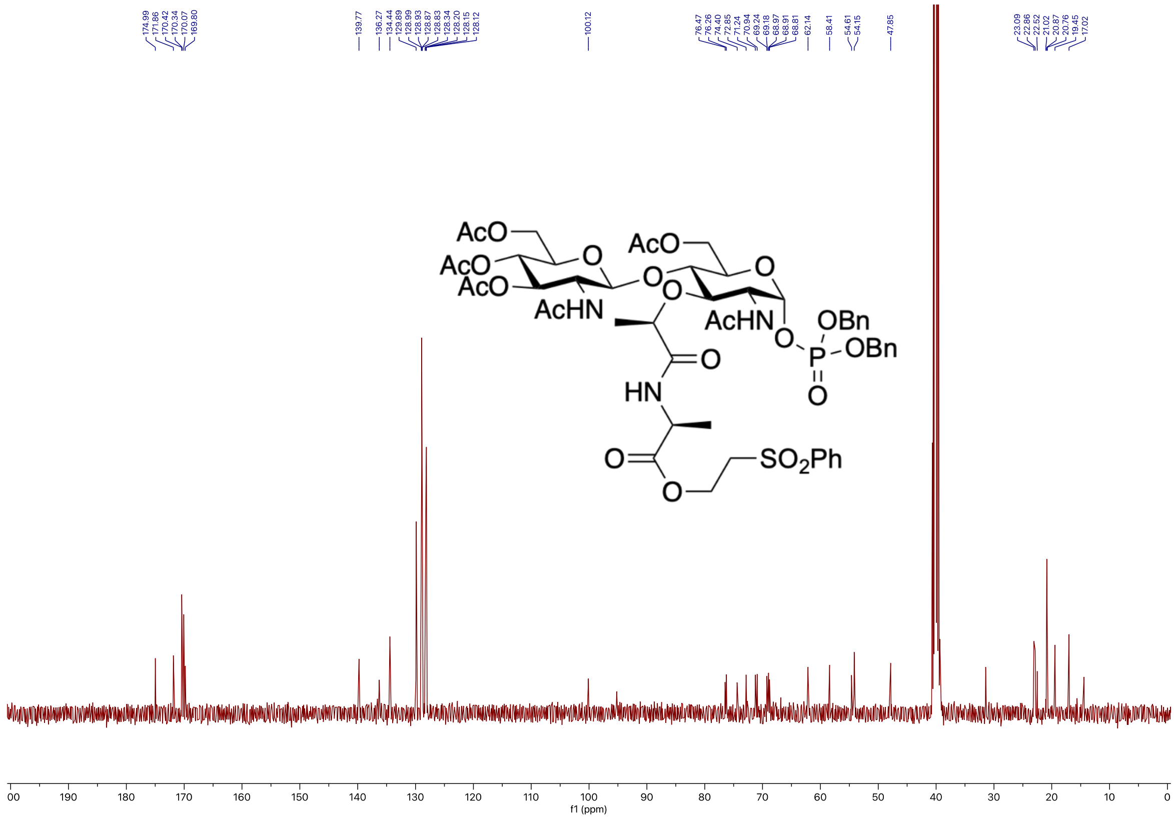

Figure S34: ^{13}C NMR spectrum (100 MHz, DMSO-d_6) of compound **6**

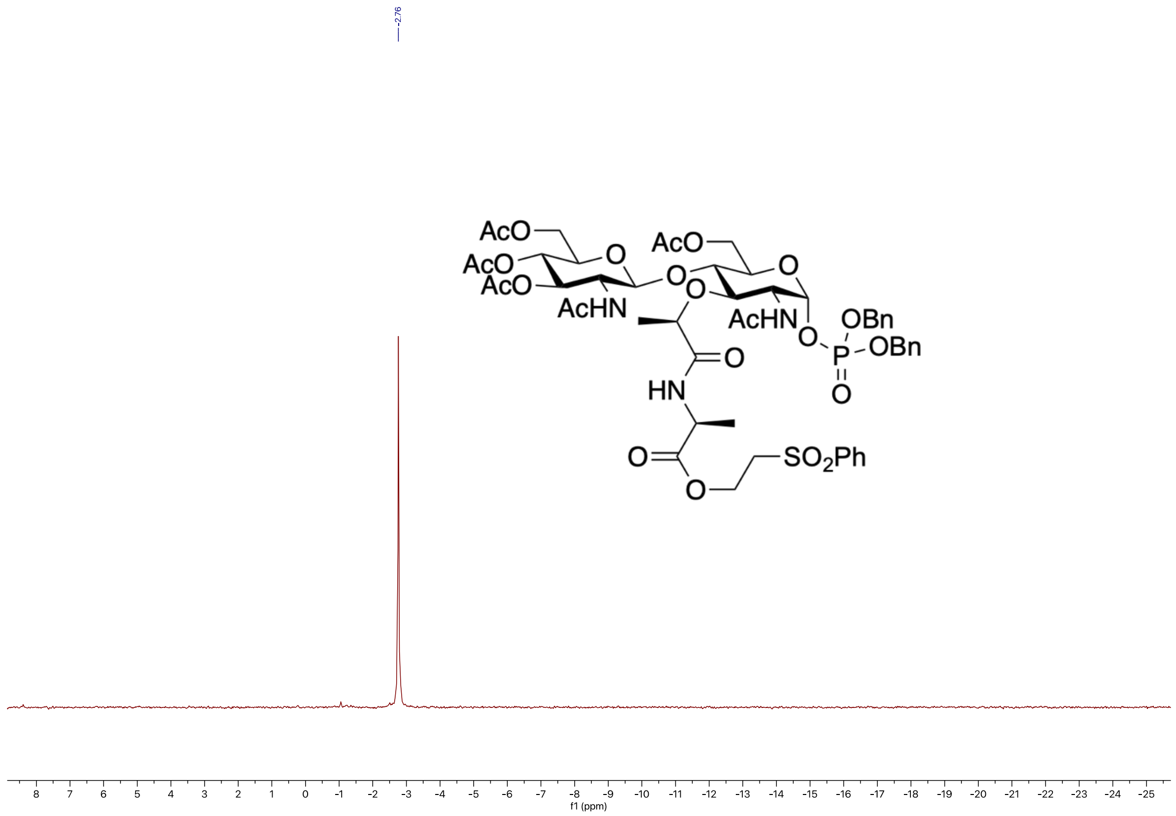

Figure S35: ^{31}P NMR spectrum (162 MHz, DMSO-d_6) of compound **6**

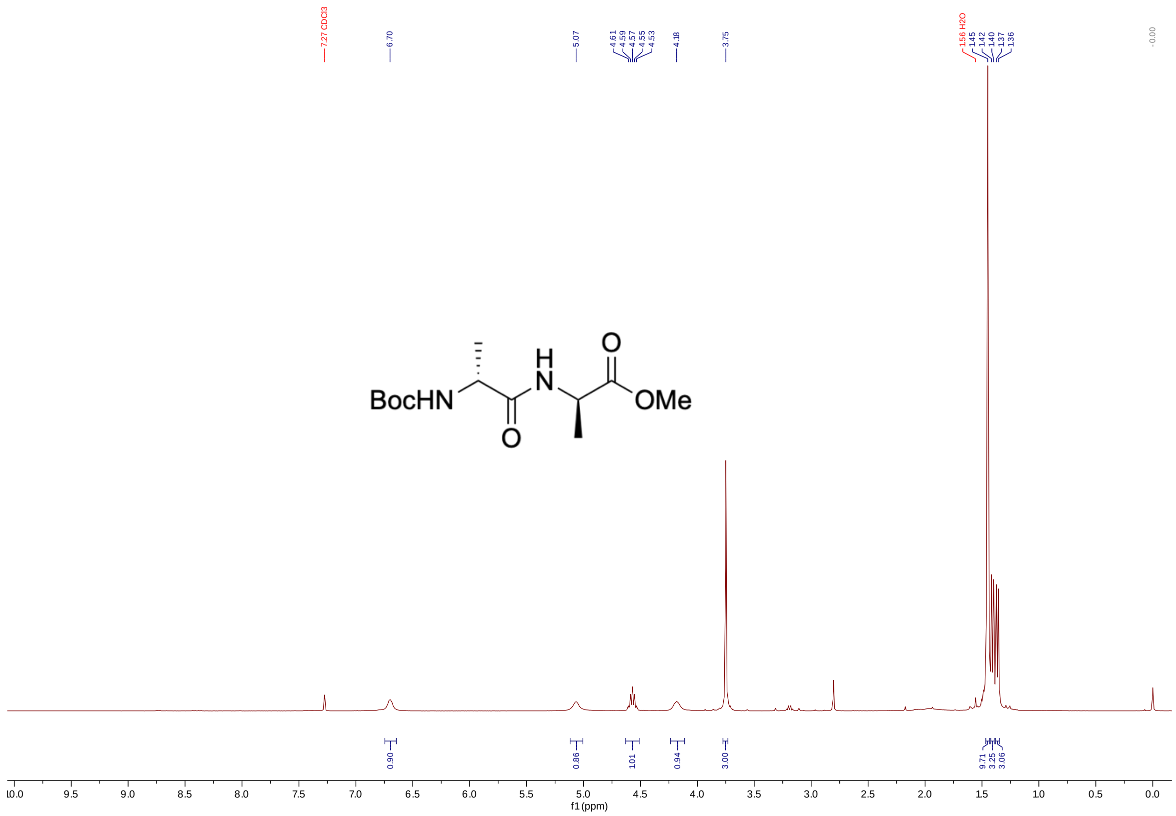

Figure S36: ^{1}H NMR spectrum (400 MHz, $CDCl_3$) of compound **S14**

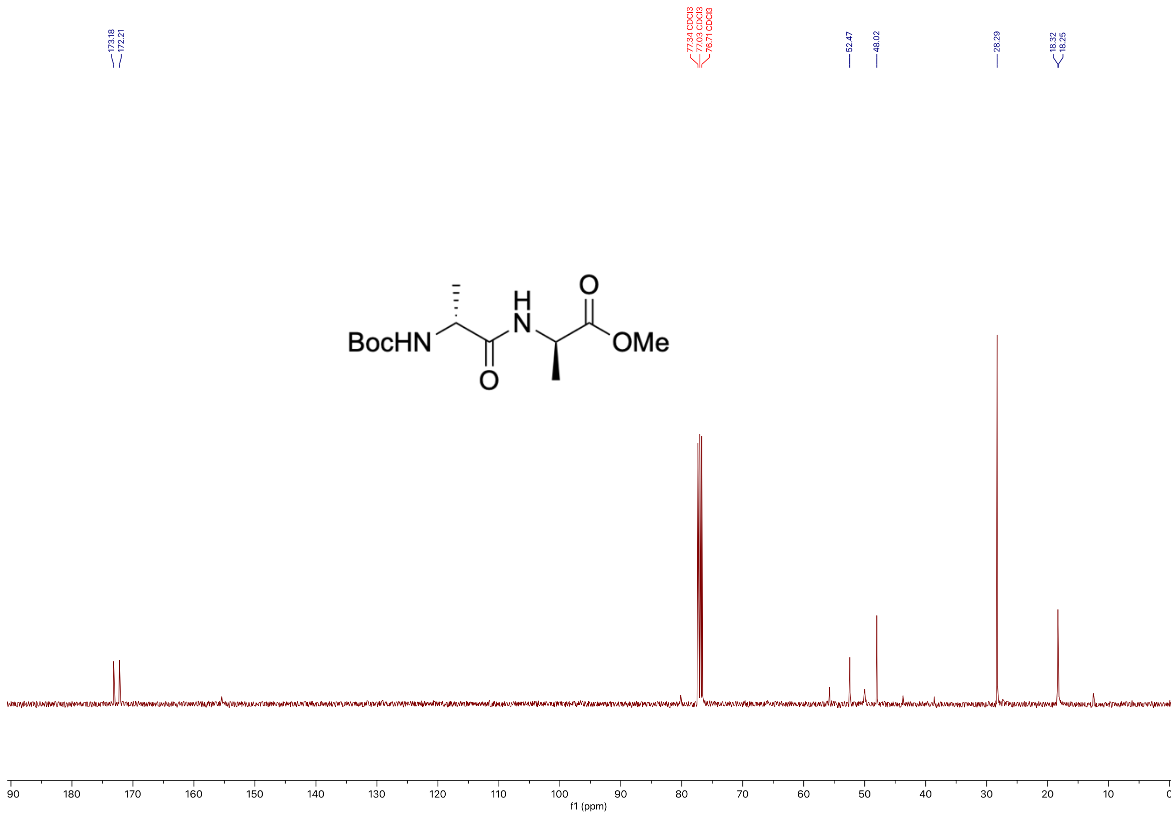

Figure S37: ^{13}C NMR spectrum (100 MHz, $CDCl_3$) of compound **S14**

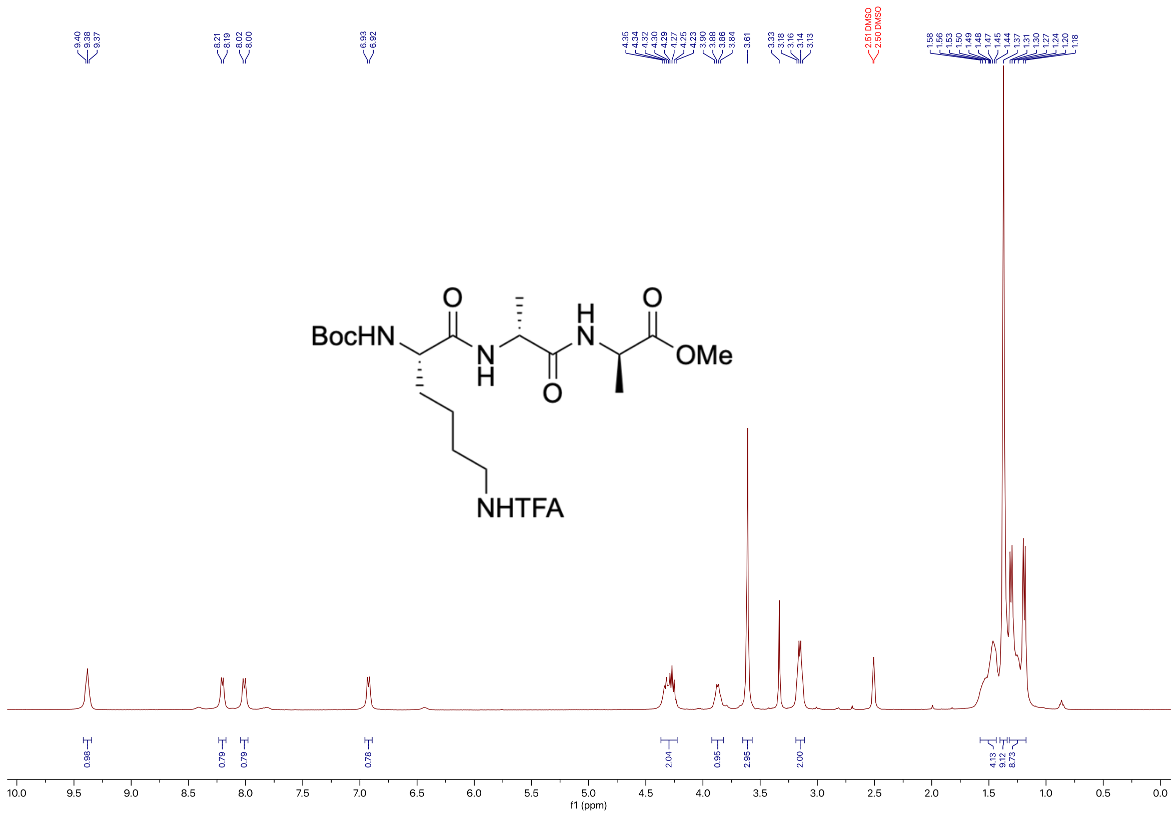

Figure S38: ^{1}H NMR spectrum (400 MHz, DMSO-d_6) of compound **S15**

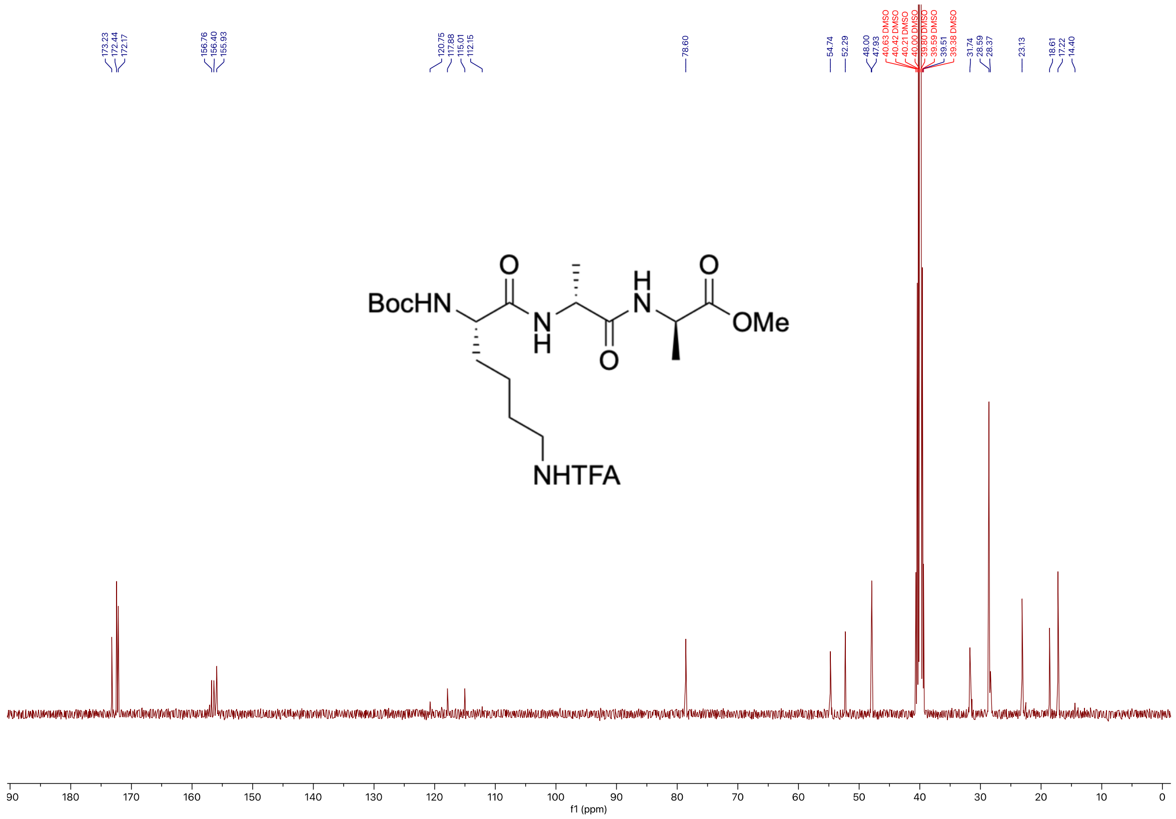

Figure S39: ^{13}C NMR spectrum (100 MHz, DMSO-d_6) of compound **S15**

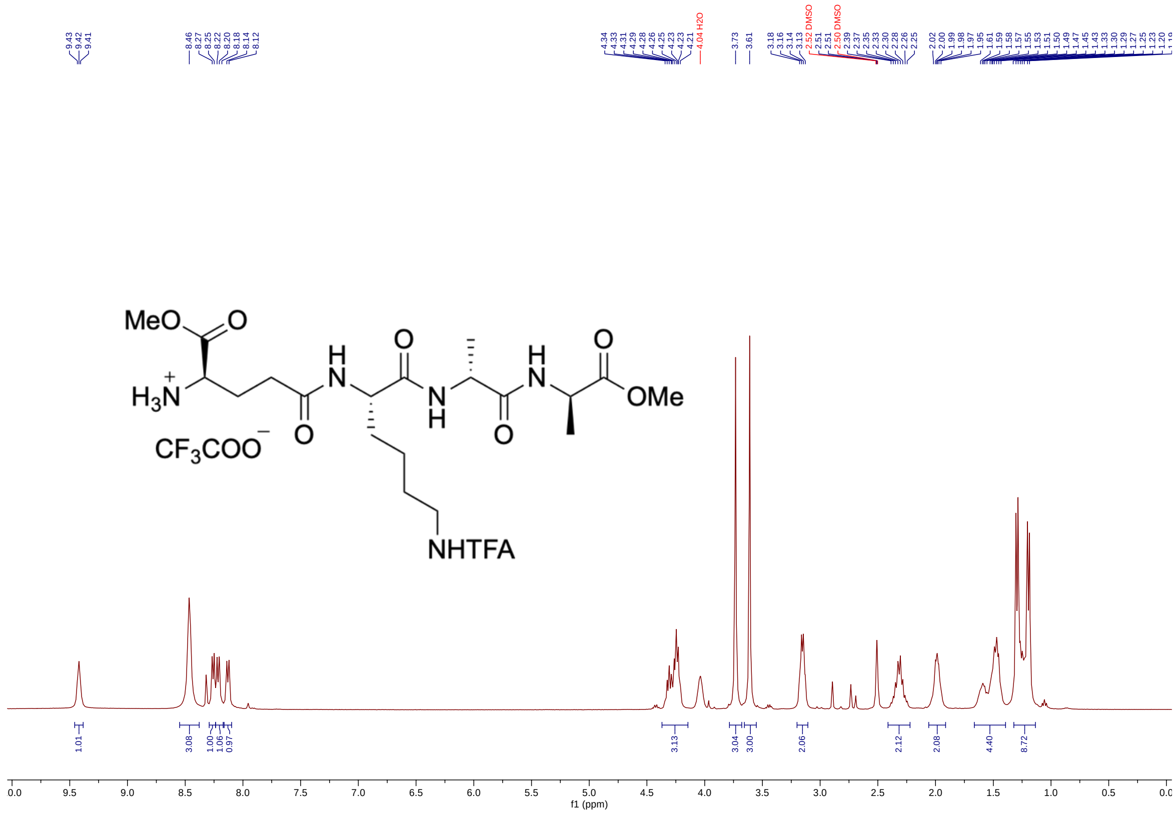

Figure S40: ^{1}H NMR spectrum (400 MHz, DMSO-d_6) of compound **S16**

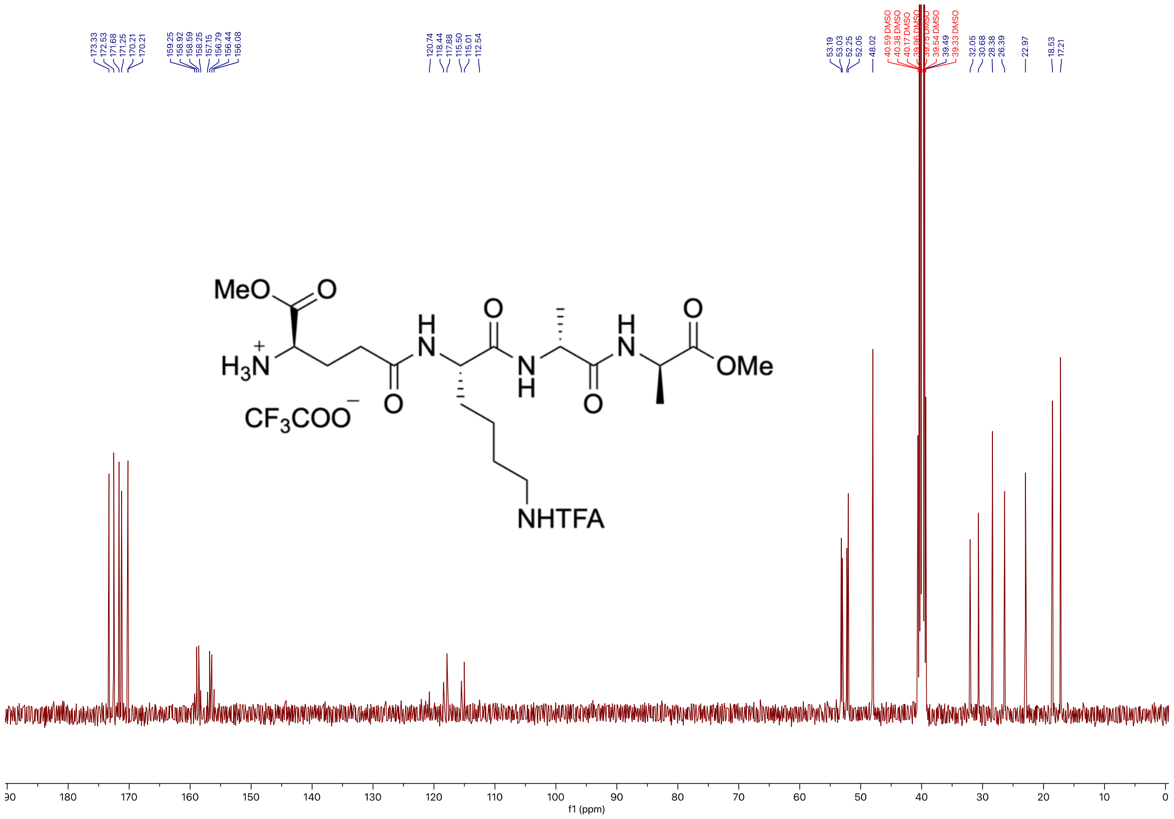

Figure S41: ^{1}H NMR spectrum (100 MHz, DMSO-d_6) of compound **S16**

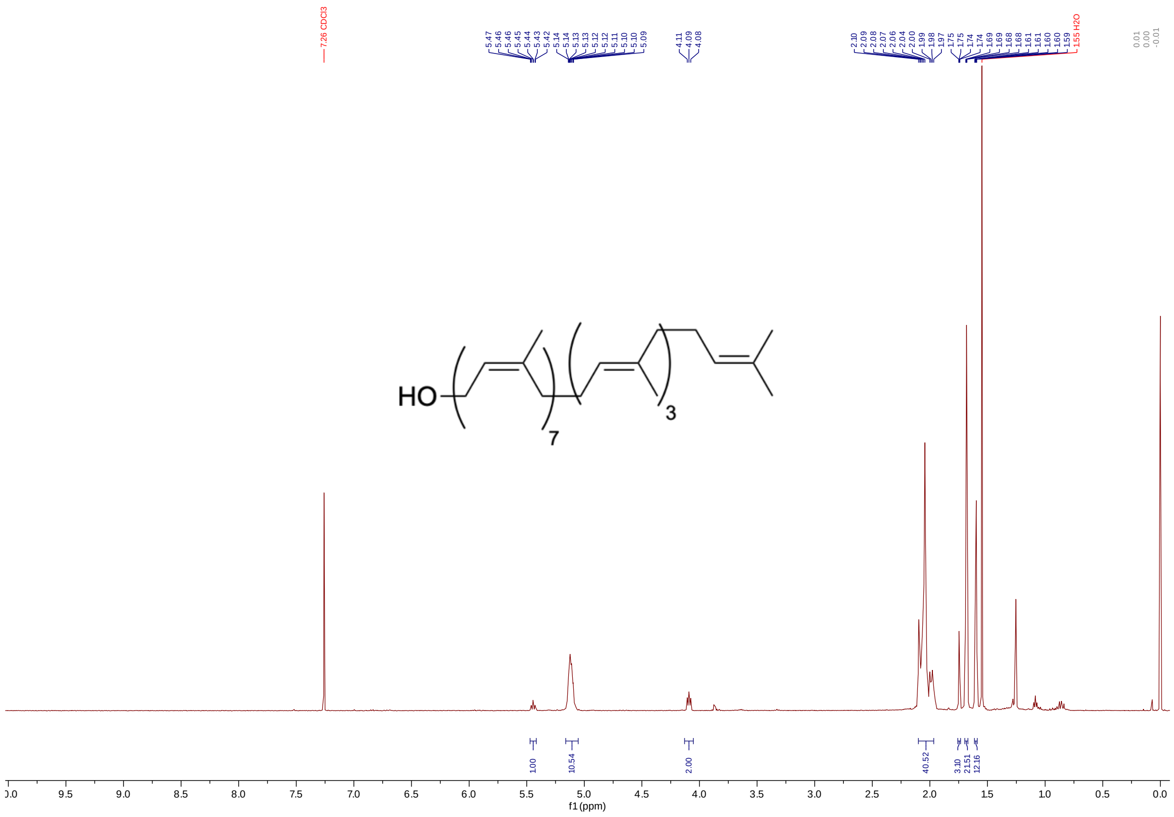

Figure S42: ^{1}H NMR spectrum (400 MHz, $CDCl_3$) of compound **S17**

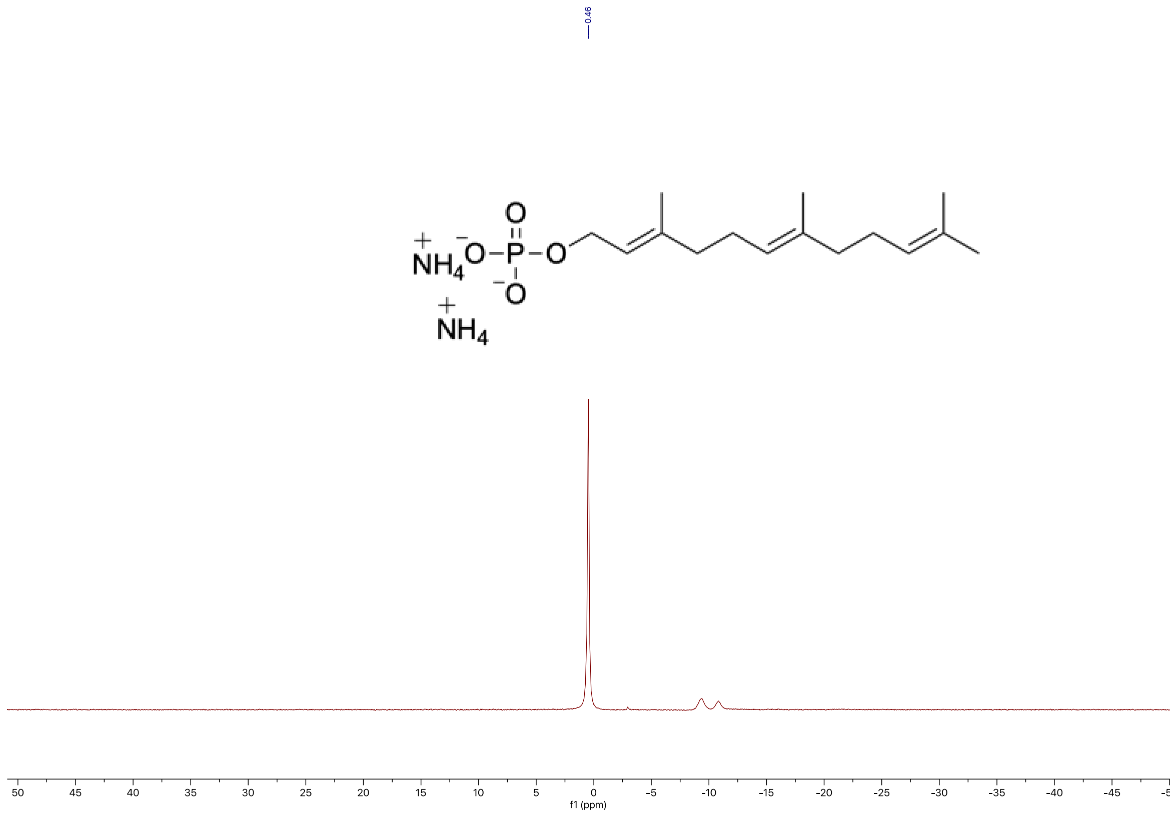

Figure S43: ^{31}P NMR spectrum (162 MHz, $CDCl_3$) of compound **S20**

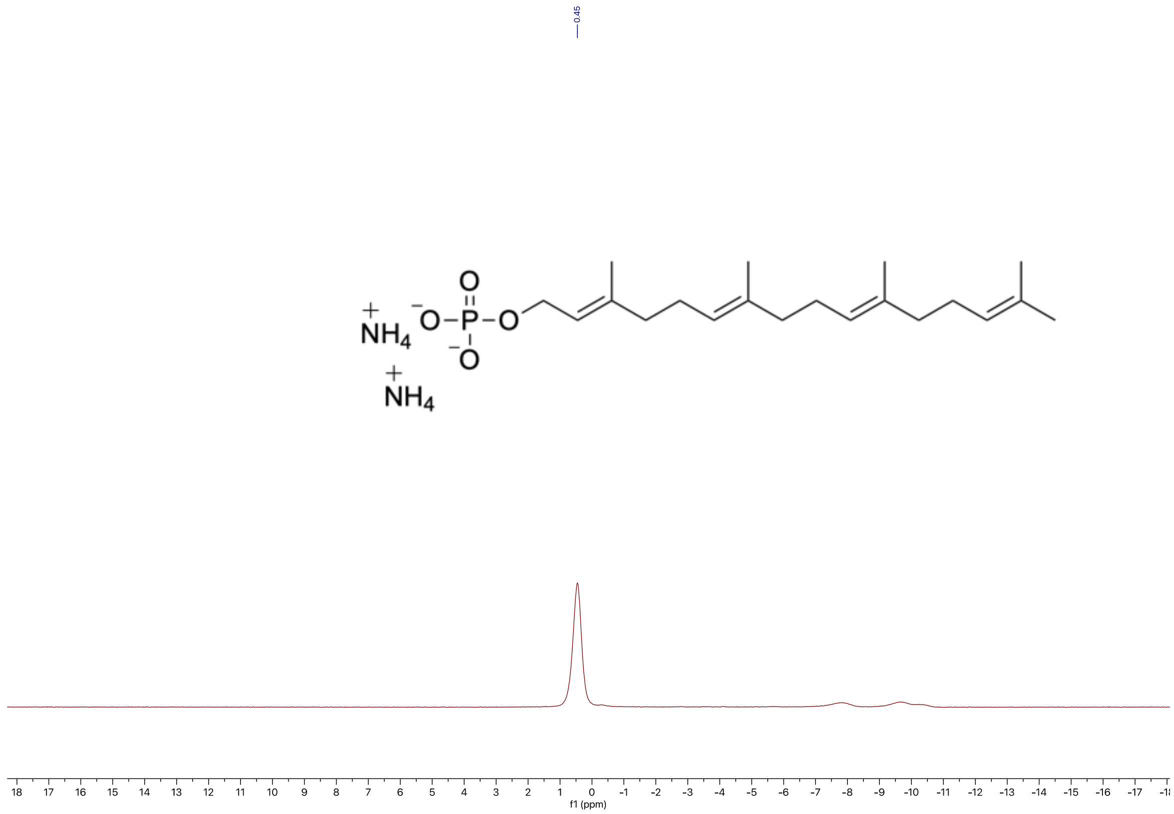

Figure S44: ^{31}P NMR spectrum (162 MHz, $CDCl_3$) of compound **S22**

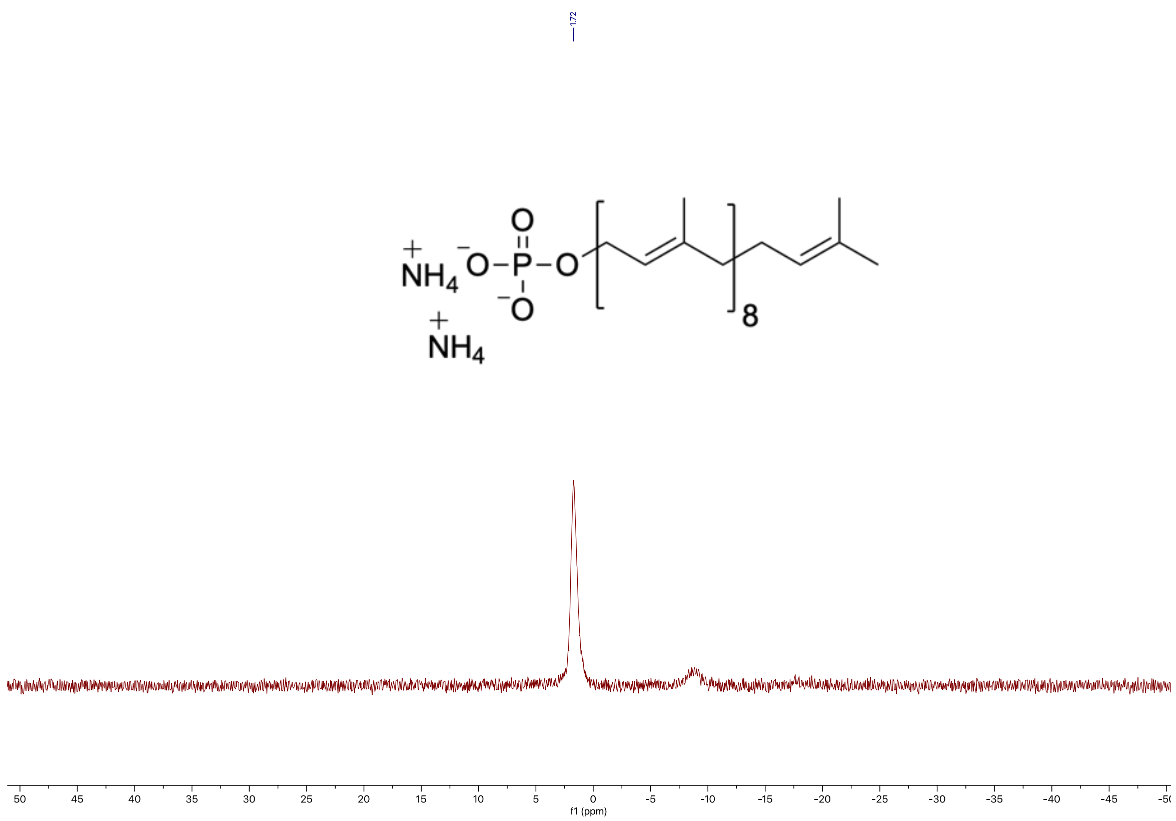

Figure S45: ^{31}P NMR spectrum (162 MHz, $CDCl_3$) of compound **S24**

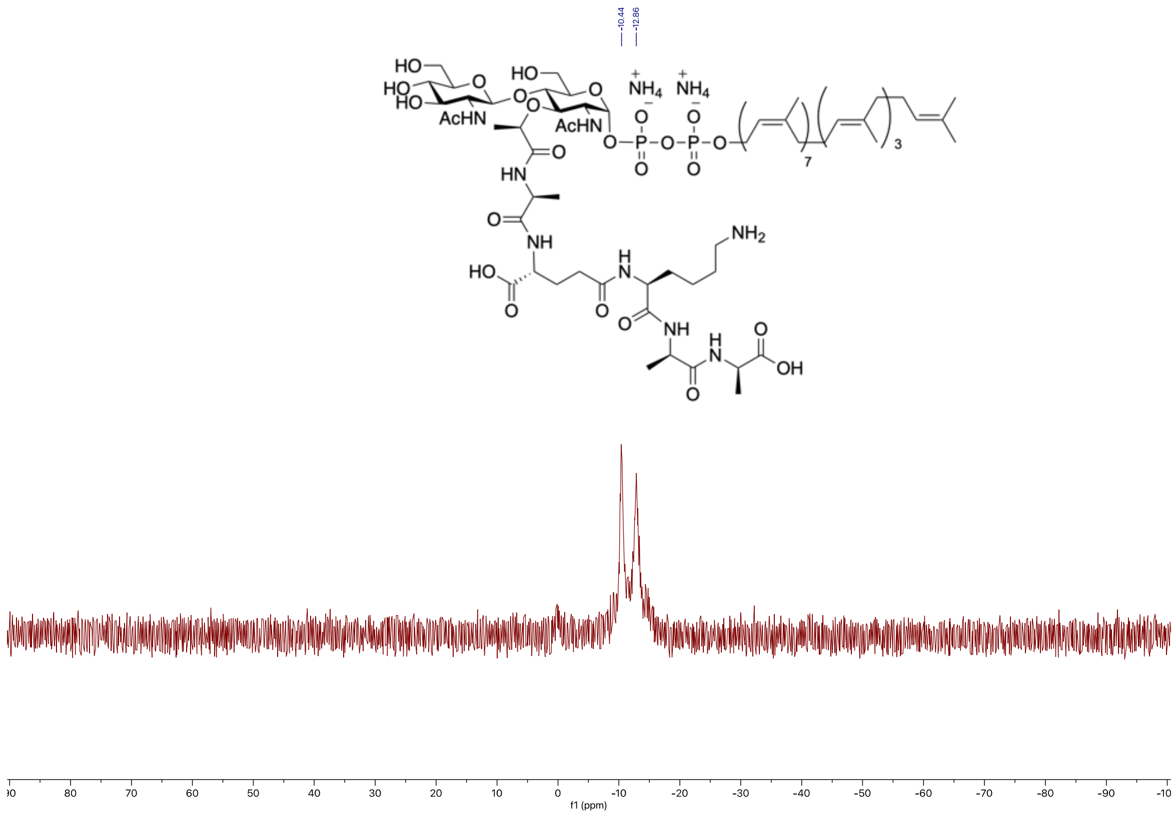

Figure S46: ^{31}P NMR spectrum (162 MHz, $CDCl_3$: CD_3OD 1:1) of compound **11**

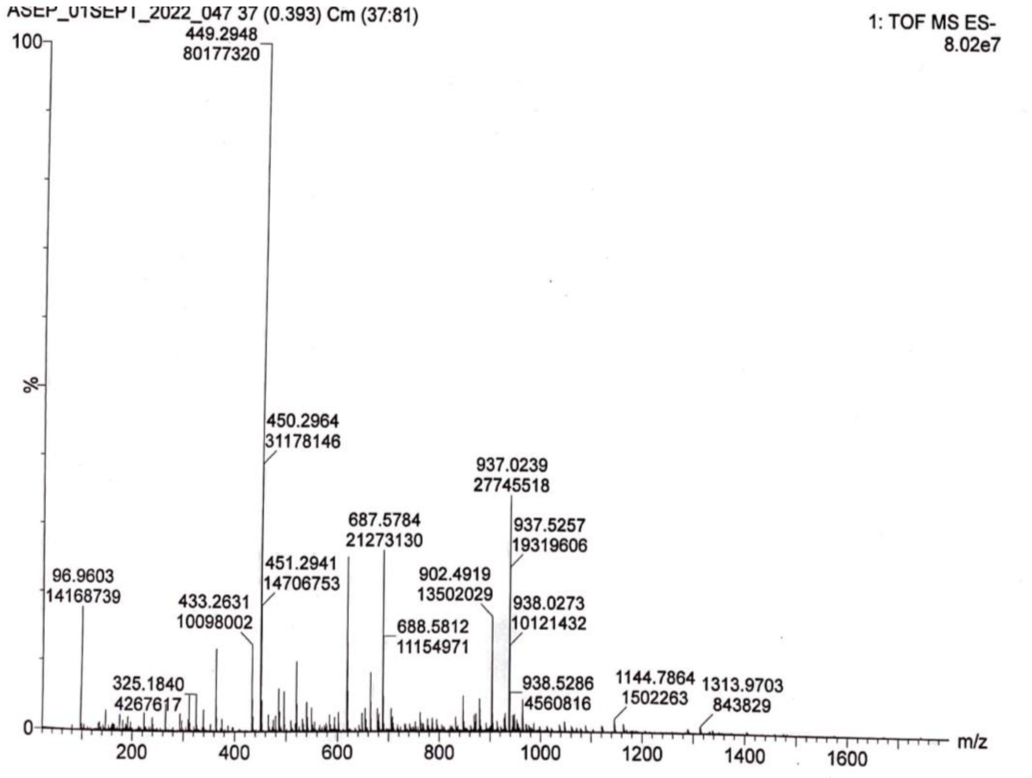

Figure S47: HRMS (ESI) of compound **11** (full spectrum)

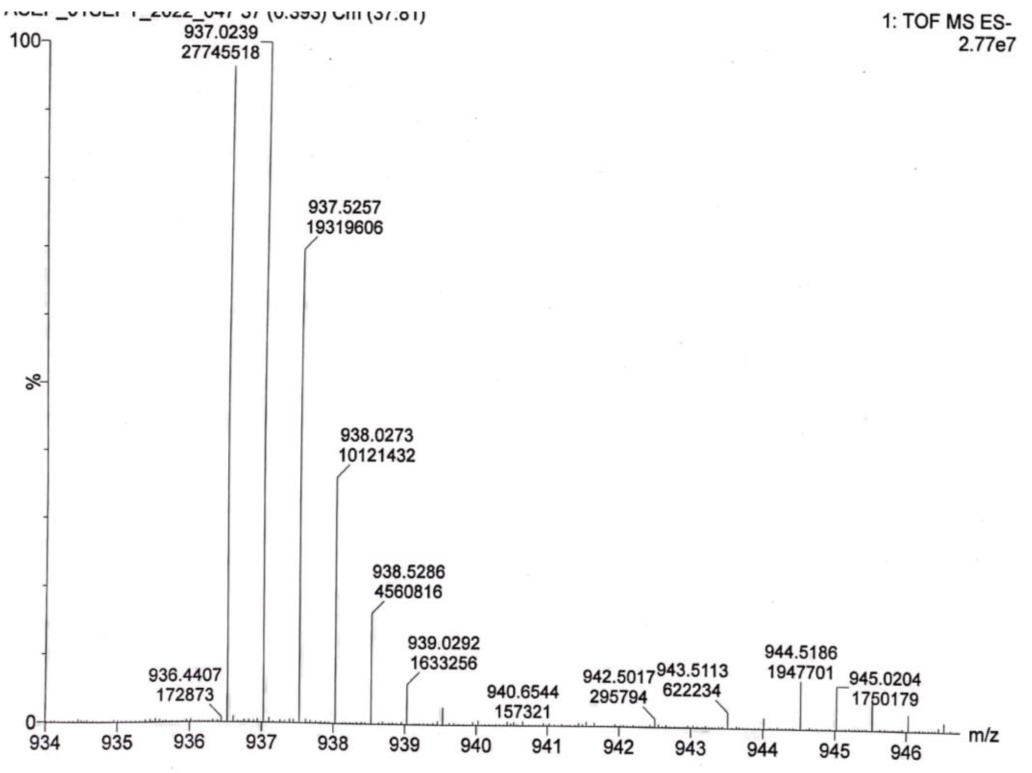

Figure S48: HRMS (ESI) of compound **11** (expanded; $[M-2H]^{-}$)

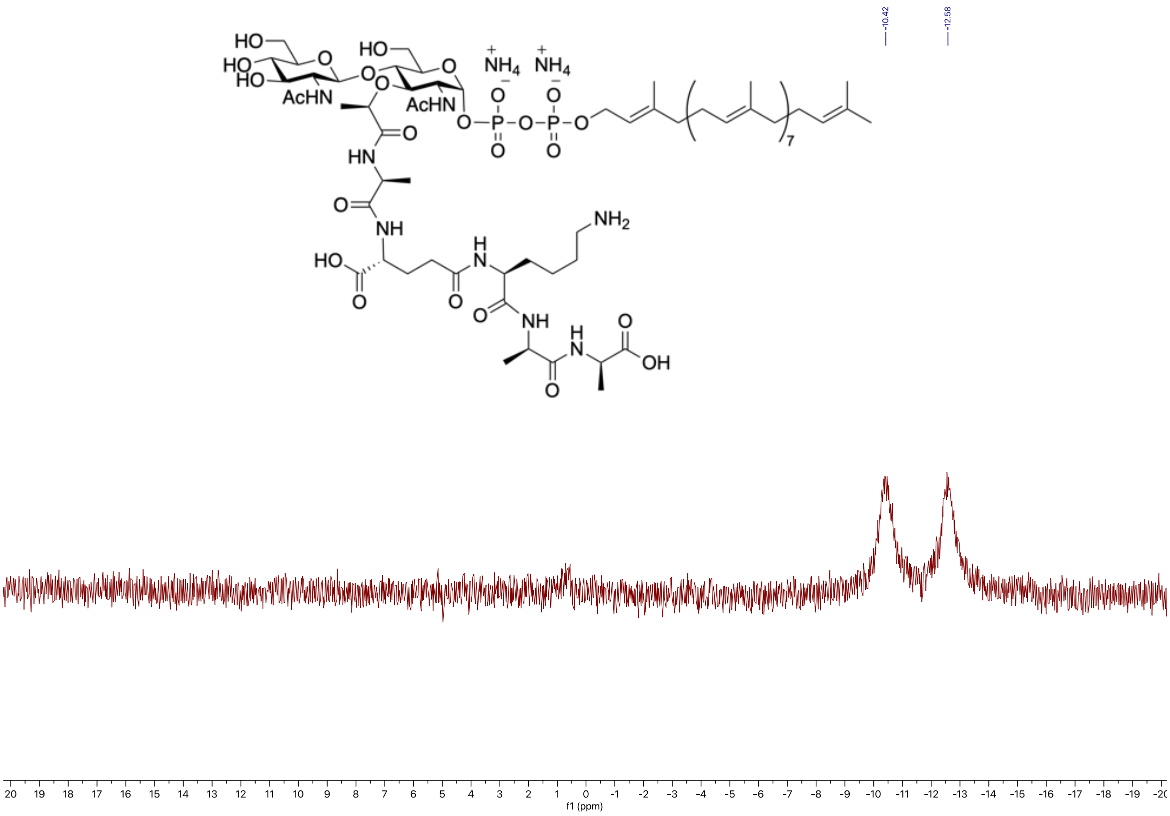

Figure S49: ^{31}P NMR spectrum (162 MHz, $CDCl_3$: CD_3OD 1:1) of compound **10**

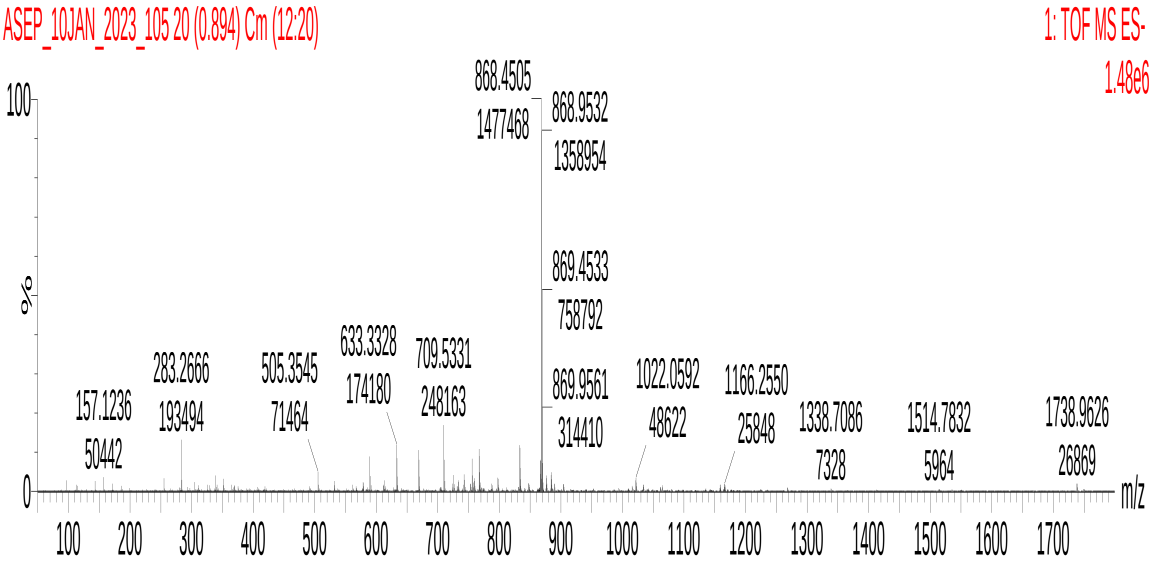

Figure S50: HRMS (ESI) of compound **10** (full spectrum)

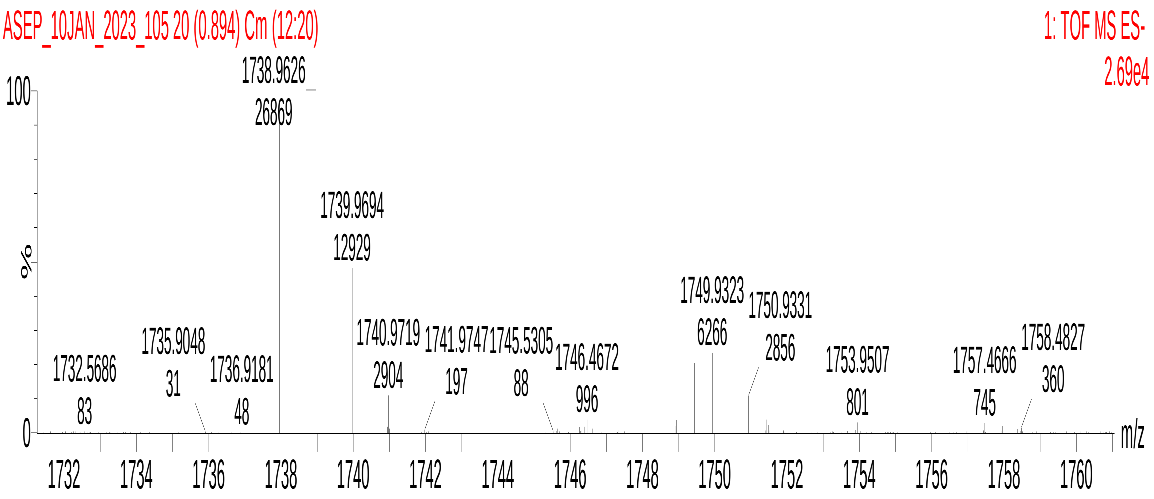

Figure S51: HRMS (ESI) of compound **10** (expanded; [M-H]$^-$)

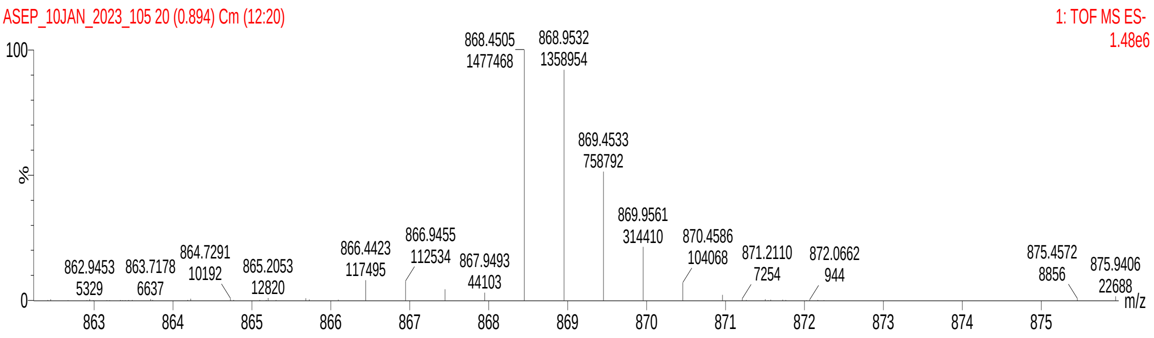

Figure S52: HRMS (ESI) of compound **10** (expanded; [M-2H]$^-$)

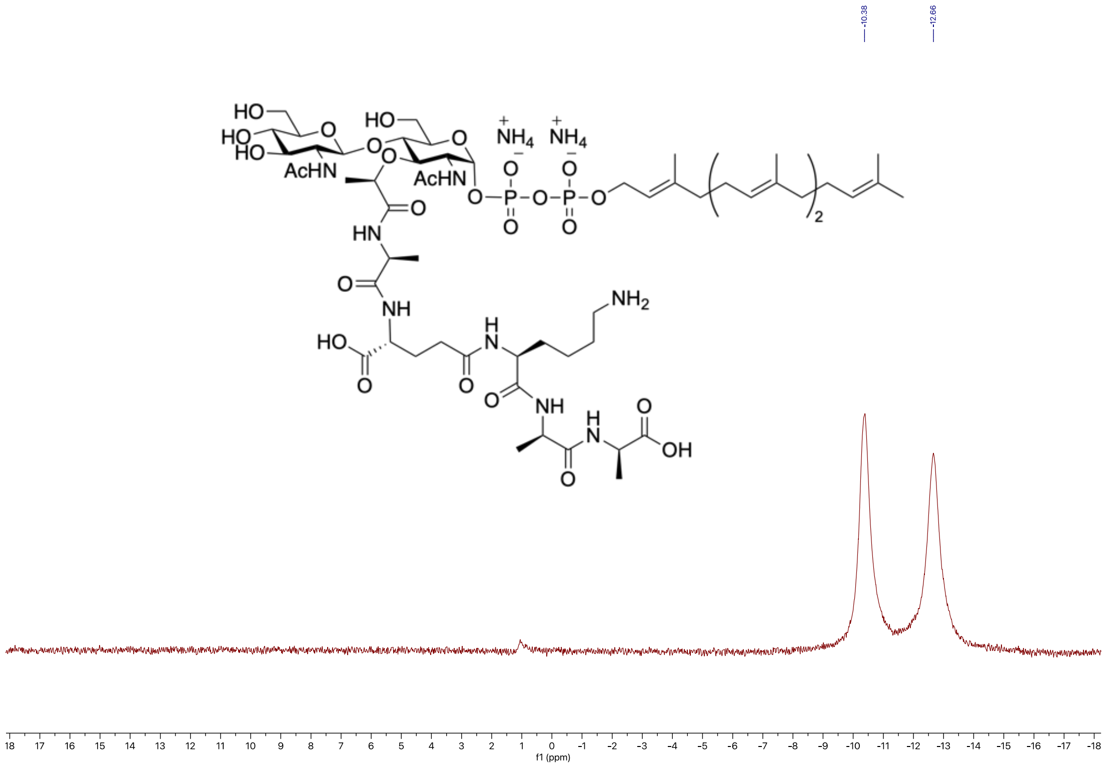

Figure S53: ^{31}P NMR spectrum (162 MHz, $CDCl_3$: CD_3OD 1:1) of compound **9**

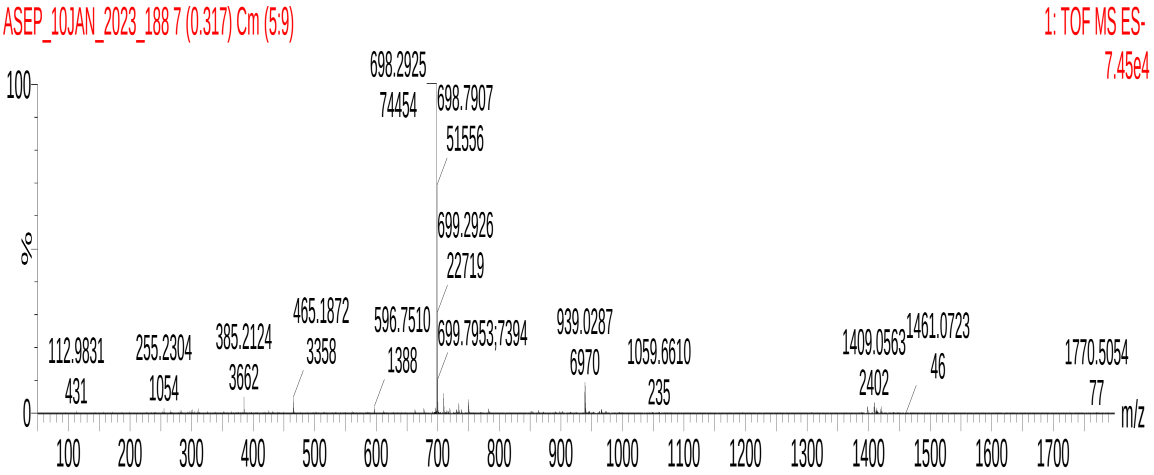

Figure S54: HRMS (ESI) of compound **9** (full spectrum)

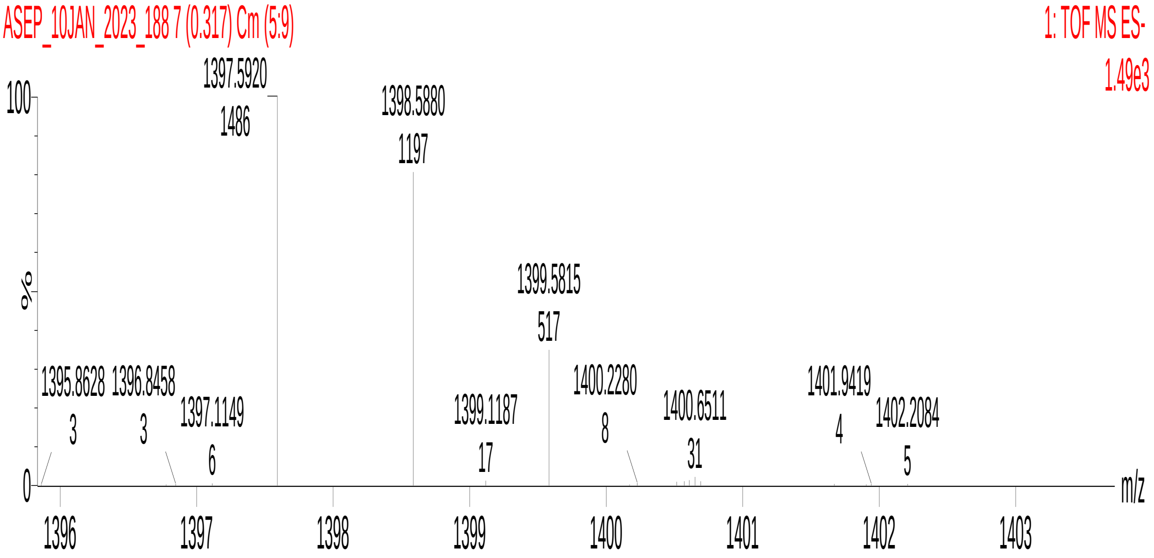

Figure S55: HRMS (ESI) of compound **9** (expanded; [M-H]$^-$)

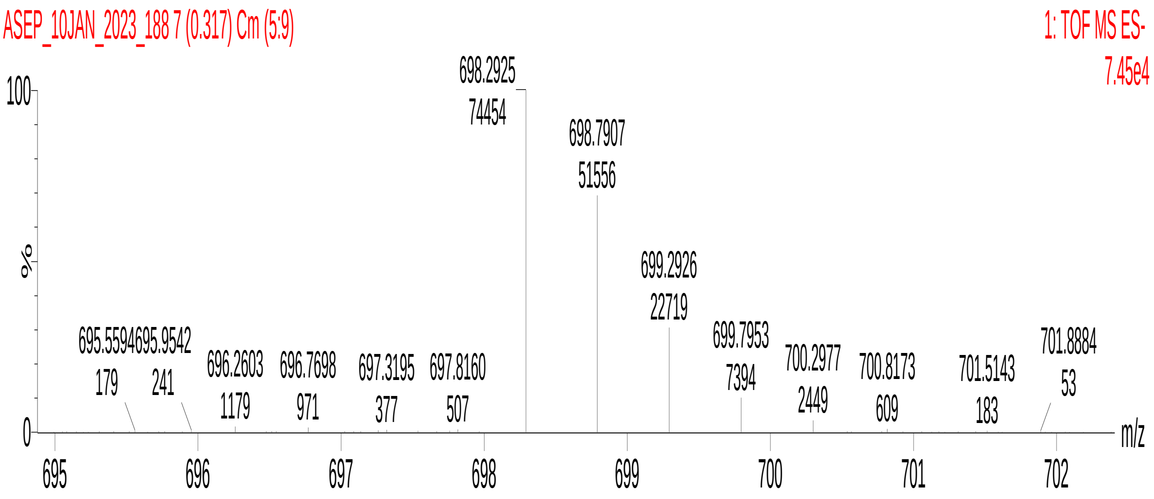

Figure S56: HRMS (ESI) of compound **9** (expanded; [M-2H]$^-$)

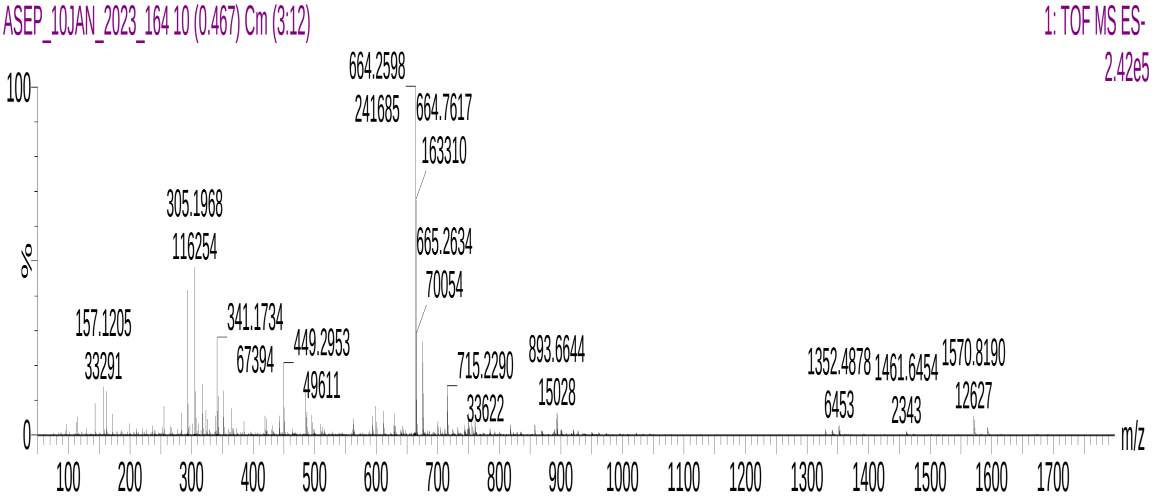

Figure S57: HRMS (ESI) of compound **8** (full spectrum)

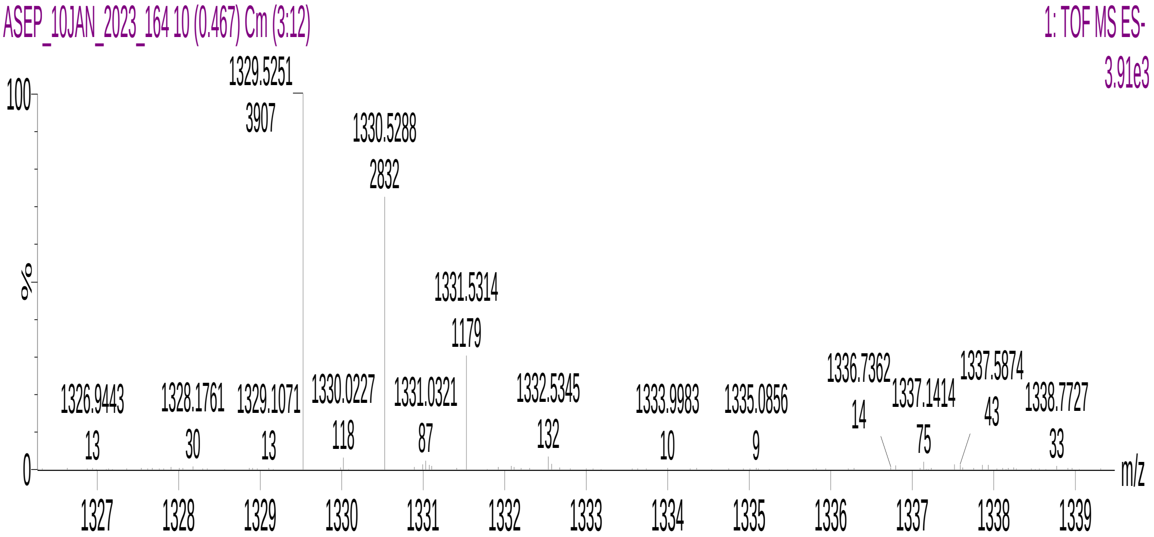

Figure S58: HRMS (ESI) of compound **8** (expanded; [M-H]$^-$)

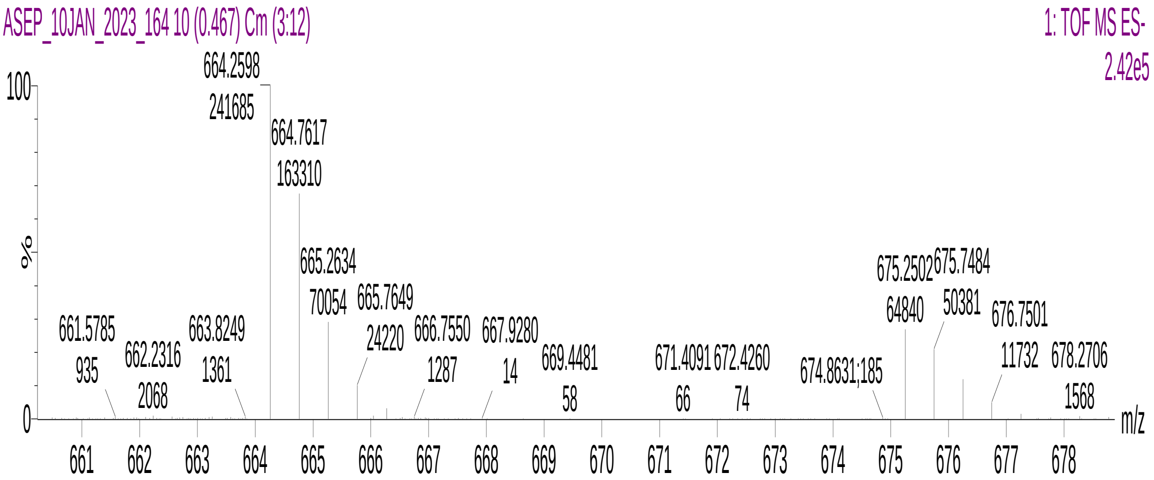

Figure S59: HRMS (ESI) of compound **8** (expanded; [M-2H]$^-$)
